# Radiation-Sensitive Nano-, Micro-, and Macro-Gels and Polymer Capsules for Use in Radiotherapy Dosimetry

**DOI:** 10.3390/ijms26146603

**Published:** 2025-07-10

**Authors:** Michał Piotrowski, Aleksandra Pawlaczyk, Małgorzata I. Szynkowska-Jóźwik, Piotr Maras, Marek Kozicki

**Affiliations:** 1 Department of Mechanical Engineering, Informatics and Chemistry of Polymer Materials, Faculty of Materials Technologies and Textile Design, Lodz University of Technology, 90-543 Lodz, Poland; michal.piotrowski@dokt.p.lodz.pl; 2 Institute of General and Ecological Chemistry, Faculty of Chemistry, Lodz University of Technology, 90-924 Lodz, Poland; aleksandra.pawlaczyk@p.lodz.pl (A.P.); malgorzata.szynkowska@p.lodz.pl (M.I.S.-J.); 3 Department of Radiotherapy Planning, Copernicus Hospital, 93-513 Lodz, Poland; p.maras@kopernik.lodz.pl

**Keywords:** Fricke gel dosimeter, radiotherapy dosimeter, 3D dosimeter, micro-gels, macro-gels, ionizing radiation

## Abstract

This work introduces an original approach to the manufacturing of ionizing radiation-sensitive systems for radiotherapy applications—dosimetry. They are based on the Fricke dosimetric solution and the formation of macro-gels and capsules, and nano- and micro-gels. The reaction of ionic polymers, such as sodium alginate, with Fe and Ca metal ions is employed. Critical polymer concentration (c*) is taken as the criterion. Reaction of ionic polymers with metal ions leads to products related to c*. Well below c*, nano- and micro-gels may form. Above c*, macro-gels and capsules can be prepared. Nano- and micro-gels containing Fe in the composition can be used for infusion of a physical gel matrix to prepare 2D or 3D dosimeters. In turn, macro-gels can be formed with Fe ions crosslinking polymer chains to obtain radiation-sensitive hydrogels, so-called from wall-to-wall, serving as 3D dosimeters. The encapsulation process can lead to capsules with Fe ions serving as 1D dosimeters. This work presents the concept of manufacturing various gel structures, their main features and manufacturing challenges. It proposes new directions of research towards novel dosimeters.

## 1. Introduction

Radiotherapy is a technique of cancer treatment using ionizing radiation. It is performed with the aid of the most modern irradiation techniques, such as IMRT (intensity modulated radiation therapy), VMAT (volumetric modulated arc therapy), SRT (stereotactic radiation therapy), SRS (stereotactic radiosurgery), 4DRT (four-dimensional radiation therapy, also called respiratory gating), and internal radiotherapy (brachytherapy). These techniques allow the elimination of even small tumors while preserving healthy tissue [1,2,3]. Their introduction is associated with higher expectations regarding the methods of measuring ionizing radiation (dosimetry) used both to test sources of ionizing radiation and equipment and to verify calculations made by treatment planning systems (TPS). In response, scientists have began research into 3D and 4D dosimetry techniques, which have been developed over more than forty years to adapt them to patient irradiation techniques.

The first proposal of 3D dosimeters concerned the infusion of a physical gel matrix with a well-known Fricke dosimeter [4] and the measurement of changes after irradiation related to the transformation of Fe^+2^ into Fe^+3^ inside the dosimeter using magnetic resonance imaging (MRI) [5]. This was the driving force behind many proposals of other 3D dosimeters [6,7], such as the following: (i) Fricke radiochromic gel dosimeters, for which the conversion of Fe ions was combined with the reaction with, for example, xylenol orange to form colored complexes [8,9,10,11,12,13,14,15]; such a dosimeter can be measured by optical computed tomography (OCT). (ii) Polymer gel dosimeters with vinyl monomers undergoing polymerization and crosslinking, which affects the density of the dosimeter; such a dosimeter can be measured by MRI (and MR-linac), computed tomography (CT and CT-linac), OCT, and ultrasonography (USG) [16,17,18,19,20,21,22,23,24,25,26,27,28]. (iii) Radiochromic gel dosimeters employing various color precursors such as tetrazolium salts, leuco-dyes (e.g., leuco crystal violet, leuco malachite green), and potassium iodine; they act upon conversion of a colorless or colored compound into a product of a different color than the substrate and can be measured by OCT [29,30,31,32]. (iv) Radiochromic resin/plastic dosimeters acting similar to radiochromic gel dosimeters [33,34,35]. (v) Lung-imitating dosimeters based on polymer gel dosimeters; they can be measured with MRI [36,37,38]. (vi) Deformable radiochromic dosimeters that can change their shape under stress, adapting to the movement of the organ and the changing shape of the organ, e.g., during breathing; they can be measured by OCT [39,40]. (vii) Radiofluorogenic gel dosimeters which, after irradiation with ionizing radiation, fluoresce under the influence of ultraviolet light and thus can be measured with the FluoroTome scanner [41,42,43]. (viii) Recently proposed combined dosimeters that simulate different tissues in one vial, such as lungs and muscles; they are based on polymer gel dosimeters (unpublished results by the authors) or radiochromic gel dosimeters, and possible measurements are by OCT, MRI, or CT [44]. Data resulting from 3D measurements of dosimeters can be processed using dedicated software packages [25,45]. In this work, however, the emphasis is on Fricke radiochromic gel dosimetry.

Fricke gel dosimetry aroused great interest after the introduction of coloring substances into the dosimeter and the possibility of 3D measurement using OCT [8,9,10,11,12,13,14,15,46,47]. However, the most unfavorable effect for 3D Fricke dosimetry is the loss of integrity of the 3D dose distribution over time after non-uniform irradiation. It is assessed by measuring the diffusion coefficient [48] of Fe^+3^ or Fe^+3^ complexes with, for example, xylenol orange (XO). The diffusion coefficient should be equal or close to 0 mm^2^ h^−1^ so that the dosimeter can reliably measure the 3D dose distribution by OCT or MRI. Unfortunately, it seems that even the force of gravity may affect the diffusion of [XO-Fe]^+3^ complexes formed after irradiation [15]. Therefore, numerous proposals to reduce the diffusion effect have been discussed (consult Table 2 in [15]), of which only one, the Fricke nanocomposite gel (NC-FG) [49,50], seems to be superior. An interesting proposal to improve Fricke gels by using complex networks of hydrogels based on poly(vinyl alcohol) has been presented elsewhere [51].

In this work, the Fricke gel dosimeter is studied with two gel matrices: a commonly used natural gelatine polymer and a synthetic co-polymer of poly(ethylene oxide)-*block*-poly(propylene oxide)-*block*-poly(ethylene oxide) (Pluronic F-127). Pluronic F-127 has been introduced into 3D radiotherapy dosimeter formulations due to its remarkable properties, such as the ability to prepare a gel dosimeter at low temperatures due to the phase behavior of Pluronic F-127 [52], high degree of transparency and colorlessness of gels, non-toxicity (approved by Food and Drug Administration, FDA, USA) [53,54], and a wide range of temperatures in which the Pluronic F-127 dosimeter can be stable. This resulted in the development of several compositions of 3D dosimeters with Pluronic F-127 [13,14,15,23,24,29,31,36,44]. The optimal composition of the Fricke-Pluronic F-127 dosimeter was found to contain 1 mM ammonium iron (II) sulphate hexahydrate ((NH_4_)_2_Fe(SO_4_)_2_·6H_2_O, FAS), 50 mM sulfuric acid (H_2_SO_4_), and 0.165 mM xylenol orange (XO) in 25% Pluronic F-127 [14]. Such a dosimeter responds linearly to irradiation up to 25 Gy (maximum tested dose; dynamic response to a dose above 60 Gy), has a dose sensitivity of 0.1316 ± 0.0022 Gy^−1^ cm^−1^ for irradiation with 6 and 15 MV photons, and should be stored before irradiation in a refrigerator (stability up to 120 h and after irradiation approximately 24 h; after irradiation, it is best stored in a dark place at room temperature; readability within the first 2 h) [14]. According to the findings [13], it is possible to lower the sol–gel–sol transition temperatures of the Fricke-XO-Pluronic F-127 dosimeter. This can be achieved by adding 0.2 M sodium chloride to the dosimeter formula, which results in a reduction of the sol–gel transition by ~2 °C. In addition, the sensitivity of the dosimeter to the dose is improved, the linear and dynamic response to the dose remains the same as in the case of the dosimeter without NaCl, and the dosimeter reacts to photons (6–15 MV) in a similar way as the previous composition [14], but NaCl negatively affects its chemical stability during storage.

**Figure 1:**
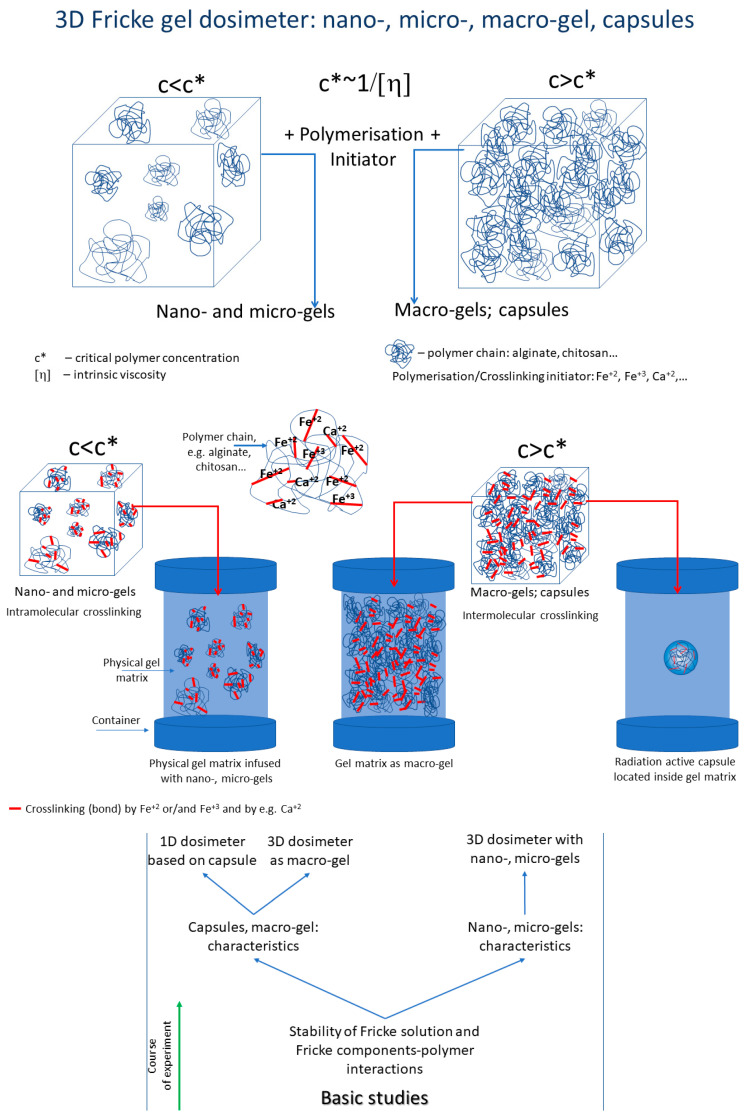
The concept of the Fricke 3D gel dosimeter. 3D gel matrix containing (i) nano- or micro-gels made of alginate (chitosan or other ionic polymer) crosslinked with Fe ions (also with, e.g., Ca ions), (ii) a macro-gel made of alginate (chitosan or other ionic polymer) crosslinked with Fe ions (also with, e.g., Ca ions), and (iii) a single capsule made of alginate (chitosan or other ionic polymer) crosslinked with Fe ions (also with, e.g., Ca ions). Below is a scheme illustrating the course of the study related to the manufacturing of the Fricke 3D radiotherapy gel dosimeter materials as capsules, macro-gels, and containing nano- or micro-gels in structure.

So far, the Fricke gel dosimeters have been produced mainly by dissolving the components of the Fricke dosimeter in a polymer matrix. The instability of most Fricke dosimeters regarding the integrity of the recorded 3D dose distribution is an obstacle during applications and requires fast 3D optical scanning methods to obtain reliable results. In turn, the current work examines new ways of manufacturing different dosimetry structures and investigates the possibility of enhancing the stability of the Fricke 3D dosimeter (Figure 1). The general idea is based on the reaction of metal ions (and therefore also Fe ions) with ionic polymers depending on the critical polymer concentration [55] (c*, reciprocal of intrinsic viscosity). Two different reaction conditions were chosen: (i) significantly below c* and (ii) above c*. The idea relates to our previous findings regarding the manufacturing of golden-, silver-, and silver-golden-chitosan physical hydrogels by a simple reaction of Au^+^, Ag^+^, or Au^+^ and Ag^+^ with a cationic polymer of chitosan [56,57]. The reaction of metal ions with ionic polymers with a concentration below c* can lead to the formation of nano- and micro-gels through intramolecular crosslinking. However, if the polymer concentration is above c*, macro-gels can be obtained as a result of intermolecular crosslinking, the so-called wall-to-wall, and thus also polymer capsules. These reactions were used to propose various polymer–metal structures for possible applications of ionizing radiation dosimetry.

The aim of this work was to present a concept for the preparation of Fricke gel dosimeters (Figure 1) and to propose alternative Fricke dosimetry materials based on nano-, micro-, and macro-gel structures. For this purpose, the following scope of research was carried out: (i) examination of the reaction between sodium alginate and the components of the Fricke dosimeter solution on the production of nano-, micro-, and macro-gels, (ii) characterization of each group of gels, (iii) study of the influence of Fe and Ca ions on the formation of alginate capsules, (iv) study of diffusion of the Fricke dosimeter components from alginate capsules, (v) test of radiation-sensitive macro-gel preparation, (vi) a proposal for a 1D radiotherapy dosimeter based on a radiation-sensitive Fe-polymer capsule embedded in a physical gel matrix, (vii) study of reducing the diffusion of Fricke ingredients from Fe-polymer capsules by preparing multilayer capsules using poly-L-lysine, and (viii) study of the manufacture of the Fricke dosimeter with Fe-polymer nano- and micro-gels embedded in a physical gel matrix. This work also seeks ways to reduce the diffusion of Fe ions in Fricke 3D gel radiotherapy dosimeters using the described concept. The workflow is illustrated in Figure 1, where initially the stability of the components of the Fricke solution and the interaction of the Fricke components with polymers were examined, and then nano-, micro-, and macro-gel structures were produced and characterized.

## 2. Results and Discussion

### 2.1. Stability of Fricke Solution and Interactions of Ingredients

The influence of water purity on the stability of iron ions was examined for 0.1–10 mM FAS solutions prepared in tap water, distilled water, re-distilled water, and deionized water stored at room temperature or in a refrigerator (Supplementary Figures S1–S8). The use of tap water results in immediate precipitation of sediment from the solution, regardless of the FAS concentration and storage conditions. For distilled water, precipitation occurs within 24 h of preparation. The amount of precipitate is smaller than for tap water, but precipitation occurs regardless of sample concentration. Samples stored in the refrigerator contained less sediment 24 h after preparation compared to samples stored in the cabinet at room temperature. The use of re-distilled and deionized water gave very similar results. In both cases, no precipitation was observed for the least concentrated sample throughout the entire experiment period. For a concentration of 0.5 mM, the precipitation occurs after approximately 48 h in very small amounts, and the situation is similar for samples with a concentration of 1 mM. In both cases, the method of storage has no apparent significance, but analyzing the absorption spectra (Supplementary Figures S9–S16), it can be concluded that changes occur slightly slower for samples stored in the refrigerator. In the case of deionized water, gas bubbles of unknown origin were observed in large quantities in all samples stored in the cabinet. The low pH may indicate dissolution of carbon dioxide, but re-distilled water has a comparable pH and gas bubbles were not observed. It is worth noting that the characteristics of deionized and re-distilled water are very similar (as is the stability of iron when using these solvents). The difference in iron stability between solutions with distilled and re-distilled water is clearly visible in this experiment, so it should propagate onto the stability of Fricke dosimeters. For this reason, all solutions and dosimetric systems examined further in this work were prepared with re-distilled water.

Preparation of nano-, micro-, and macro-gel structures according to the scope of this work required investigation of the stability of the component substrates in solutions, their chemical character, and possible interactions between some of them. Therefore, streaming current measurements were performed for FAS, XO, CaCl_2_, sodium alginate, iron(II) lactate, iron(II) gluconate dihydrate, sulfuric acid, gelatine, Pluronic F-127, and for the reacting components of sodium alginate and FAS, sodium alginate and CaCl_2_, sodium alginate and iron(II) lactate, sodium alginate and iron(II) gluconate dihydrate, and FAS and XO. The solutions were prepared in double-distilled water, for which the streaming current is −490 mV. The results are presented in Supplementary Figure S17. The streaming current for the compounds is related to their concentration (Supplementary Figure S17A–I). It increases with an increase in concentration for FAS, CaCl_2_, iron(II) lactate, iron(II) gluconate dihydrate, gelatine, and Pluronic F-127. There is a reverse tendency for sodium alginate; it decreases if the concentration of sodium alginate increases. For XO, the streaming current vs. concentrations changes in a more complex manner. First, it decreases for the two lowest concentrations, which is followed by a significant increase for 0.1 mM concertation and then a drop in values for the remining concentration, which oscillate around similar values of streaming current. The streaming current for sulfuric acid was challenging to record properly, most probably due to the high conductivity of this solution. The streaming current for gelatine takes positive values over the entire concentration range (Supplementary Figure S17G). This may have some consequences for the interactions between alginate micro-gels containing Fe ions and the gelatine matrix of a dosimeter composed of micro-gels and gelatine matrix. The streaming current for Pluronic F-127, which is a non-ionic co-polymer, increases with its concentration (Supplementary Figure S17H). The reason for this, as can be speculated, may be Pluronic F-127 molecules adsorption at the interface (gold electrodes–solution) and shielding against water molecules of a highly negative streaming current.

Each solution of the compounds characterizes with specific stability over the time of measurement, as shown in Supplementary Figure S17. The most stable are CaCl_2_ and iron(II) gluconate dihydrate over the entire range of concentrations examined and XO for all concentrations apart from 0.1 mM. For FAS and sodium alginate, an increase in streaming current was observed for all concentrations; the same applies to XO for 0.1 mM. For iron(II) lactate, the streaming current initially decreases then stabilizes, and at a longer measurement time, it can increase for some concentrations. The distinctive feature is that the streaming current is negative for all compounds; however, particularly for low concentrations of compounds, the negative streaming current of double-distilled water can contribute to the streaming current values of the solutions. Therefore, the observation of the streaming current values does not propagate on the conclusions on the possible reactions between these negatively charged compounds. Hence, it was important to investigate the reactions of Ca^+2^ and, most importantly, Fe^+2^ with sodium alginate to verify the assumption on the possibility of binding these ions with sodium alginate. Firm binding of Fe^+2^ with sodium alginate can be used for reducing the diffusion of iron ions in a 3D dosimeter matrix. The results are shown in Supplementary Figure S17J–L. From these results, it is evident that both Ca^+2^ and Fe^+2^ react with sodium alginate, and in consequence, the streaming current values increase, becoming less negative. The reaction between the components can also burden measurement, which is related to either precipitation of the product or formation of, e.g., micro-gels and even macro-gels. This is visible from the large fluctuation of the values and even gel formation in the measurement cell (Supplementary Figure S17K,L). The results in this section showed the interaction between sodium alginate, iron, and calcium ions, which were further used for the manufacturing of nano-, micro-, and macro-gels/capsules.

### 2.2. Features of Capsules

Alginate capsules were manufactured by dropwise addition of sodium alginate solutions from a syringe to forming solutions containing divalent metal ions. Reaction between divalent metal ions with alginate chains resulted in crosslinking of the chains and formation of capsules. According to the results of the preliminary experiments, the formation of alginate capsules is related to several factors, as follows: (i) viscosity and concentration of alginates, (ii) diameter of needle outlet for alginate solution, (iii) distance between the syringe outlet and the surface of forming solution, (iv) concentration of forming solution, and (v) alginate flow rate. Changes in these parameters results in different sizes and shapes of the capsules. For instance, a too-high flow rate of alginate solution from the syringe allows the formation of alginate polymer rods instead of beads. To obtain droplets, and then beads, the flow rate was reduced to an arbitrary chosen value. In turn, the capsules will not form if the alginate solution is added dropwise to pure water. Also, a very low concentration of forming solution will not result in the formation of mechanically firm capsules. Thus, it was set to be 1% or higher. Additionally, the diameter of the needle affixed to the syringe or syringe outlet is related to the size of the capsules. In this work, the alginate capsules (beads) were produced for one size of syringe outlet to obtain capsules a few millimeters in diameter. The distance between the syringe outlet and the surface of the forming solution was set to approximately 25 cm so that each drop is submerged under the surface of the forming solution. The concentration and viscosity of alginates play a major role in the formation of alginate capsules. Therefore, three alginates of different viscosities were tested in this work and their concentrations were chosen after preliminary experiments to be 1.7% and higher.

The capsules made by dropping the sodium alginate solution of different concentrations (1.7, 2, 2.3, 2.7, 3, and 3.5%) into the Fricke solution containing 0, 1, 1.5, 2.5, and 3.5% CaCl_2_ are presented in photographs in Supplementary Figures S18−S30 and Figure 2, Figure 3 and Figure 4 for Sigma low, Sigma medium, and Heppe medium viscosity, respectively. A criterion for organoleptic evaluation of the capsules was chosen, that the proper capsules should have a repetitive, spherical shape and sufficient mechanical strength so that they do not disintegrate when gripped with the fingers.

The summary of chosen conditions for manufacturing the best alginate capsules accepting the criterion is presented in Table 1. In brief, in the case of the low-viscosity Sigma alginate, acceptable capsules (mostly spherical and of adequate mechanical strength) or very good capsules (all spherical and of adequate mechanical strength) are formed for 1.7–3.5% alginate solution in reaction with the Fricke solution and 1.5–3.5% CaCl_2_. However, for medium-viscosity Sigma alginate, the only acceptable capsules were formed from 1.7% sodium alginate in reaction with the Fricke solution and 1.5% CaCl_2_. For other reaction conditions it was impossible to obtain well-shaped capsules. When using lower concentrations of alginate, discs of different thickness were obtained, while at higher concentrations, a teardrop shape was obtained. This is due to the high viscosity of the alginate. At high concentrations, the droplets coming out of the syringe are unable to relax into a spherical shape on the way from the syringe outlet to the surface of the liquid. For Heppe alginate, acceptable or very good capsules can be formed for 1.7–3.5% alginate solution, the Fricke solution, and 1–3.5% CaCl_2_. Heppe alginate capsules appear clearer than those prepared with Sigma alginates (no filtering of alginate solutions was applied). The mechanical properties of the capsules assessed organoleptically appear to be comparable to those of low-viscosity Sigma alginate capsules. Based on the above observations, low-viscosity Sigma alginate and medium-viscosity Heppe alginate are the most promising for capsule formation for dosimetric purposes.

In addition, a very important observation was made for the capsules prepared without Ca^+2^ in the forming solution for all alginate concentrations and alginate types (viscosities) (Supplementary Figure S18). The capsules are of irregular shape; however, the fact they are formed confirms that Fe^+2^ ions bind sodium alginate chains, which leads to physical hydrogels.

**Figure 5:**
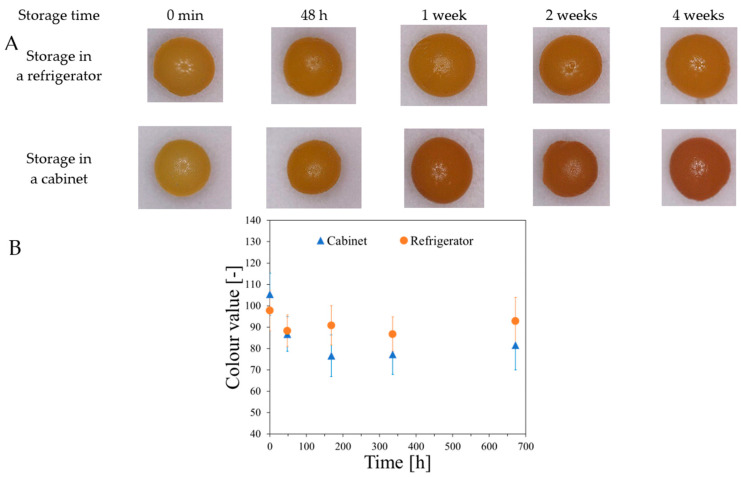
Stability of the capsules made in a reaction of 3.5% alginate (low viscosity Sigma) with Fricke solution (FAS: 1 mM, XO: 0.165 mM, H_2_SO_4_: 50 mM) containing 3.5% CaCl_2_ by dropwise addition of sodium alginate solution into the Fricke solution with Ca^+2^ (**A**). The capsules stored in the Fricke solution either in a refrigerator at approximately 4 °C or in a cabinet at approximately 21–23 °C (without access to daylight). Top of the figure contains photographs of the capsules during storage (**A**). The graph (**B**) corresponds to the color values of the capsules read with ImageJ software (v. 1.53j, National Institutes of Health, Bethesda, MD, USA) over time of storage (mean values of circular ROIs covering the entire capsules).

**Figure 6:**
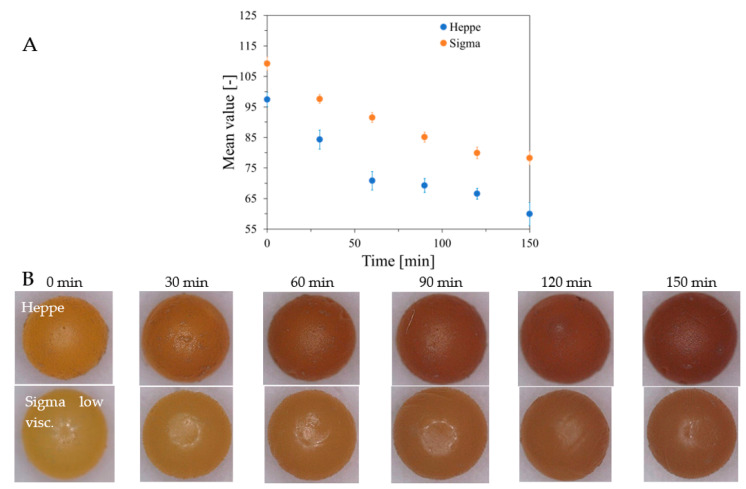
(**A**): Stability of alginate capsules containing Fricke solution. A mean value of color for the photographs taken with a microscope (Delta Optical Smart 5MP PRO digital microscope, Delta Optical, Nowe Osiny, Poland) versus storage time at approximately 25 °C with access to laboratory light, removed from Fricke solution, for the capsules formed with Heppe medium-viscosity and Sigma low-viscosity alginates. Beneath the graph are the photographs (**B**) of the capsules taken at 0–150 min after formation (first row: capsules made of Heppe and second row: capsules made of Sigma alginate). The capsules were made in a reaction of 3.5% alginate with Fricke solution (FAS: 1 mM, XO: 0.165 mM, H_2_SO_4_: 50 mM) containing 3.5% CaCl_2_ by dropwise addition of sodium alginate solution into the Fricke solution with Ca^+2^.

**Figure 7:**
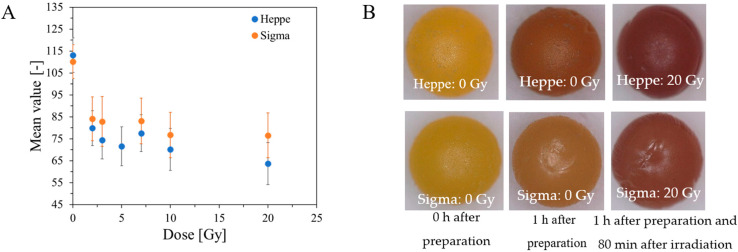
Response of alginate capsules containing Fricke solution to irradiation with X-rays of a TrueBeam medical accelerator (Varian, Palo Alto, CA, USA): mean value of color for the photographs taken with a microscope (Delta Optical Smart 5MP PRO digital microscope) versus absorbed dose (**A**) and example photographs of non-irradiated and irradiated capsules (**B**). The capsules were made of Heppe and a low-viscosity Sigma alginate in a reaction of 3.5% alginate with Fricke solution (FAS: 1 mM, XO: 0.165 mM, H_2_SO_4_: 50 mM) containing 3.5% CaCl_2_ by dropwise addition of sodium alginate solution into the Fricke solution with Ca^+2^. The capsules were immersed (0 Gy) and removed from the Fricke solution (20 Gy) and not immersed in the solution after irradiation. The photographs of capsules are magnified: ×38. The values in A are averages for 3–6 capsules and were calculated from approximately 50,000 pixels.

The stability of alginate capsules produced by dropwise addition of sodium alginate solution to Fricke’s solution containing Ca and XO ions was tested for capsules stored in Fricke’s solution in a refrigerator (at approximately 4 °C) or cabinet (approximately 21–23 °C) with access to daylight. The results are shown in Figure 5. It was found that the best storage condition for the capsules was low temperature (refrigerator), which preserves the color for several weeks. When alginate capsules are removed from the forming solution (Fricke’s solution with or without calcium ions) and stored at approximately 25 °C with access to laboratory light, their color is unstable and changes over time, as shown in Figure 6. The color of Heppe and Sigma alginate capsules changes from light yellow to brown. The color change is analogous to that when the capsules are irradiated with ionizing radiation (Figure 7). When capsules are irradiated, their color changes after absorbing the dose and does not change during storage in air, unlike non-irradiated capsules. Capsules made of Heppe alginate appear to be slightly more sensitive to irradiation than those made of Sigma alginate, as the color changes to a darker brown.

### 2.3. Analysis of Fe, Ca and XO in Alginate Capsules

**Figure 8:**
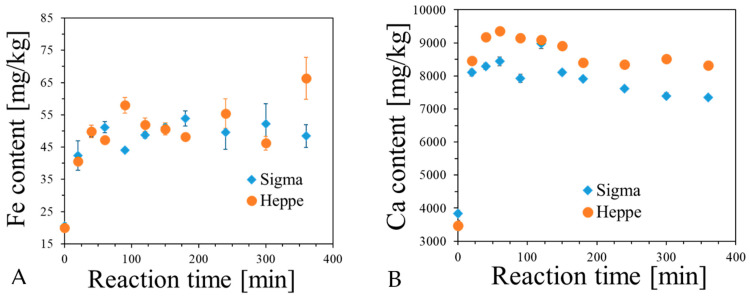
Content of Fe (**A**) and Ca (**B**) per 1 kg of capsules (average weight of one capsule: 0.034 g) in relation to reaction time between sodium alginate solution (Sigma and Heppe) and Fricke solution (FAS: 1 mM, H_2_SO_4_: 50 mM, XO: 0.165 mM) containing 3.5% CaCl_2_. Measurements were performed with the aid of ICP-OES. The reaction was carried out at a temperature of approximately 23 °C. Throughout the experiment, the contents of the beaker were stirred with a magnetic stirrer at a speed of approximately 450 rpm.

**Figure 9:**
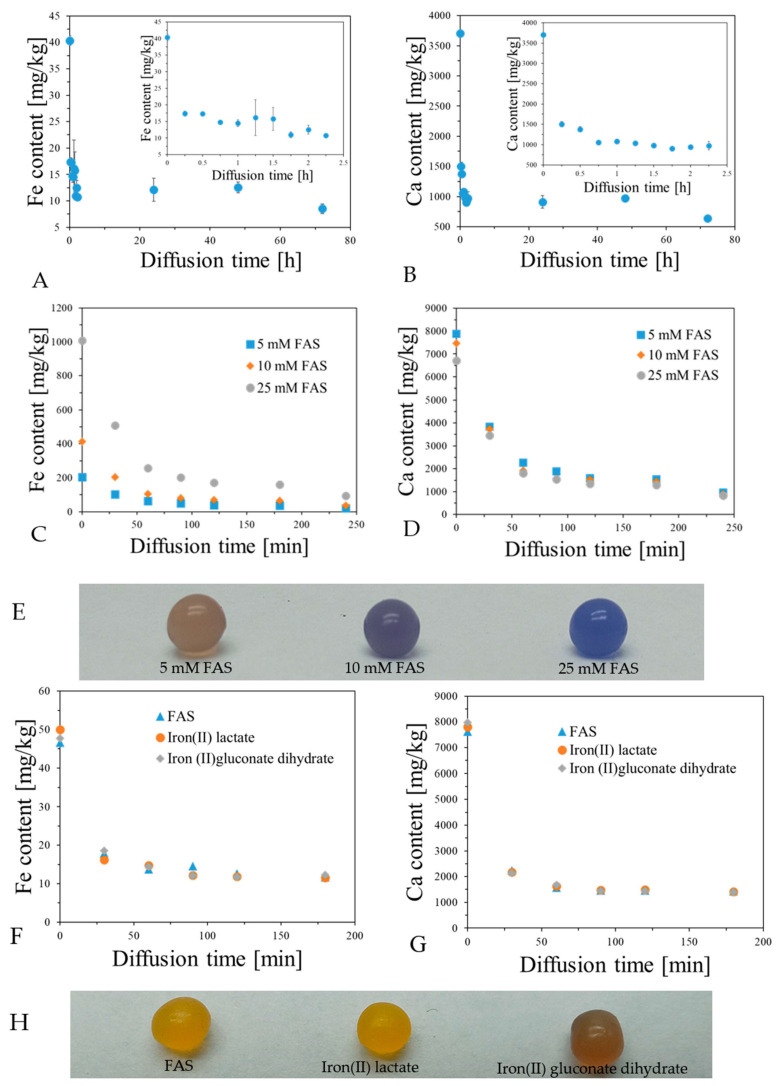
Examination of diffusion of Fe (**A**,**C**,**F**) and Ca (**B**,**D**,**G**) from alginate capsules (average weight of one capsule: 0.034 g) made by reaction of sodium alginate solution (Sigma) (3.5%) with Fricke solution (FAS: 1 mM, H_2_SO_4_: 50 mM, XO: 0.165 mM; reaction time = 40 min) containing 3.5 CaCl_2_ (**A**,**B**), different concentrations of FAS (**C**,**D**), and different compounds containing Fe (**F**,**G**) (concentration of each compound: 1 mM). In (**E**,**H**), photographs of capsules made of different concentrations of FAS and different compounds containing Fe, respectively, are shown. The capsules were immersed in 3.5 mL of re-distilled water. Measurements were performed with the aid of ICP-OES. For (**A**,**B**), the water with the capsules was changed after 48 h of diffusion, for (**C**,**D**), the water was changed after 180 min, and for (**F**,**G**), after 120 min.

It was proven in previous sections that alginate capsules can be formed by reaction of sodium alginate solution with Fe ions, with and without accompanying Ca ions. The formation of such capsules occurs by adding sodium alginate solution into aqueous solution of such ions and keeping them beneath the surface of the solution for an arbitrary chosen time. The capsules are formed by binding the ions to carboxylate groups of alginates, which leads to intermolecular crosslinking. However, it was supposed that apart from such reaction, the capsules may also contain non-bonded ions by simply soaking up the Fricke solution with or without calcium ions. For this reason, it was necessary to investigate the content of Fe and Ca ions in capsules using ICP-OES for the following cases: (i) in relation to reaction time between sodium alginate solution and Fricke solution containing 3.5% CaCl_2_ (Figure 8); (ii) for the capsules prepared by reaction of sodium alginate with Fricke solution containing 3.5% CaCl_2_ and different concentration of FAS: the diffusion of Fe and Ca ions from capsules was studied (Figure 9); (iii) for the same case as (ii), but instead of FAS, other compounds containing Fe ions were tested: iron(II) lactate and iron(II) gluconate dihydrate (Figure 9).

The content of Fe and Ca in alginate capsules increases with reaction time (Figure 8). For Fe, it stabilizes at approximately 100 min of reaction, while for Ca it reaches a maximum value at approximately 50 min and then the Ca content decreases; at approximately 300 min of reaction, it seems to stabilize. Comparing two types of alginates, they bind Fe ions similarly, but Heppe has a higher affinity to bind Ca ions than Sigma alginate. In turn, the diffusion experiments (Figure 9) revealed that both Ca and Fe are not retained in capsules when they are immersed in water. Most ions transfer to water. For instance, only approximately 25% of Fe ions and 25% of Ca ions remain in alginate capsules (Figure 9A,B). The concentration of FAS in Fricke solution does not impact on Fe content in capsules during the diffusion studies, however, the higher this concentration is, the slightly more Fe is retained in the capsules (Figure 9C,D). In turn, the higher the concentration of FAS in Fricke solution, the more the capsules convert to a blue color (Figure 9E). This prevents them from being used as 1D dosimeters. In addition, there is no impact of the type of compound containing Fe ions on their retention in the capsules immersed in water (Figure 9F,G). The type of compound containing Fe ions influences the initial color of alginate capsules; it is similar for FAS and iron(II) lactate but much darker yellow for iron(II) gluconate dihydrate (Figure 9H). Apart from examination of Fe and Ca diffusion form alginate capsules, XO diffusion was also assessed (Supplementary Figure S31). By these results, it is clear that XO is not retained permanently in the capsules in large quantities, and approximately 95% is released to water. To sum up, although the alginate capsules can be formed by crosslinking alginate chains by both Fe and Ca ions by adding the alginate solution dropwise into the Fricke solution containing XO and Ca ions, most of the components that are important from the dosimetric point of view are released when the capsules are immersed in water. The remining bonded components play an important role during irradiation of the capsules, as discussed below.

### 2.4. Macro-Gel and Characteristics of Nano- and Micro-Gels

A view of macro-gel prepared by adding 1 mM FAS and 50 mM of sulfuric acid to 1.35–1.75% sodium alginate is presented in Supplementary Figure S32. The view of macro-gels containing additional 0.165 mM XO, before and after irradiation with a dose of 30 Gy are presented in Supplementary Figure S33. The obtained gels change color after irradiation; however, due to their inhomogeneity, reading the dose distribution using optical methods is impossible. It is necessary to optimize the composition and the method of preparation to obtain a homogeneous macro-gel, which is not part of this work.

The first composition tested for obtaining nano- and micro-gels by reaction of sodium alginate with Fricke solution components consisted of 0.015–0.65% Sigma low-viscosity sodium alginate, 1 mM FAS, and 50 mM H_2_SO_4_. Additionally, a sample containing 0.5% sodium alginate and 50 mM H_2_SO_4_ without FAS was prepared. The pH value of the tested solutions ranged from approximately 1.4 (for the lowest alginate concentrations) to approximately 1.8 (for the highest alginate concentrations). The obtained gels are presented in Supplementary Figures S34 and S35 and the transmittance spectra for the nano- and micro-gel solution are presented in Supplementary Figure S35D. The graph shows (Supplementary Figure S35D) that as the concentration of alginate decreases, the transmittance value of the samples increases. For the lowest concentration of 0.015%, the transmittance is similar to the transmittance of re-distilled water. In the photographs in Supplementary Figure S34, suspended white structures are visible in the solutions, the number of which decreases with decreasing alginate concentration. The same structures are also visible in the solution without FAS (Supplementary Figure S34H), which indicates that the precipitated compound is not iron alginate but most likely alginic acid. In an environment with a pH value below approximately 3.6 [58,59], the carboxyl groups of alginate protonate, and then hydrogen bonds are formed between the hydrogens of the carboxyl groups, which leads to the formation and precipitation of a physical gel. To avoid the formation of alginic acid, the pH of the solutions should be increased by reducing the concentration of sulfuric acid. It should be noted, however, that the addition of acid not only increases the efficiency of the radiation oxidation of iron ions [60] but also inhibits the oxidation of ferrous ions by oxygen present in the solution [61]. When 0.1 mM XO was added to a solution of 0.5% alginate and 1 mM FAS, without sulfuric acid, the initial orange color of the solution changed to purple (indicating oxidation of the iron present in the solution) within approximately 3 min (Supplementary Figure S35E). However, as can be seen in Supplementary Figures S6 and S7, the oxidized iron compounds did not precipitate from the FAS solution in re-distilled water (pH = 5.77) until 24 h after preparation. According to the literature, the presence of Fe^+3^ complexing anions in the solution significantly increases the oxidation rate of Fe^+2^ ions [62]. Xylenol orange added to Fricke solutions is a complexing agent for ferric ions; hence, the decision was made not to add the dye to the dosimeters containing nano- and micro-gels. It should be noted that removing the dye makes it impossible to read the dose distribution using optical methods (current 3D optical computed tomography scanners), although it should still be possible to read using nuclear magnetic resonance (1D) and magnetic resonance imaging (3D).

For the next attempt to obtain nano- and micro-gels, 0.1–0.6% sodium alginate Heppe, 1 mM FAS, and 1–3 mM sulfuric acid were used. The concentrations were selected so that the pH of the solutions was in the range of 3.9–4.2 to significantly reduce the degree of protonation of the carboxyl groups of alginate while increasing the rate of autoxidation of Fe^+2^ ions as little as possible. After preparing the solutions, their transmittance was measured (Supplementary Figure S36A), and in addition, within 360 h of preparation, measurements of the hydrodynamic diameters of the micro-gels were carried out using DLS (Supplementary Figure S36B). Cuvettes with FAS and alginate solutions are presented in Supplementary Figure S36C–E. The transmittance of all solutions increased significantly compared to samples containing 50 mM sulfuric acid (Supplementary Figure S35D), and no precipitated compounds were observed with the naked eye in fresh solutions (Supplementary Figure S36C). The influence of alginate concentration and storage time on the diameters of structures present in solutions cannot be determined based on DLS measurements. The obtained results are arranged in a chaotic manner, which may be due to the presence of air bubbles formed while vigorously mixing the solution during addition of FAS and sulfuric acid to alginate. The microscopic photograph of the sample containing 0.5% alginate shows many structures suspended in the solution (Supplementary Figure S36F). The yellow color of the visible structures suggests the presence of iron bound to alginate, but further studies (e.g., analysis using a transmission electron microscope (TEM)) would be necessary to confirm this. Nevertheless, the mean hydrodynamic diameter of the structures formed is not higher than 140 nm.

The effect of radiation on the formation of nano- and micro-gels was studied by irradiating solutions of 0.5% sodium alginate (both medium-viscosity Heppe and low-viscosity Sigma were tested) and 2 mM FAS. Two variants of solutions were prepared, differing in the content of sulfuric acid. In one variant, 2 mM H_2_SO_4_ was added (pH of solutions approximately 3.9), while in the other, no acid was added (pH approximately 5.7). Approximately 1 h passed from the preparation of solutions to irradiation, and the assessment of the formed structures was carried out by taking microscopic photographs one hour after irradiation. The obtained images are presented in Supplementary Figures S37–S40. All samples contained suspended structures similar to those observed in Supplementary Figure S36F. This may indicate rapid oxidation of Fe^+2^ ions and the formation of iron alginate structures. It should be noted that sodium alginate contains up to approximately 40% of ash, which is an inorganic salt found in algae from which the polymer [63] was produced. Among the salts, there may be copper compounds [64], which, even in trace amounts, are a catalyst for the oxidation of Fe^+2^ ions [62], as well as other metals that can bind with alginate, which can lead to the formation of microstructures. Further studies are necessary to better understand all the processes occurring in the tested solutions. However, taking into account the multitude of complications related to the use of alginate in the Fricke dosimeter, such as the mutually exclusive optimal pH conditions for sodium alginate and the Fricke solution, the effect of ash contained in alginates on the rate of autooxidation of Fe^+2^ ions, and the need to remove the dye from the dosimeter composition, investigation of other polymers capable of binding ferric ions is suggested. A modification of the capsules by incorporation of other Fe ion-binding polymers in their structure would be an option as well.

The effect of the presence of a micro-gel on the response of the Fricke dosimeter to ionizing radiation was checked by irradiating (0–20 Gy) a composition containing 0.015% sodium alginate, 1 mM FAS, 50 mM sulfuric acid, and 0.165 mM XO. The photographs of irradiated samples (Supplementary Figure S35F) show that the irradiation is accompanied by a dose-dependent color change. A characteristic feature of some samples is the appearance of macrostructures (aggregates) with a clearly darker color than the rest of the sample. The number of structures is the greatest for the highest doses. No such observations were seen immediately after irradiation. It is possible that the gels turned into a sol during transport, which facilitated aggregation. Due to the formation of alginic acid and the presence of competing XO, only a small part of the Fe^+3^ ions is predicted to be bound to the alginate.

### 2.5. 1D Capsule Dosimeter

**Figure 10:**
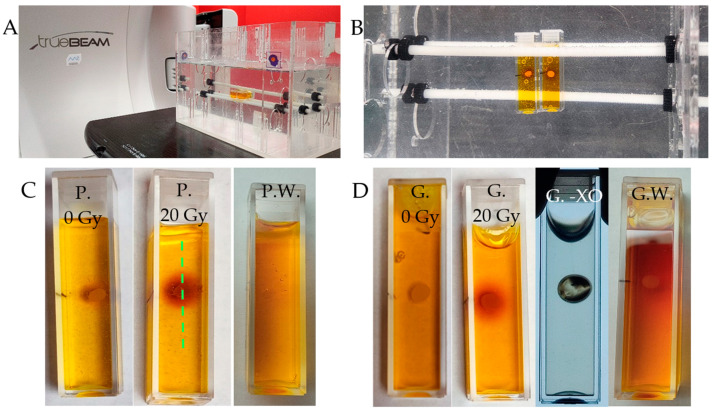

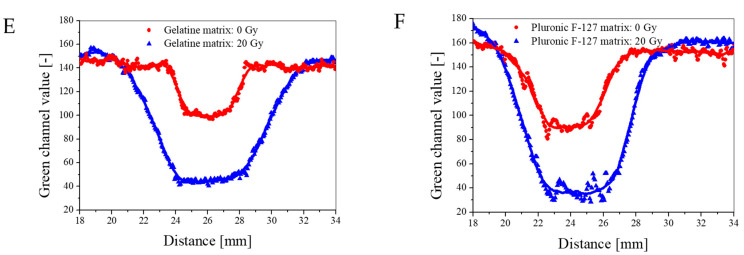
Response to irradiation of alginate capsules with Fricke solution in Pluronic F-127 or gelatine matrix. (**A**): Irradiation set up. Samples in cuvettes of 4.7 cm height and 1 cm optical path are immersed in water (water phantom, W-1-DD1-2, GeVero Co.); the water phantom is on the bench of the TrueBeam accelerator (Varian). (**B**): Top view of the samples in the water phantom. (**C**,**D**): Samples before and after irradiation (20 Gy) for the dosimeters with Pluronic F-127 (**C**) and gelatine (**D**) matrix; in (**D**), a cuvette with gelatine matrix and capsule containing Fricke dosimeter without XO dye is also shown (G.-XO) for comparison. P. and G. denote Pluronic F-127 and gelatine, respectively; P.W. and G.W. denote Pluronic F-127 and gelatine matrices, respectively, with capsules that were washed (for 2 days) before immersion in the matrices and after irradiation (20 Gy). (**E**,**F**): Profiles along the samples as indicated by green dashed line in (**C**). The values are for the green channel of RGB color space.

The use of alginate capsules containing a Fricke dosimeter solution was tested in a radiotherapy environment (Figure 10A,B), where a linear accelerator (X-rays) was used for irradiation. The experiment aimed to test such a system for point measurements of radiation dose. Fricke-alginate capsules were embedded in physical gel matrices of Pluronic F-127 or gelatine. The experimental results prove the response of the Fricke-alginate capsule in the gel matrices to X-ray irradiation, which was manifested by a change in the color of the capsules. Due to the diffusion of Fe^+2^ from the capsules, their surroundings also change color during irradiation (Figure 10C,D). The cuvettes with capsules in the gel matrices were photographed, and the profiles passing through the capsules and their surroundings were analyzed (Figure 10E,F). The results clearly show a decrease in the green channel value (RGB color model) after 20 Gy absorption, which is similar for both matrices. In this case, this system consisting of cuvettes with capsules containing a Fricke dosimeter embedded in a gel matrix has shown promise for dosimetry applications. The experiment also revealed a prerequisite for the successful functioning of the capsules, in that the release of the Fricke solution from the capsules worsens their dose response and color change; too low a content of the Fricke solution in the capsules, obtained by washing the capsules, eliminates their use as dosimetry systems. In conclusion, the alginate capsules of the present development should be considered as reservoirs of the Fricke solution, but they require further chemical modifications to prevent the leakage of the Fricke components. In addition, the effect of the matrix on the shape of the capsules was observed, which is discussed below.

**Figure 11:**
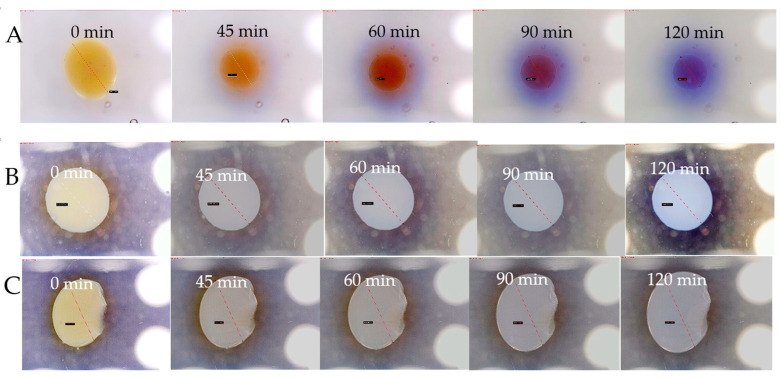

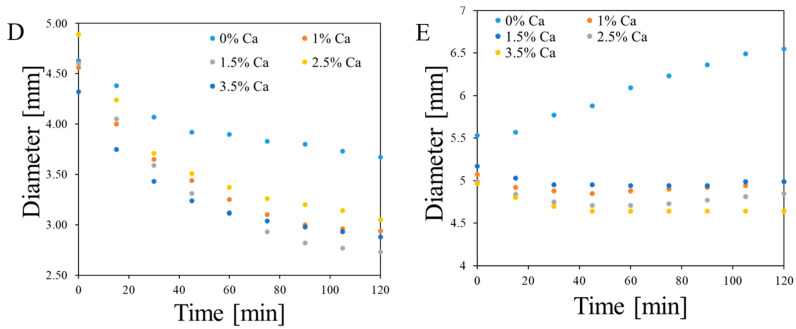
Performance of alginate capsules containing Fricke dosimeter embedded in Pluronic F-127 and gelatine matrices. (**A**): Pluronic F-127 matrix, c CaCl_2_ = 1.5%; (**B**): gelatine matrix, c CaCl_2_ = 1.5%; (**C**): gelatine matrix, c CaCl_2_ = 0%; (**D**,**E**): change of size of alginate capsule in Pluronic F-127 (**D**) and gelatine (**E**) matrix over time of storage at room temperature (~23 °C). Capsules formed in a reaction of 3.5% sodium alginate with Fricke solution: 1 mM FAS, 50 mM H_2_SO_4_, and 0.165 mM XO containing 0–3.5% CaCl_2_. Black bars in (**A**–**C**) denote the distance of 0.75 mm. Dashed lines indicate the positions where the diameter was measured.

**Figure 12:**
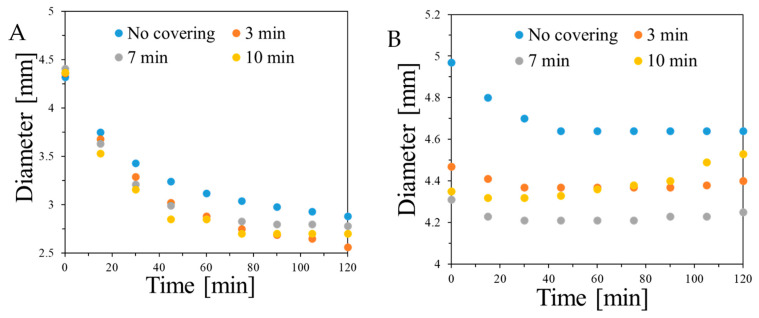

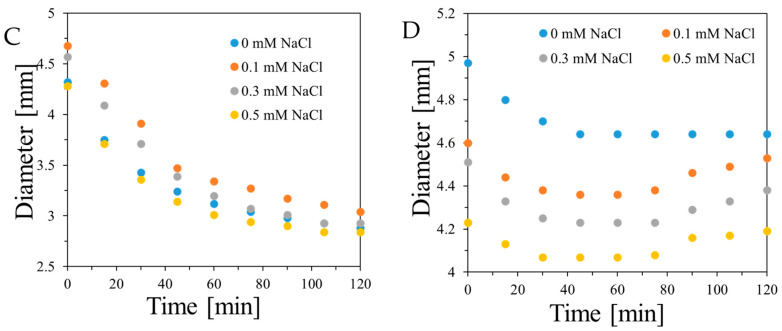
Performance of alginate capsules containing Fricke dosimeter embedded in Pluronic F-127 and gelatine matrices. (**A**,**B**) correspond to the capsules treated with poly-L-lysine and immersed in the Pluronic F-127 and gelatine matrices, respectively. (**C**,**D**) are for the capsules immersed in Pluronic F-127 (**C**) and gelatine matrices (**D**) at different ionic strength—concentrations of NaCl. Capsules formed in a reaction of 3.5% sodium alginate with Fricke solution: 1 mM FAS, 50 mM H_2_SO_4_, and 0.165 mM XO containing 3.5% CaCl_2_.

Figure 11 and Supplementary Figures S41 and S42 illustrate the performance of alginate capsules containing Fricke dosimetric solution, which are embedded in either Pluronic F-127 or gelatine matrix up to 120 min, for the capsules prepared by dropping sodium alginate solution into Fricke solution containing 0–3.5% CaCl_2_. In the cases of the alginate capsules in Pluronic F-127 (Supplementary Figure S41), three phenomena can be observed: (i) the capsules shrink over time, (ii) they release the components of the Fricke dosimeter, and (iii) the components released and the shrunk capsules change their color, which is related to the conversion of Fe^+2^ into Fe^+3^ associated with a complex formation with XO. The shrinking process occurs dynamically just after immersion of the capsules in Pluronic F-127 matrix and seems to stabilize after 120 min. The capsules without CaCl_2_ in composition are the least vulnerable to shrinking and those with 2.5% CaCl_2_ are the most vulnerable. The behavior of the alginate capsules is very different when they are embedded in gelatine matrix (Supplementary Figure S42). All the capsules containing CaCl_2_ shrink within only the first 40 min to a much lesser extent than the capsules embedded in Pluronic F-127, stabilize up to approximately 60 min, and swell and stabilize between approximately 110–120 min. An exceptional behavior was observed for the capsule without CaCl_2_ (Supplementary Figure S42E); they swelled significantly over the entire measurement time. The behaviours of the capsules in two different matrices is most likely associated with the type of polymer forming the matrices: non-ionic Pluronic F-127 and cationic gelatine, interactions between the components of the capsules and the matrices, and the ionic strength of the matrices and the capsules (Figure 12). The latter was taken into consideration. In this respect, the capsules were introduced into a Pluronic F-127 or gelatine gel matrix to which 0 to 0.5 mM NaCl was added. Increasing the ionic strength of the Pluronic matrix had no effect on the capsule shrinkage process, regardless of the concentration of added sodium chloride (Figure 12C). The capsules immersed in the gelatine matrix with the addition of NaCl shrank for the first 40 min after introduction into the gel (Figure 12D). After this time, they reached their minimum diameter, and after another 20 min, they began to increase their diameter. This is a different behavior than the capsules embedded in gelatine without salt, which also shrank for the first 40 min but, after reaching the minimum diameter, did not change further. The observations made indicate that increasing the ionic strength of the matrix did not overcome the phenomenon of changes in the capsule diameter during storage in the gel. An alternative attempt to stop the shrinkage of the capsules was to create a shell around them. For this purpose, the capsules were immersed in a 0.1% solution of poly-L-lysine for 0 to 10 min. Then, the capsules were introduced into a gel matrix. Regardless of the time of immersion in poly-L-lysine, covering the capsules did not stop their shrinkage in the gel matrix (Figure 12A,B). In this respect, and considering the diffusion of Fricke components for the capsules discussed above, other ionic polymers should be tested in the future to improve the performance of the capsules. For instance, introduction of poly(vinyl alcohol) (PVA) and polydimethylsiloxane may be one such possibility, as presented elsewhere [65]. In this work, Zhang et al. developed capsules (pellets) made of PVA with Fricke’s solution, which were additionally coated with polydimethylsiloxane (Sylgard 184). The obtained capsules with an average diameter of 2.57 mm were characterized by good mechanical strength. Moreover, after placing them in a gel matrix (PVA), no changes in the size of pellets or diffusion of dosimeter components outside the capsules were observed. In turn, however, a significant disadvantage of the proposed solution is the complex process of obtaining capsules, which consists of dripping the PVA solution from Fricke into liquid nitrogen (−196 °C), repeating the process of freezing (−20 °C) and thawing (25 °C) of the pellets three times, and then covering them with Sylgard by immersing them in degassed polydimethylsiloxane heated to 50 °C in a semi-cured state.

## 3. Materials and Methods

### 3.1. Type of Water

Water from four different sources was used in the experiment. The impact of different types of water on the stability of Fricke dosimeter components was examined. The types were tap water, distilled, re-distilled, and deionized. Each water was characterized for hardness, pH, and conductivity. Conductivity was measured with a CC-505 conductivity meter (ELMETRON, Zabrze, Poland) and pH was measured with a SevenExcellence S400 pH meter (Mettler Toledo, Warsaw, Poland). Water hardness was determined by titration of water samples with an EDTA solution at a concentration of 0.011 mol/dm^3^ (the titer was set to a standard CaCl_2_ solution at a concentration of 0.015 mol/dm^3^) in the presence of a mixture of eriochrome black and NaCl. For deionized and re-distilled water, the water hardness was below the mark limit. The types and characteristics of water are shown in Table 2. The stability of FAS aqueous solutions in particular types of water was checked by preparing solutions of various concentrations (0.1–10 mM). Changes occurring in the samples were assessed organoleptically (photographs with a digital camera) and through UV–Vis spectrophotometric measurements (Section 3.9).

### 3.2. Preparation of Capsules

Alginates with viscosity 2.800 mm^2^/s at 25 °C (0.1%) (low viscosity, Sigma-Aldrich, Saint Louis, MO, USA), 6.271 mm^2^/s at 25 °C (0.1%) (medium viscosity, Sigma-Aldrich, Saint Louis, MO, USA), and 5.366 mm^2^/s at 25 °C (0.1%) (medium viscosity, Heppe, Halle, Germany) were used in this work. The alginate solutions in re-distilled water (1.7–3.5%) were filled into 10 mL syringes (Braun, Melsungen, Germany), and then ~4 mL was added dropwise to the Fricke solution (50 mL) without or with calcium chloride (Chempur, Piekary Śląskie, Poland) to form capsules from the 4 mL alginate volume. Fricke solution consisted of 1 mM ammonium iron(II) sulfate hexahydrate (pure p.a., Chempur, Poland), 50 mM sulfuric acid (Chempur, Piekary Śląskie, Poland), and 0.165 mM xylenol orange (Sigma-Aldrich, Staint Louis, MO, USA). The concentration of calcium chloride ranged from 0 to 3.5%. Sodium alginate was added dropwise from a syringe without a needle attached. The outlet of the syringe (outside diameter: ~3.9 mm, inside diameter: ~2.3 mm) was approximately 25 cm above the surface of the Fricke solution. During the addition, the solution was gently stirred with a magnetic stirrer (approximately 150 rpm, IKA, Staufen im Breisgau, Germany); after the addition was complete, the rotational speed was increased to approximately 350 rpm. The capsules were kept in the continuously stirred Fricke solution for 15–360 min to increase the iron content of the capsules. During the research, the storage time in the solution was optimized.

### 3.3. Preparation of Macro-Gel

Macro-gels were obtained using a low-viscosity alginate (Sigma-Aldrich, Saint Louis, MO, USA) and a medium-viscosity alginate (Heppe, Halle, Germany). To 40 mL of solutions of sodium alginate in re-distilled water (concentrations in 50 mL of the solution were 2–3.5%), 10 mL of Fricke’s solution was added drop by drop. The concentrations of the components in 50 mL of the solution were as follows: 1 mM FAS, 50 mM sulfuric acid (VI), and 0.165 mM XO. The solution was stirred with a magnetic stirrer (C-MAG HS 7-IKA, Staufen im Breisgau, Germany) at a speed of approximately 800 rpm.

### 3.4. Preparation of Nano- and Micro-Gels

Low-viscosity alginate (Sigma-Aldrich, Saint Louis, MO, USA) and a medium-viscosity alginate (Heppe, Halle, Germany) were used to prepare the nano- and micro-gels. First, a sodium alginate solution was prepared in 80% of the final volume of redistilled water. In the remaining 20% of the water volume, FAS (Chempur, Piekary Śląskie, Poland) was dissolved, and then sulfuric acid (VI) was added. The solution prepared in this way was added in small portions (approx. 200 µL) to sodium alginate with vigorous stirring (approximately 800 rpm) (C-MAG HS 7-IKA, Staufen im Breisgau, Germany) to ensure good dispersion of iron ions and to prevent the formation of alginate and Fe^+2^ aggregates. The sulfuric acid and FAS solution was added using an automatic pipette, placing its tip in the alginate solution, close to the dipole. The final concentrations of the components were as follows: 0.015–0.65% sodium alginate, 0–2 mM FAS, 0–50 mM sulfuric acid (VI). The pH values of the micro-gels solutions were measured using pH-meter HI 221 (Hanna Instruments, Woonsocket, RI, USA). To characterize the obtained micro-gels with microscopic observations and DLS measurements, XO dye was not added to the solution.

### 3.5. Preparation of 1D Dosimeter

A gel matrix (Pluronic F-127 (Sigma-Aldrich, Saint Louis, MO, USA) or gelatine (Sigma, Saint Louis, MO, USA) was introduced into the PMMA cuvettes to a height of 2 cm of the cuvette. After the matrix was gelled, a capsule obtained according to 3.2 was placed in the center of the gel surface, and then the cuvette was filled with the gel matrix.

### 3.6. Preparation of 3D Dosimeter

XO was added to the micro-gel obtained according to 3.4. The concentration of the dye in the micro-gel solution was 0.165 mol/dm^3^. The gel matrix (Pluronic F-127 (Sigma-Aldrich, Saint Louis, MO, USA) or gelatine (Sigma-Aldrich, Saint Louis, MO, USA)) was dissolved in the micro-gel solution. The dissolution of Pluronic F-127 was carried out by pouring the solution on successive layers of Pluronic F-127 in the form of powder and then storing it in a refrigerator at a temperature of approximately 4 °C. Gelatine in the form of powder was dissolved in the micro-gel solution by heating the whole mixture to 50 °C while stirring at a speed of approximately 400 rpm with a magnetic stirrer.

### 3.7. Particle Charge Measurements

The measurements of the components of the Fricke dosimeter, sodium alginate, and calcium chloride were performed using the particle charge detector PCD 03 (Mutek, BTG Instruments, Weßling, Germany). Measurements were carried out for 10 min, taking reading every 30 s. Measurements of the reaction between components were carried out by gradually adding 50 µL of solutions of Fricke dosimeter components and calcium chloride to 15 mL of 0.25% sodium alginate.

### 3.8. Diffusion Measurements

The diffusion rate of iron and calcium ions from the capsules to water was tested by placing a sample of five capsules in a PMMA cuvette, which were then flooded with 3.5 mL of re-distilled water and closed with a polyethylene plug. The capsules were stored in water for a specified period (15 min–72 h). The capsules were then removed from the water and blotted for approximately 30 s on a paper towel and placed in glass tubes. The content of calcium and iron ions in the sample was determined using the ICP-OES technique (Section 3.11).

Measurements of the diffusion rate of the dye from the capsules to water were carried out by placing 15 capsules in a glass beaker with a volume of 25 mL and pouring them with 10 mL of re-distilled water. The capsules were stored for a specified period (20–180 min). Then, 3.5 mL of water was taken from the beaker and added into PMMA cuvettes. Spectrophotometric measurements (Section 3.9) were used to determine the concentration of the dye in the water.

### 3.9. UV–Vis Measurements

UV–Vis measurements were performed with a V-530 spectrophotometer (Jasco, Tokyo, Japan). The absorption and transmittance of the samples were measured in the range of 190–700 nm with a resolution of 1 nm. PMMA cuvettes were used for the measurements. Measurements were carried out in relation to air.

### 3.10. Dynamic Light Scattering Measurements

Distribution of the mean hydrodynamic diameter of structures formed by mixing sodium alginate solutions with components of Fricke dosimeter was assessed with the aid of dynamic light scattering (DLS, Particle Size Analyzer, NICOMP 380, Particle Sizing Systems Santa Barbara, CA, USA) at room temperature.

### 3.11. Assessment of Metal Content in Capsules

Before starting the measurements, the samples were weighed into glass tubes on an analytical balance with an accuracy of five decimal places. Approximately 0.2 g of sample was weighed, and 4 mL of concentrated nitric acid (65% HNO_3_, Baker Analyzed, Avantor, Inc. Radnor, PA, USA) was added. The tubes covered with Teflon caps were placed in the UltraWAVE microwave assisted digestion system (Milestone, Sorisole, Italy). In this system, inert gas (nitrogen) under pressure (40 bar) is pumped into the reactor before decomposition begins. The decomposition stages in microwave system consisted of three stages. Stage I: temperature inside the reactor 20 °C, maximum pressure inside 120 bar, maximum power 1500 W, duration 15 min. Stage II: gradual increase in the temperature inside the reactor until reaching the temperature of 220 °C at the end of the second stage, maximum pressure inside the reactor 150 bar, maximum power 1500 W, duration 10 min. Stage III: maintaining the temperature at 220 °C, maximum pressure inside the reactor 150 bar, maximum power 1500 W, duration 8 min. After decomposition, the samples were quantitatively transferred into clean 50 mL volumetric flasks. An internal standard of indium solution (ICP class, Merck, Darmstadt, Germany) was added before the final dilution of the samples with deionized water. For this, 2 mL of 10 mg/kg indium solution was introduced into 50 mL flasks. The expected indium concentration in the test solution was therefore 0.4 mg/kg. The use of an internal standard was used to monitor the reproducibility of analyzes performed in a given matrix.

Prior to the quantitative analysis using the ICP-OES technique (iCAP 7400, Thermo Scientific Waltham, MA, USA), calibration curves were prepared for the 1000 mg/kg starting standard, which was a Merck IV multi-element ICP standard (Multi-element ICP standard, Merck). The calibration curve was prepared based on the stock standards from which serial dilutions were prepared. The maximum concentration of the standard was 2 mg/kg and the lowest was 0.02 mg/kg. The Fe levels were monitored using four emission lines for Fe. In all cases, the plasma observation was in Axial mode, similar to the internal standard. The Fe concentration values were finally given against the following emission line: Fe II (ion line) 259.940 nm (Axial), In 325.609 nm (Axial). The correctness of the obtained results was verified, among others, by analyzing the certified river water material WatR Supply Metals 697 (Eraqc) and a satisfactory agreement was obtained between the results for Fe and the certified values. In addition, the reproducibility of the internal standard (In) in the matrix of the analyzed samples was at a satisfactory level. Parameters of the ICP-OES iCAP 7400 spectrometer (Thermo Scientific, Waltham, MA, USA) were as follows: number of replicates: 3, carrier gas 0.5 L/min, plasma gas 12 L/min, auxiliary gas 0.5 L/min, torch: quartz, nebulizer: concentric, quartz, RF generator power: 1150 W, internal standard: Ind, torch setting mode: Axial (Fe, In), resolution: 7 pm at 200 nm, spectral range: 166–847 nm, detector: semiconductor CID, optics: simultaneous analyzer with Echelle optics (52.91 grooves/mm).

### 3.12. Irradiation

**Figure 13:**
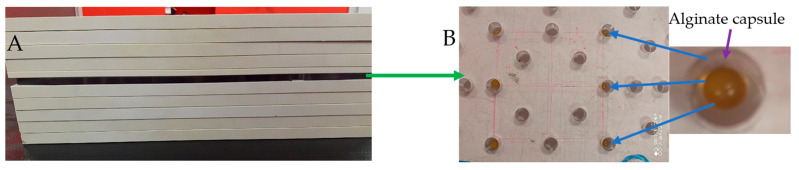
Irradiation of alginate capsules. Phantom SP34 (RW3 plates) (**A**) and a poly(methyl methacrylate) template with holes and capsules inside the holes (**B**).

The capsules were made of medium-viscosity Heppe and low-viscosity Sigma alginates in a reaction of 3.5% alginate with Fricke solution (FAS: 1 mM, XO: 0.165 mM, H_2_SO_4_: 50 mM) containing 3.5% CaCl_2_ by dropwise addition of sodium alginate solution into the Fricke solution with Ca^+2^. They were irradiated with X-rays of a TrueBeam medical accelerator (Varian, Palo Alto, CA, USA). For this purpose, the capsules were placed in holes with a diameter of 5 mm in a template made of poly(methyl methacrylate) with a thickness of 5 mm. This template was located between plates SP34 phantom made of RW3 (IBA, Schwarzenburg, Germany); the thickness of the plates on the top and bottom was 4.5 and 5 cm, respectively (Figure 13). The radiation conditions were as follows: 6 MV (with flattening filter, WFF), monitor unit rate of 600 MU/min, field of irradiation: 20 × 20 cm^2^, source-to-surface distance SSD = 95 cm, dose 2, 3, 5, 7, 10, and 20 Gy. In turn, UV–Vis cuvettes (1 cm optical path) with alginate capsules with Fricke solution in Pluronic F-127 or gelatine matrix were irradiated in a W-1-DD1-2 water phantom (GeVero Co., Lodz, Poland) with a TrueBeam accelerator. The following settings were applied: energy of 6 MV WFF, monitor unit rate of 600 MU/min, field size of 20 × 20 cm^2^, SSD = 95 cm, depth = 5 cm, dose = 20 Gy. The required number of MUs was achieved after the dosimetric measurements were performed with the aid of an ionizing chamber (CC04) with an electrometer (Dose-1), both IBA. Calibration of the accelerator (absolute dose measurements) was carried out using an ionizing chamber (FC 65-G) according to a report published elsewhere [66].

## 4. Conclusions

In this work, a study on the preparation of new chemical systems for ionizing radiation dosimetry is presented. The reaction conditions of sodium alginates with Fricke dosimeter components were analyzed to obtain nano-, micro-, and macro-gels and composites of these and physical gel matrices. Although the concept was generally proven for potential dosimetric applications, the chemical complexity prevented us from obtaining dosimetric products ready for use in daily routine dosimetry applications but opened paths for further research.

The most important is that this work resulted in the following: (i) knowledge on the influence of water impurities on the stability of Fe^2+^ and the optimal conditions related to the stability of the Fricke dosimeter during storage was achieved, (ii) proof of the reaction of Fe^2+^ with alginate chains leading to the formation of macro-gels and hydrogel beads, (iii) the possibility of loading the beads with the components of the Fricke dosimeter, (iv) proof of the reactivity of such beads to irradiation, which was characterized by the formation of a color dependent on the absorbed dose, (v) the formation of macro-gels responsive to ionizing radiation, (vi) the conditions of the physical gel matrix allowing the beads to maintain their original shape, and (vii) the possible formation of nano- and micro-gels by the reaction of the Fricke components with the alginate chains. However, the competition between the oxidation of ferrous ions, their binding by alginate chains, and the protonation of the alginate salt at acidic pH makes the system based on nano- and micro-gels less attractive and less suitable for the formation of 3D gel matrices with such structures inside in order to improve the performance of such a 3D dosimeter in terms of the integrity of the recorded dose distribution (reduced diffusion of ferrous and ferric ions). The concept of binding Fe ions to polymer chains is attractive to enlarge the entire structure and reduce Fe diffusion. It requires further testing using different compounds, including polymers.

The studies presented in this work indicate the necessity of modifying Fricke beads/capsules to impede the leakage of ferrous and ferric ions and their complexes with XO; a layer-by-layer approach using polymers not investigated in detail in this work could be an option. The concept of capsules containing Fricke dosimetry or further modification of such capsules containing other radiation-sensitive compounds is very intriguing. Such solutions can be considered as an alternative to thermoluminescence dosimeters (TLD). TLDs are sometimes used in combination with anthropomorphic phantoms, where they are placed in holes and irradiated. The same would be done with the dosimetric capsules. Unlike TLDs, the capsules change color, and the reading of the color is much less complicated than reading the signal collected by TLDs, which require special treatment after irradiation. The formation of macro-gels by the reaction of ferrous ions with alginate macromolecules requires optimization towards smooth and uniform 3D hydrogels, which can then find application in 3D radiotherapy dosimetry.

## Figures and Tables

**Figure 2:**
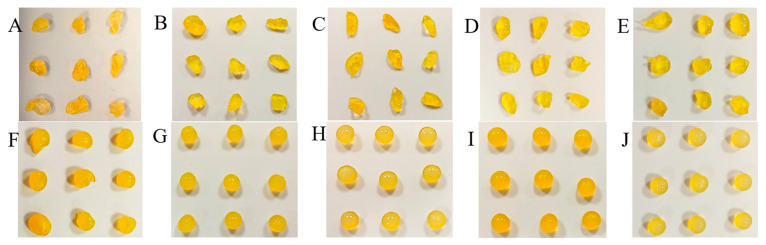
Alginate capsules obtained by dropping the sodium alginate polymer solution of a low viscosity (Sigma) into the Fricke solution containing CaCl_2_ of 0% (**A**,**F**), 1% (**B**,**G**), 1.5% (**C**,**H**), 2.5% (**D**,**I**), and 3.5% (**E**,**J**). The concentration of the sodium alginate solution was as follows: 1.7% (**A**–**E**), 3.5% (**F**–**J**).

**Figure 3:**
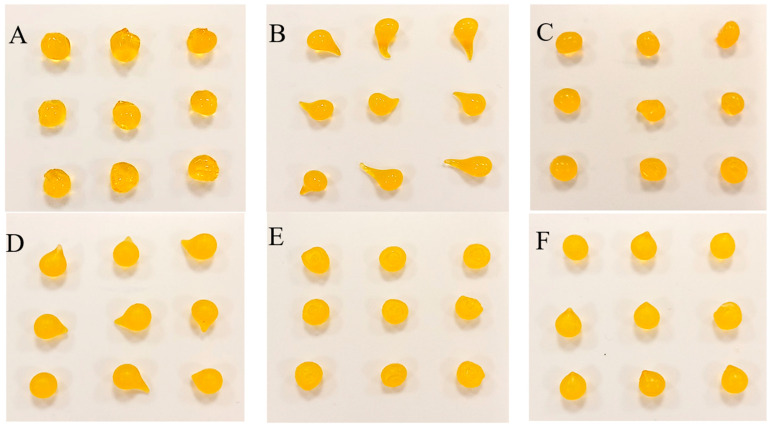
Alginate capsules obtained by dropping the sodium alginate polymer solution of medium viscosity (Sigma) into the Fricke solution containing CaCl_2_ of 0% (**A**,**D**), 1% (**B**,**E**), and 1.5% (**C**,**F**). The concentration of the sodium alginate solution was as follows: 1.7% (**A**–**C**), 3.5% (**D**–**F**).

**Figure 4:**
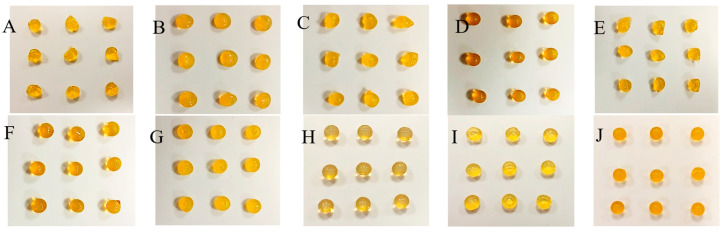
Alginate capsules obtained by dropping the sodium alginate polymer solution (medium viscosity, Heppe) into the Fricke solution containing CaCl_2_ of 0% (**A**,**F**), 1% (**B**,**G**), 1.5% (**C**,**H**), 2.5% (**D**,**I**), and 3.5% (**E**,**J**). The concentration of the sodium alginate solution was as follows: 1.7% (**A**–**E**), 3.5% (**F**–**J**).

**Table 1:** Characterization of alginate capsules made from sodium alginate reaction with Fricke and CaCl_2_ solutions.

No.	Alginate Type	Alginate Concentration [%]	CaCl_2_ Concentration [%]	Comment
1	Low viscosity, Sigma	2.7	2.5	Acceptable
2			3.5	
3		3.0	2.5	Very good
4			3.5	
5		3.5	1.5	Acceptable
6			2.5	Very good
7			3.5	
8	Medium viscosity, Sigma	2.7	1.5	Acceptable
9	Medium viscosity, Heppe	2.7	1.5	Very good
10			2.5	
11		3.0	1	Acceptable
12			1.5	Very good
13			2.5	
14			3.5	
15		3.5	1.5	
16			2.5	
17			3.5	

**Table 2:** Types of water used in the study and their characteristics.

Type of Water	Water Hardness [°dH]	pH [-]	Conductivity [µS/cm]
Tap water	12.17	7.46	378.9
Distilled	0.35	6.95	11.15
Re-distilled	-	5.77	1.23
Deionized	-	5.63	1.16

## Data Availability

The original contributions presented in this study are included in the article/Supplementary Material. Further inquiries can be directed to the corresponding author.

## Supplementary Materials

The supporting information can be downloaded at: https://www.mdpi.com/article/10.3390/ijms26146603/s1.
