# Supplementary material for: Radiation-Sensitive Nano-, Micro-, and Macro-Gels and Polymer Capsules for Use in Radiotherapy Dosimetry
Source: Int J Mol Sci. 2025 Jul 10;26(14):6603. doi: 10.3390/ijms26146603 (PMC12294773; doi:10.3390/ijms26146603)
Supplement: Supplementary file 1 [file ijms-26-06603-s001.zip › ijms-3722341-supplementary.pdf]

Supplementary material

Abstract

This work introduces an original approach to the manufacturing of ionizing radiation-sensitive systems for radiotherapy applications - dosimetry. They are based on the Fricke dosimetric solution and the formation of macro-gels and capsules, and nano- and micro-gels. The reaction of ionic polymers, such as sodium alginate, with Fe and Ca metal ions is employed. Critical polymer concentration (c*) is taken as the criterion. Reaction of ionic polymers with metal ions leads to products related to c*. Well below c*, nano- and micro-gels may form. Above c* macro-gels and capsules can be prepared. Nano- and micro-gels containing Fe in the composition can be used for infusion of a physical gel matrix to prepare 2D or 3D dosimeters. In turn, macro-gels can be formed with Fe ions crosslinking polymer chains to obtain radiation-sensitive hydrogels, so called from wall-to-wall, serving as 3D dosimeters. The encapsulation process can lead to capsules with Fe ions serving as 1D dosimeters. This work presents the concept of manufacturing various gel structures, their main features and manufacturing challenges. It proposes new directions of research towards novel dosimeters.

Keywords: Fricke gel dosimeter; Radiotherapy dosimeter; 3D dosimeter; Microgels; Macrogels; Ionizing radiation

1. Introduction

See the main text of the publication.

2. Materials and methods

See the main text of the publication.

3. Results and discussion

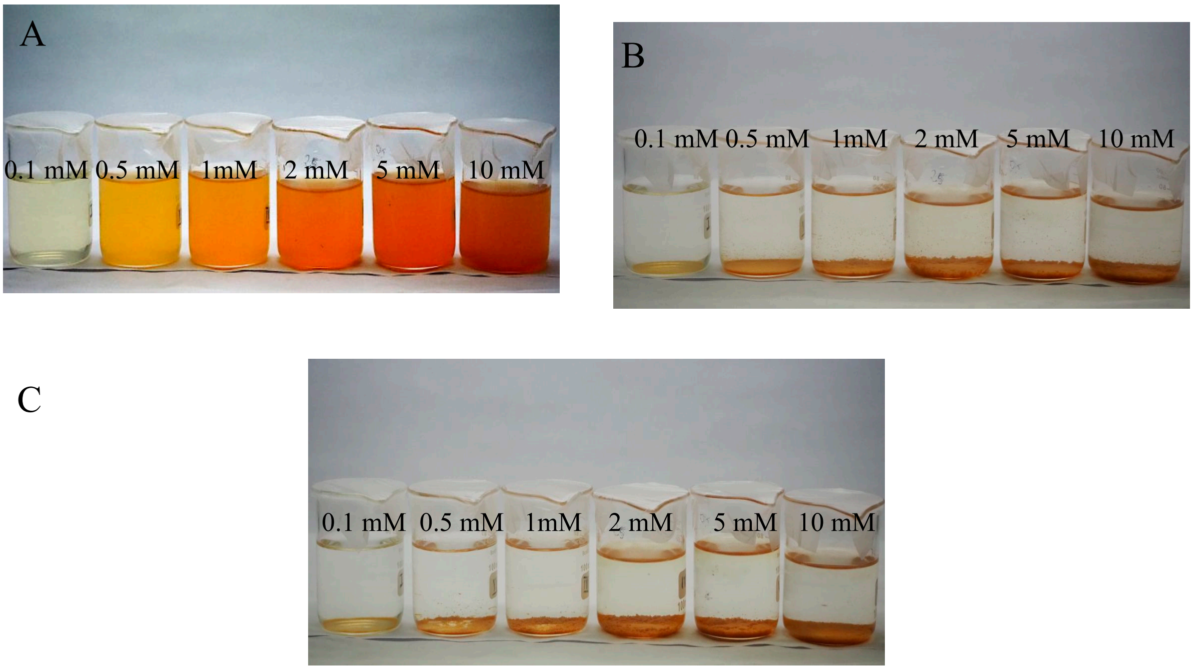

Supplementary Figure S1. Photographs of 0.1–10 mM FAS solutions after preparation (0 h) (A) and storage for 24 h (B) and 48 h (C) in a refrigerator (~4 °C) for the solutions prepared in tap water.

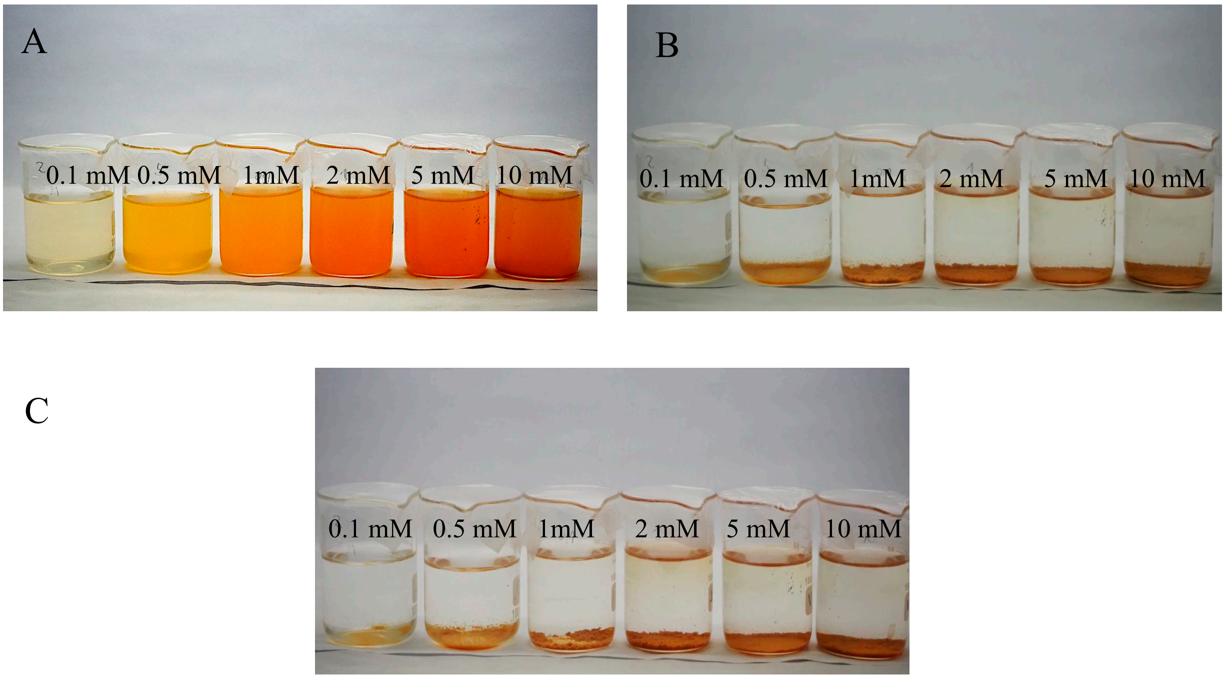

Supplementary Figure S2. Photographs of 0.1–10 mM FAS solutions after preparation (0 h) (A) and storage for 24 h (B) and 48 h (C) at room temperature (~21–23° C) for the solutions prepared in tap water.

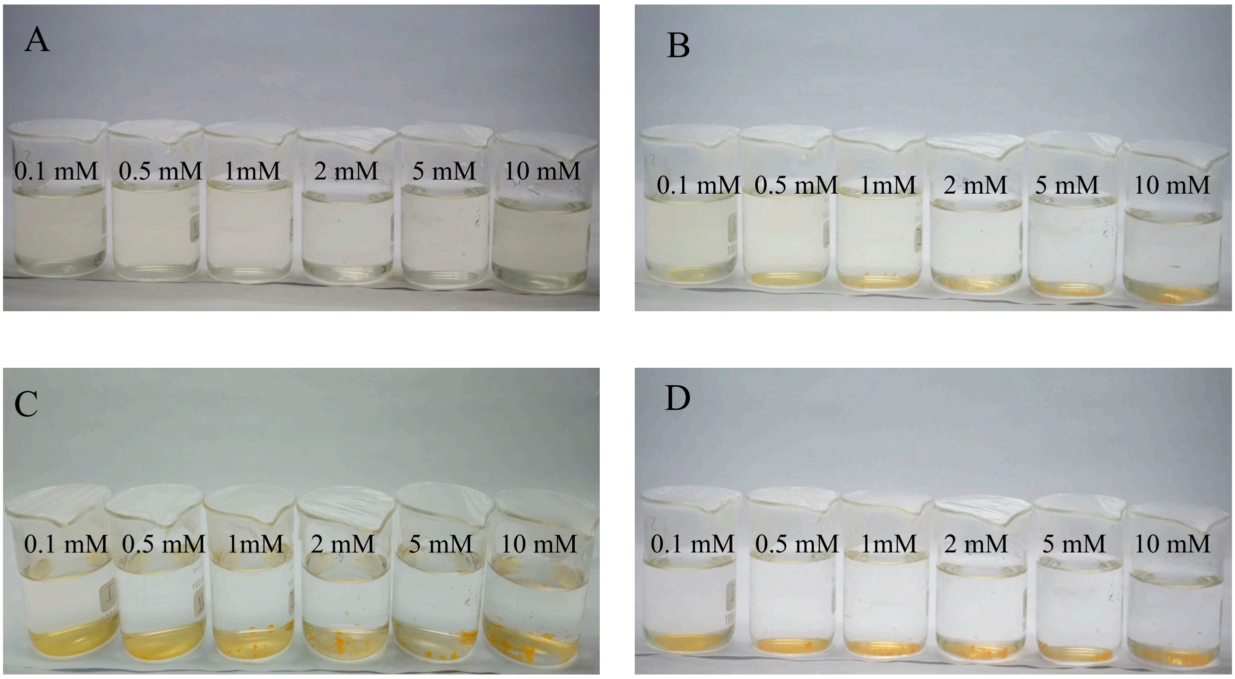

Supplementary Figure S3. Photographs of 0.1–10 mM FAS solutions after preparation (0 h) (A) and storage for 24 h (B), 72 h (C), 168 h (D) in a refrigerator (~4 °C) for the solutions prepared in distilled water.

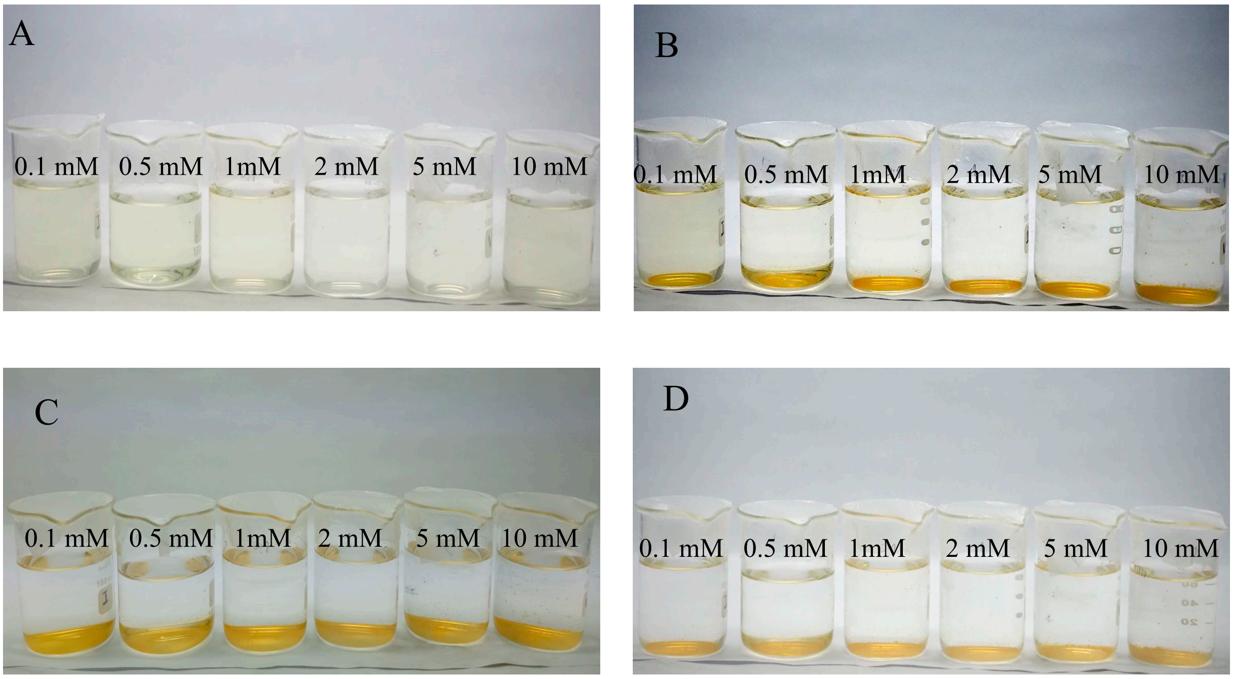

Supplementary Figure S4. Photographs of 0.1–10 mM FAS solutions after preparation (0 h) (A) and storage for 24 h (B), 72 h (C) and 168 h (D) at room temperature (~21–23° C) for the solutions prepared in distilled water.

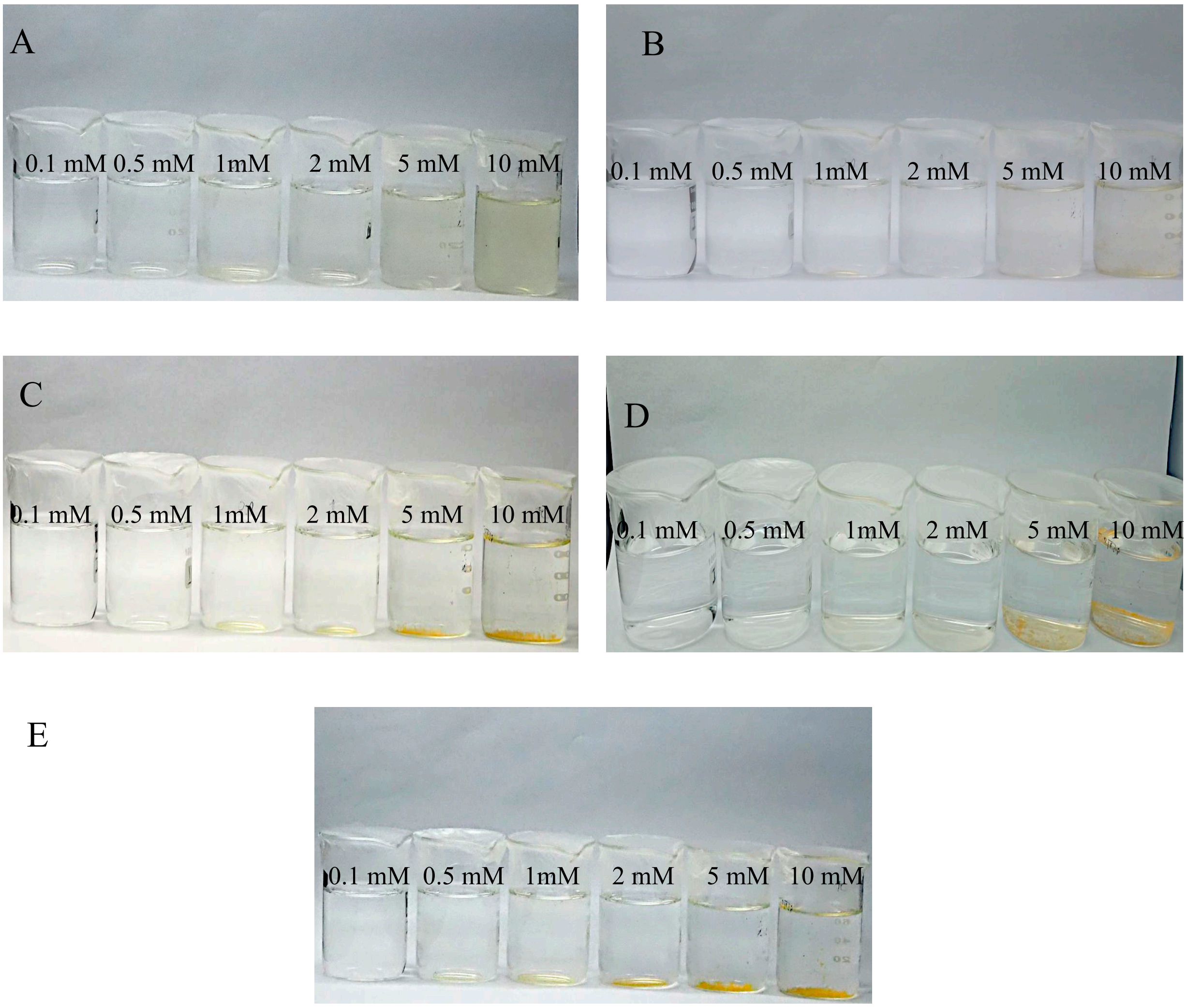

Supplementary Figure S5. Photographs of 0.1–10 mM FAS solutions after preparation (0 h) (A) and storage for 24 h (B), 48 h (C), 72 h (D), 168 h (E) in a refrigerator (~4 °C) for the solutions prepared in re-distilled water.

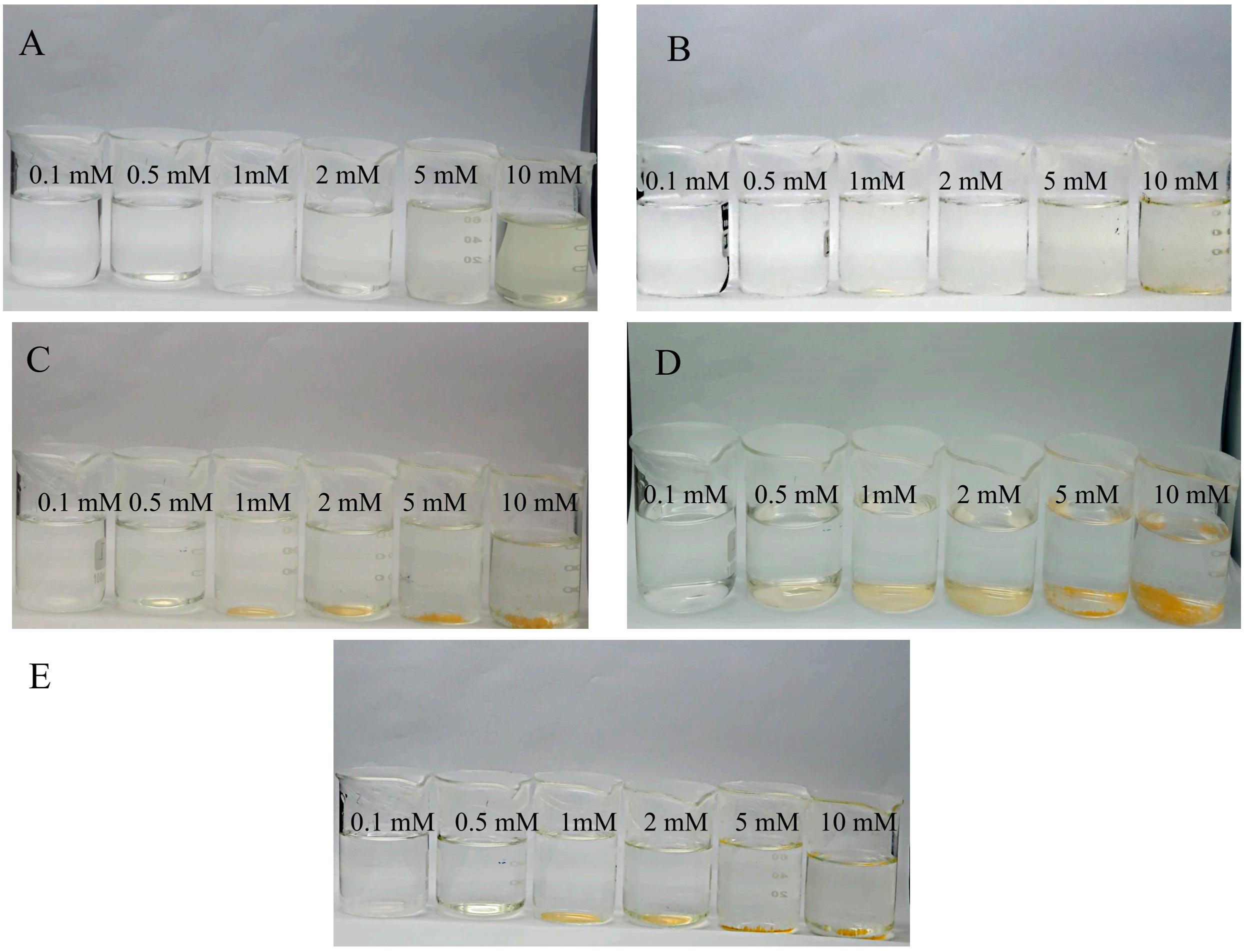

Supplementary Figure S6. Photographs of 0.1–10 mM FAS solutions after preparation (0 h) (A) and storage for 24 h (B), 72 h (C) and 168 h (D) at room temperature (~21–23° C) for the solutions prepared in re-distilled water.

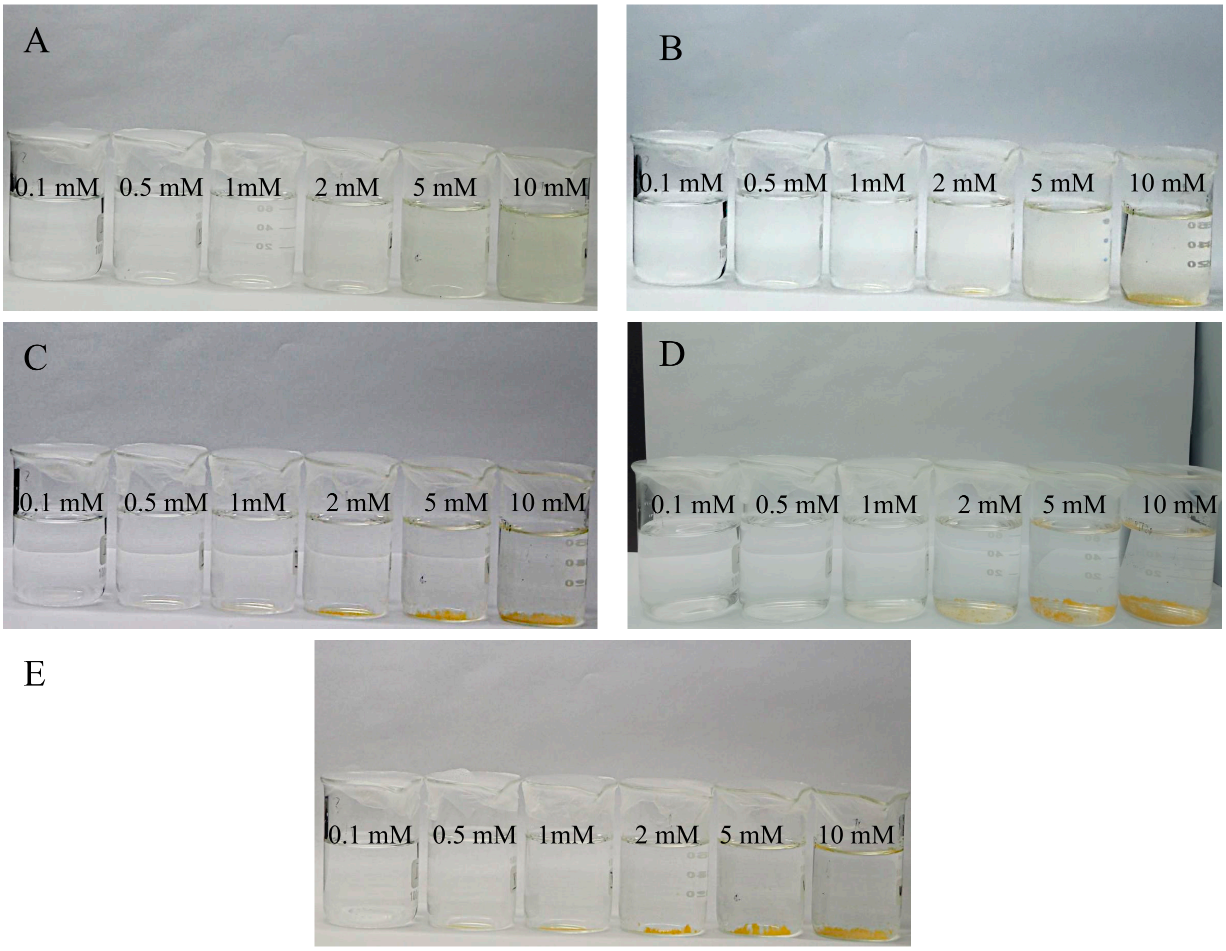

Supplementary Figure S7. Photographs of 0.1–10 mM FAS solutions after preparation (0 h) (A) and storage for 24 h (B), 48 h (C), 72 h (D), 168 h (E) in a refrigerator (~4 °C) for the solutions prepared in deionized water.

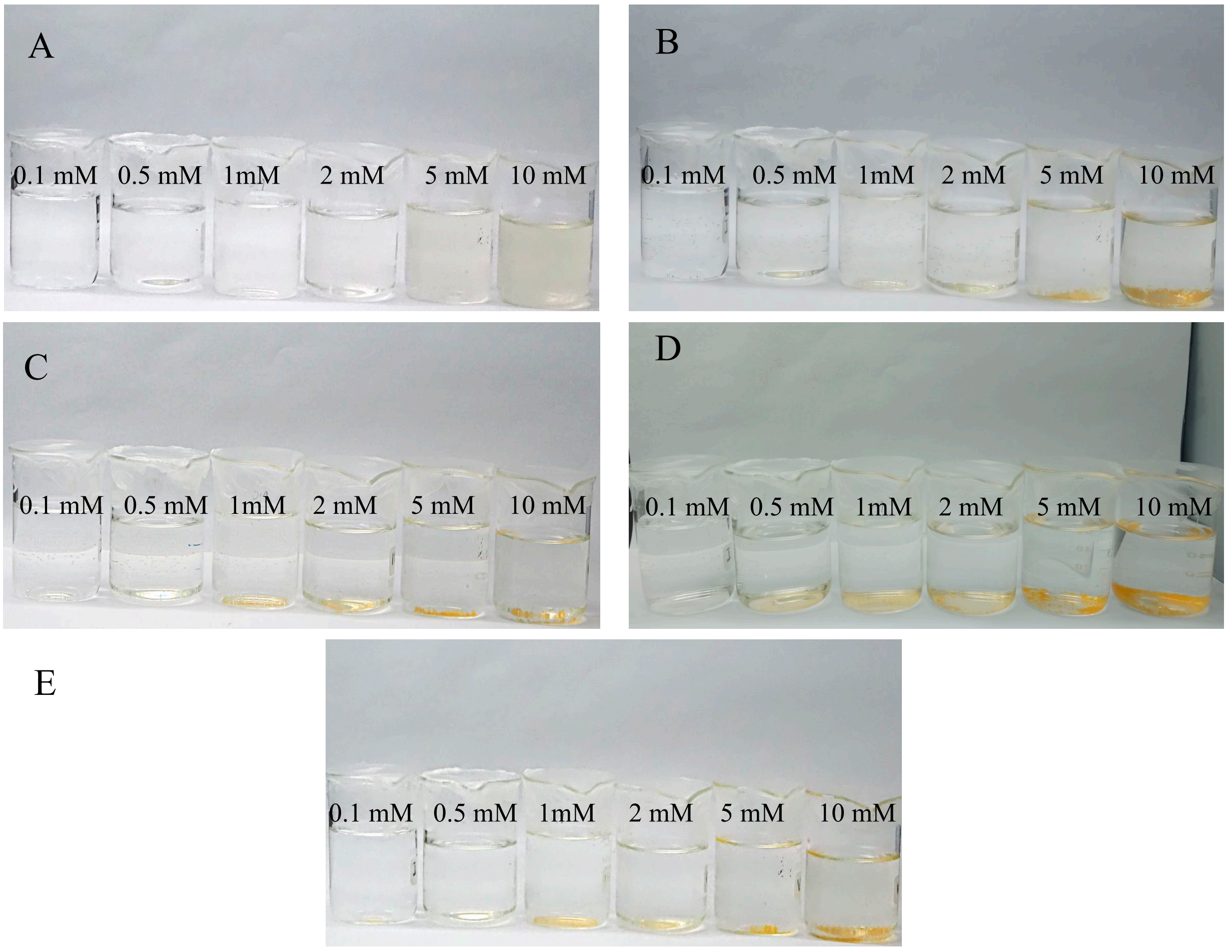

Supplementary Figure S8. Photographs of 0.1–10 mM FAS solutions after preparation (0 h) (A) and storage for 24 h (B), 48 (C), 72 h (D) and 168 h (E) at room temperature (~21–23° C) for the solutions prepared in deionized water.

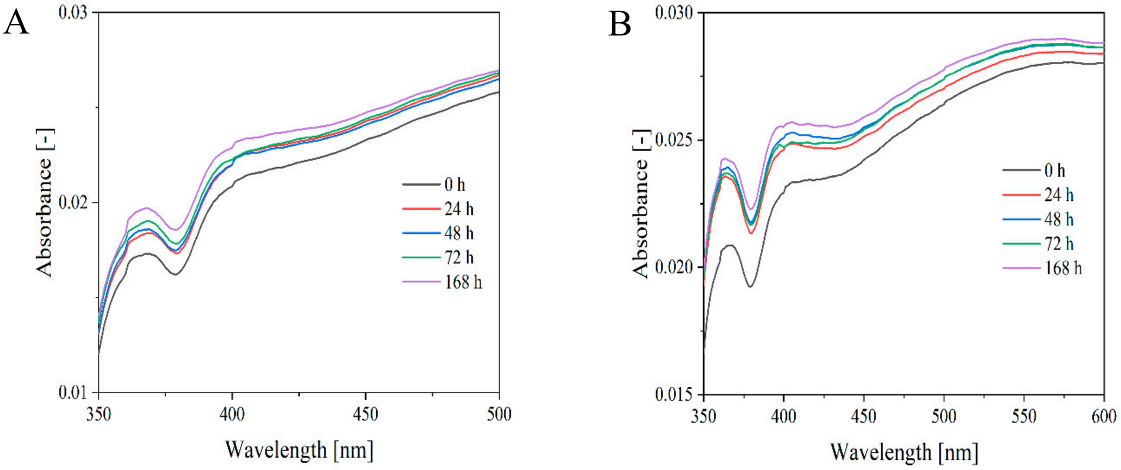

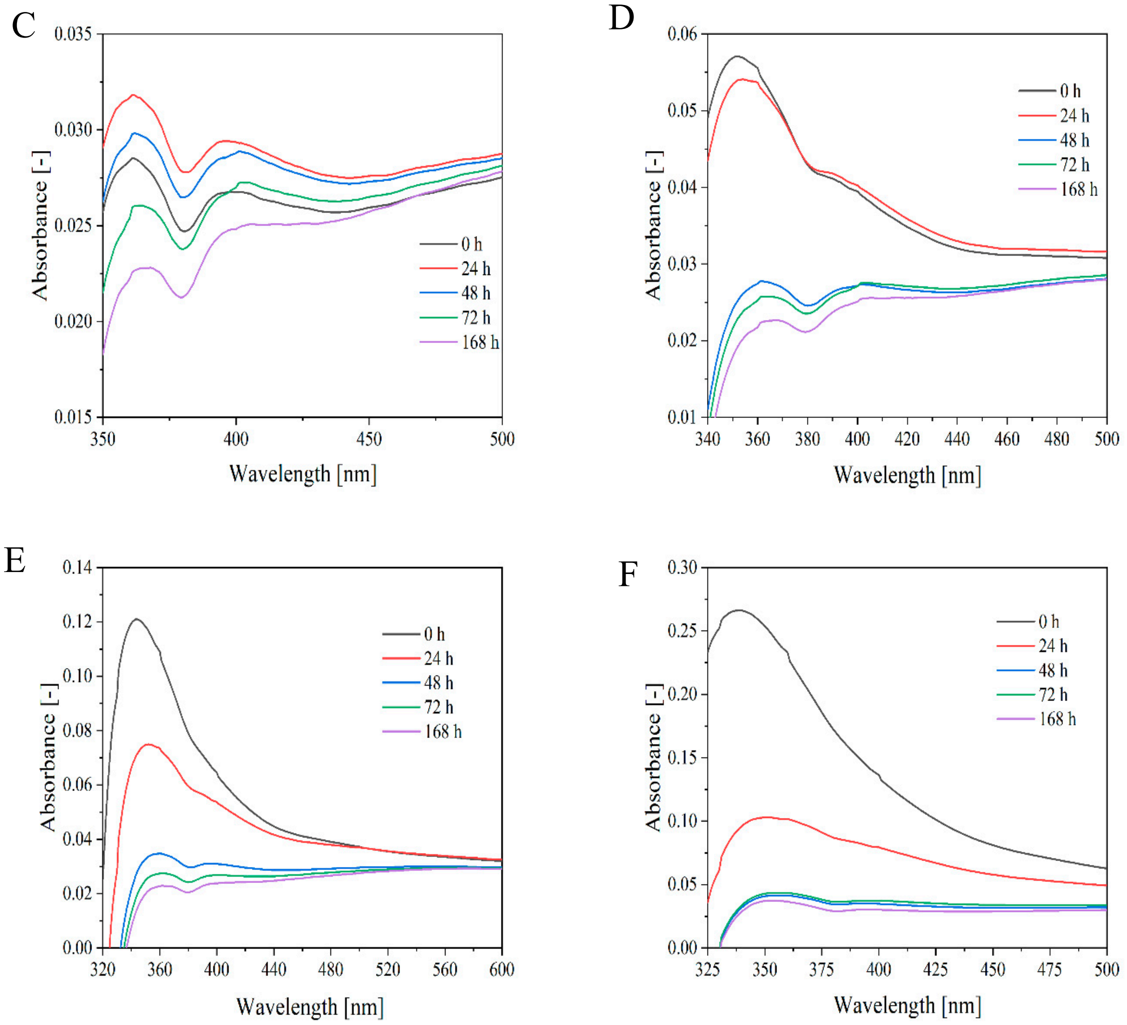

Supplementary Figure S9. Absorption spectra of FAS solutions in deionized water and stored in the refrigerator (~4 °C): 0.1 mM (A), 0.5 mM (B), 1 mM (C), 2 mM (D), 5 mM (E) and 10 mM (F).

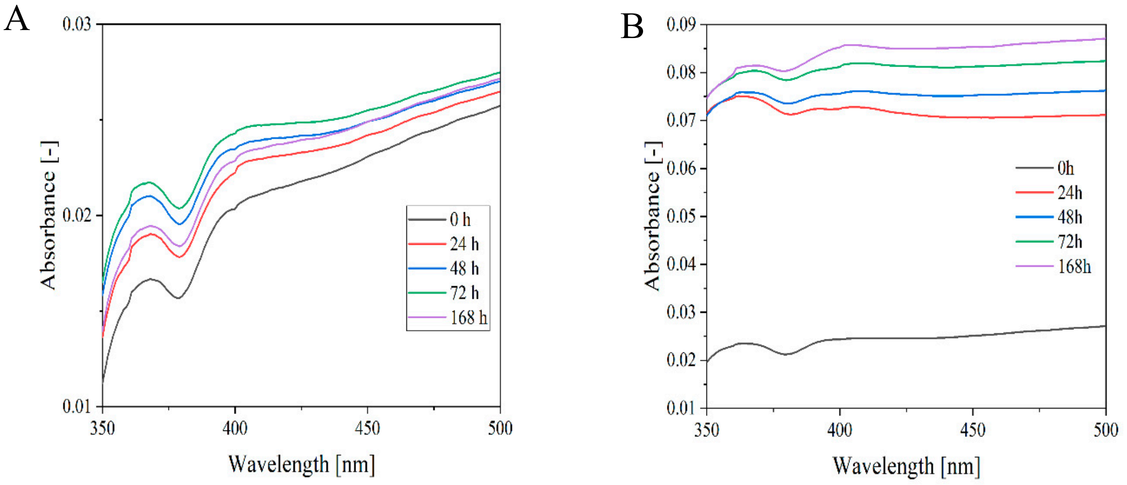

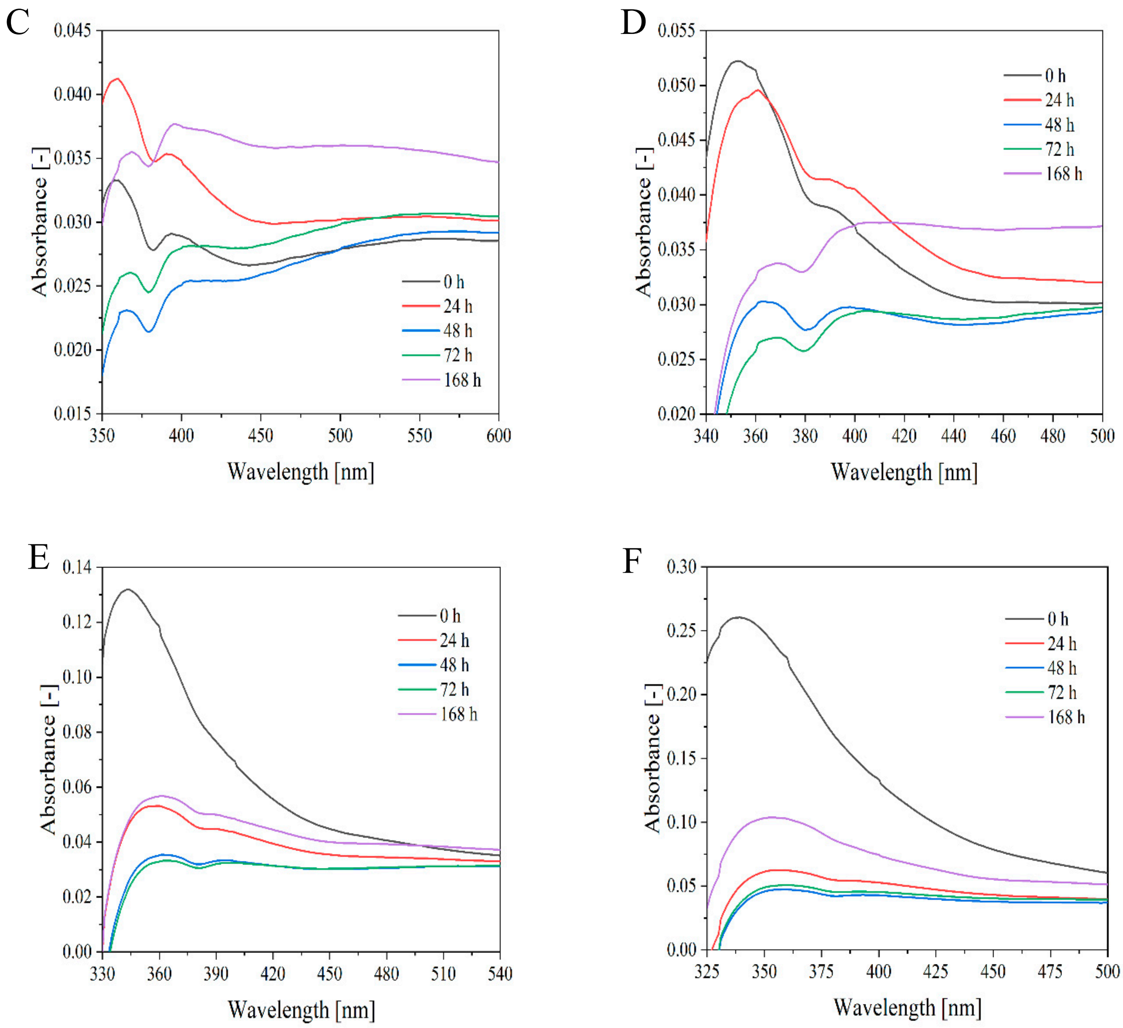

Supplementary Figure S10. Absorption spectra of FAS solutions in deionized water and stored in a cabinet (~21–23° C): 0.1 mM (A), 0.5 mM (B), 1 mM (C), 2 mM (D), 5 mM (E) and 10 mM (F).

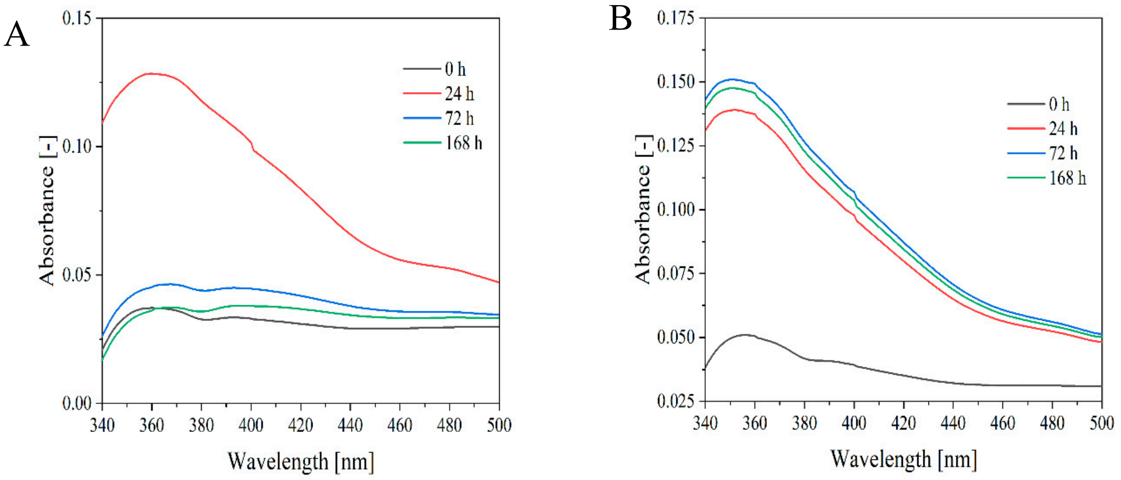

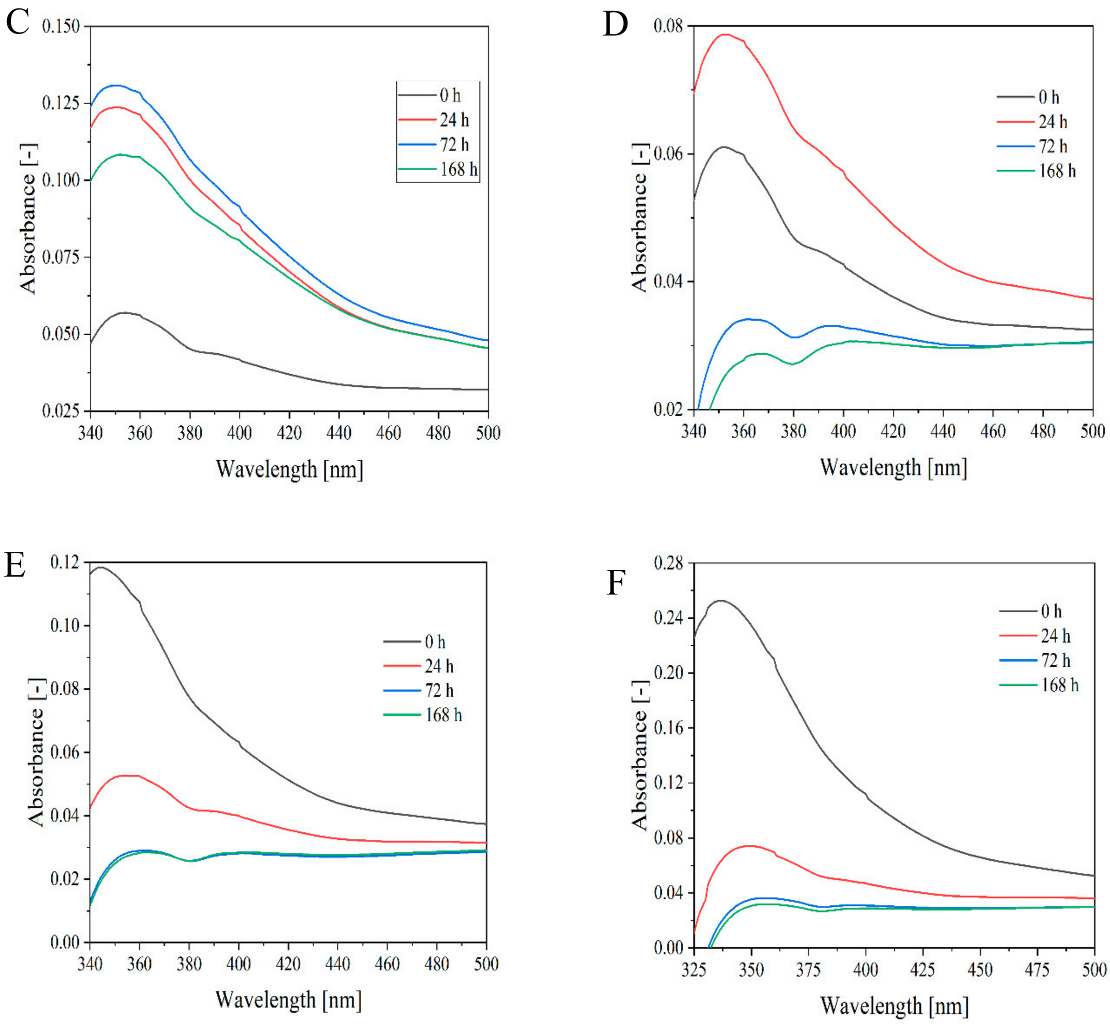

Supplementary Figure S11. Absorption spectra of FAS solutions in distilled water and stored in the refrigerator (~4 °C): 0.1 mM (A), 0.5 mM (B), 1 mM (C), 2 mM (D), 5 mM (E) and 10 mM (F).

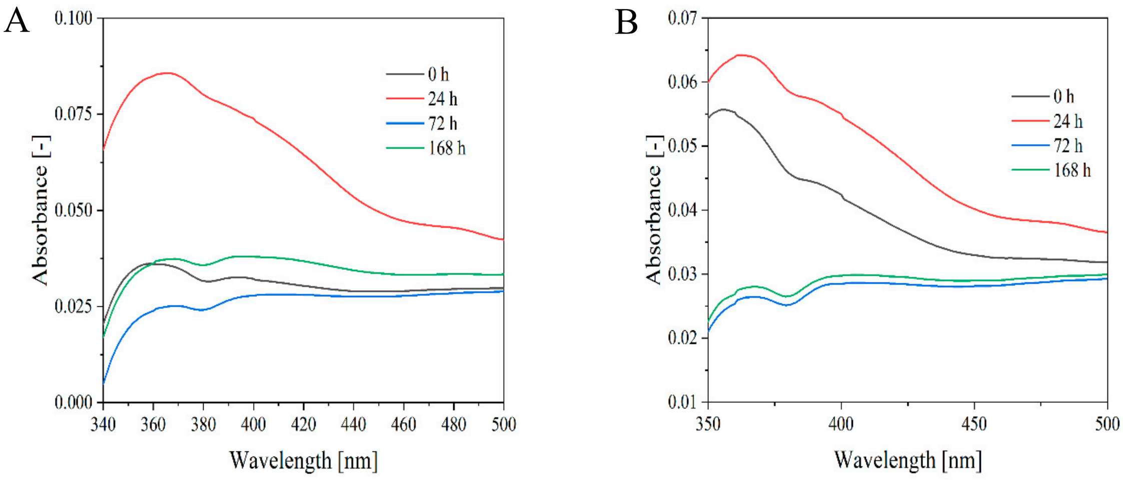

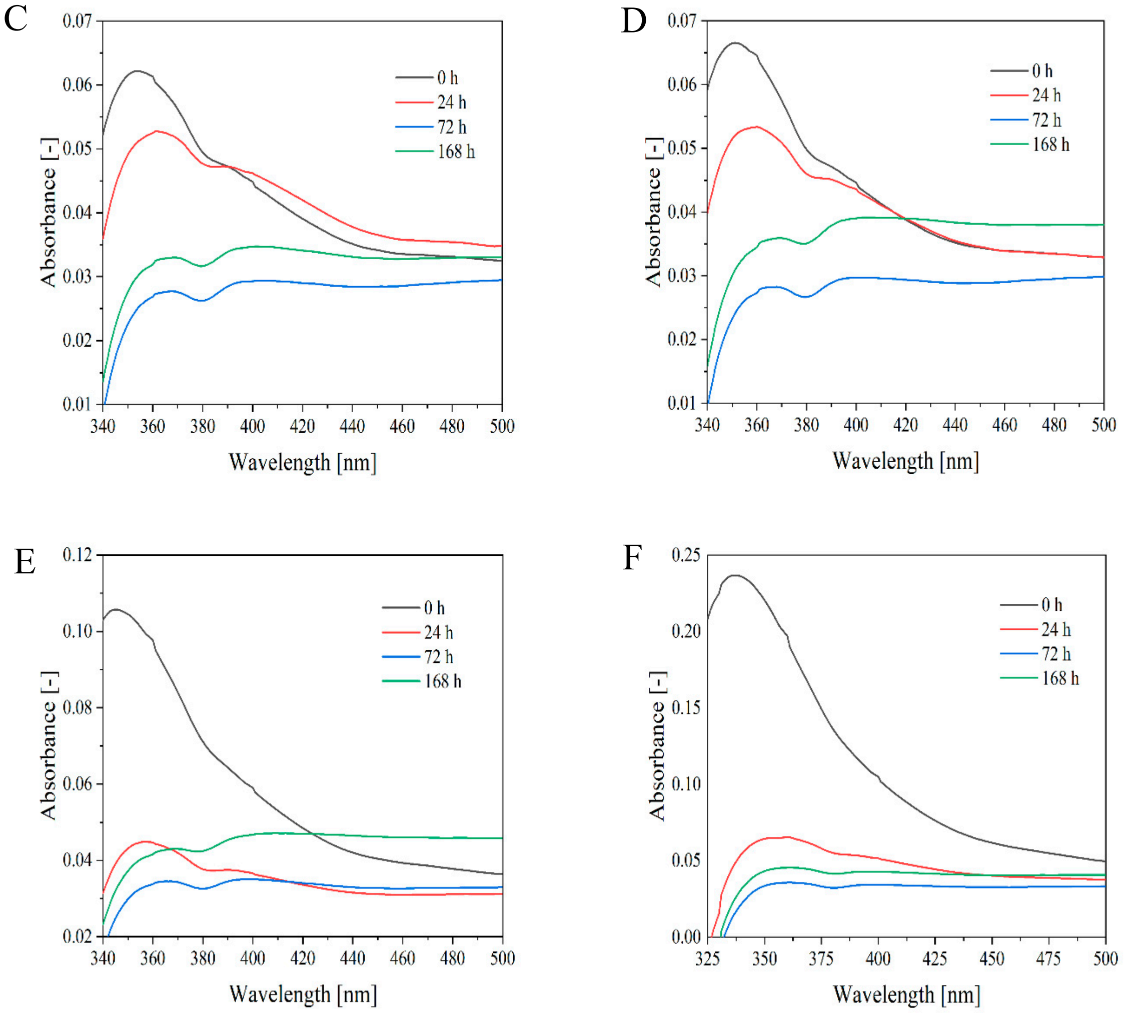

Supplementary Figure S12. Absorption spectra of FAS solutions in distilled water and stored in a cabinet (~21–23° C): 0.1 mM (A), 0.5 mM (B), 1 mM (C), 2 mM (D), 5 mM (E) and 10 mM (F).

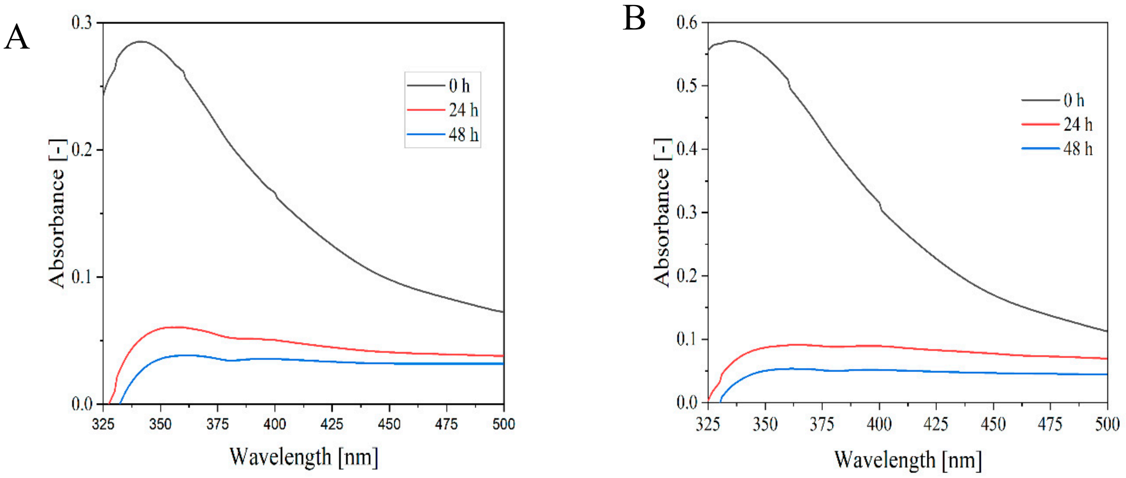

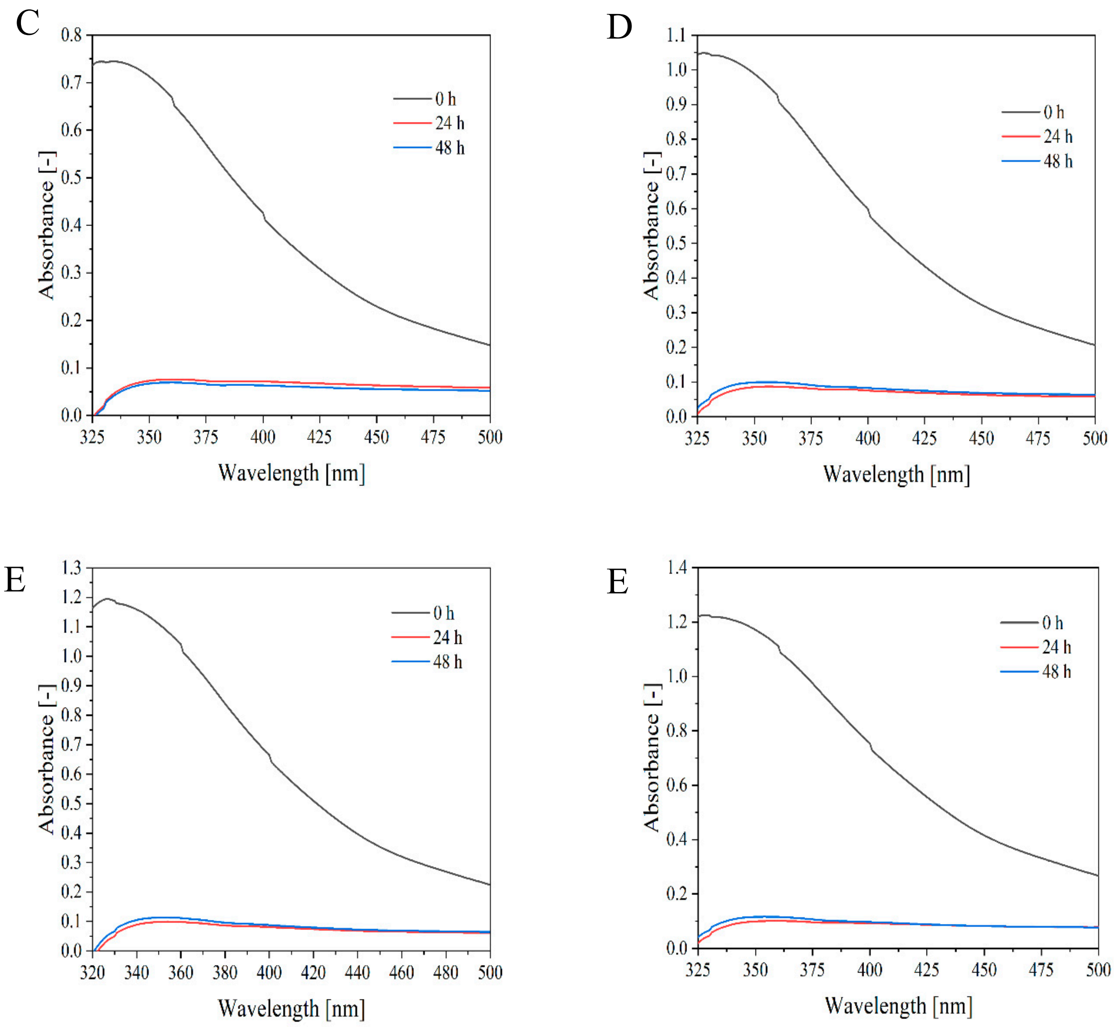

Supplementary Figure S13. Absorption spectra of FAS solutions in tap water and stored in the refrigerator (~4 °C): 0.1 mM (A), 0.5 mM (B), 1 mM (C), 2 mM (D), 5 mM (E) and 10 mM (F).

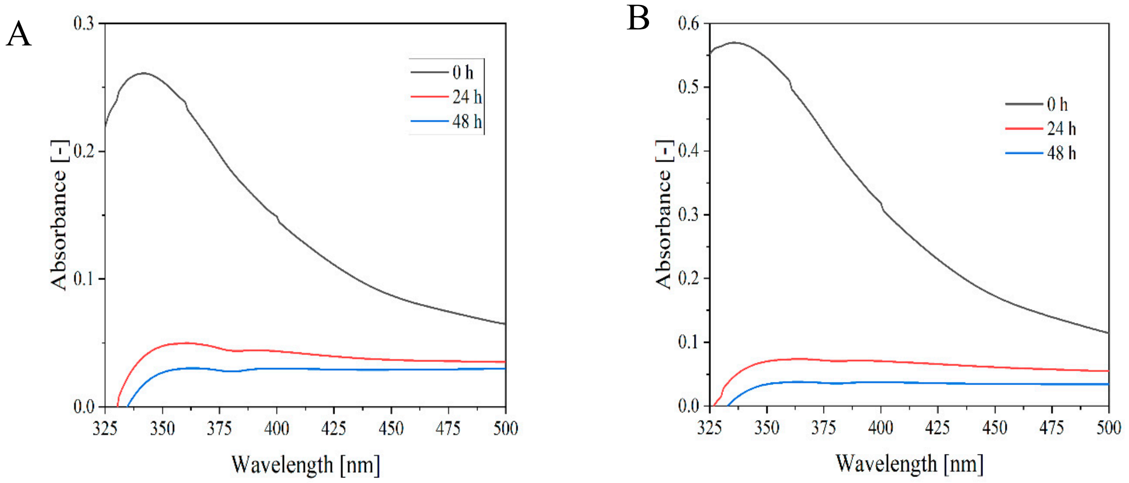

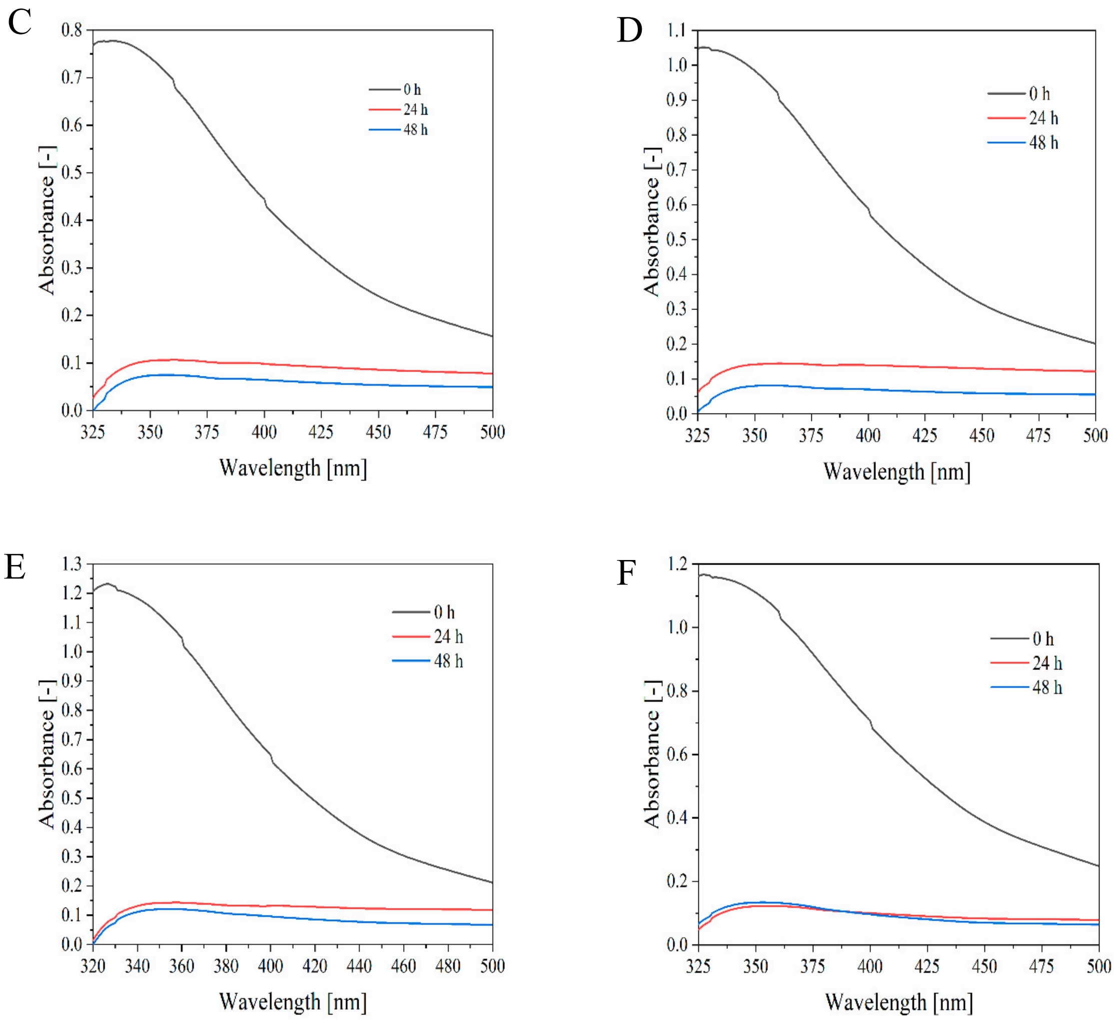

Supplementary Figure S14. Absorption spectra of FAS solutions in tap water and stored in a cabinet (~21–23° C): 0.1 mM (A), 0.5 mM (B), 1 mM (C), 2 mM (D), 5 mM (E) and 10 mM (F).

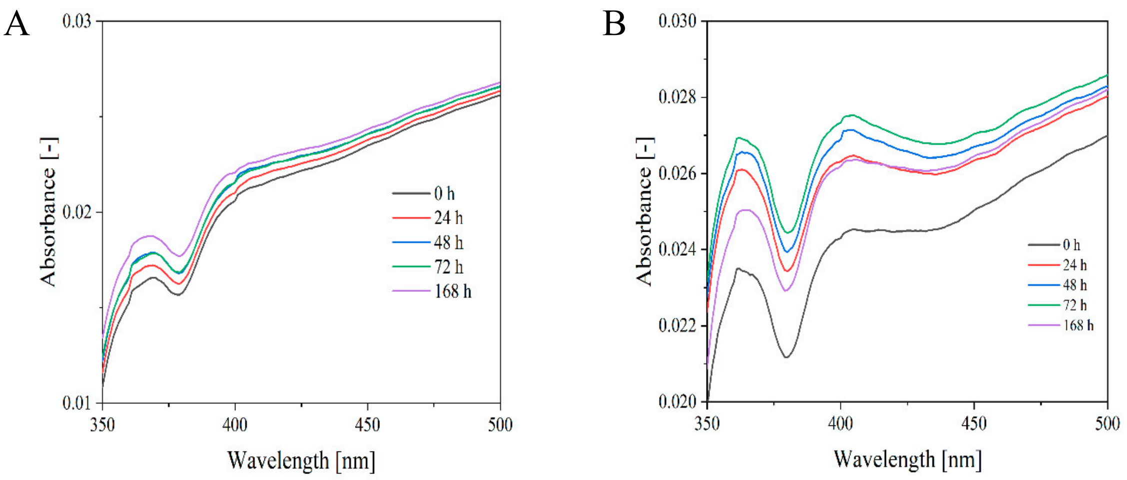

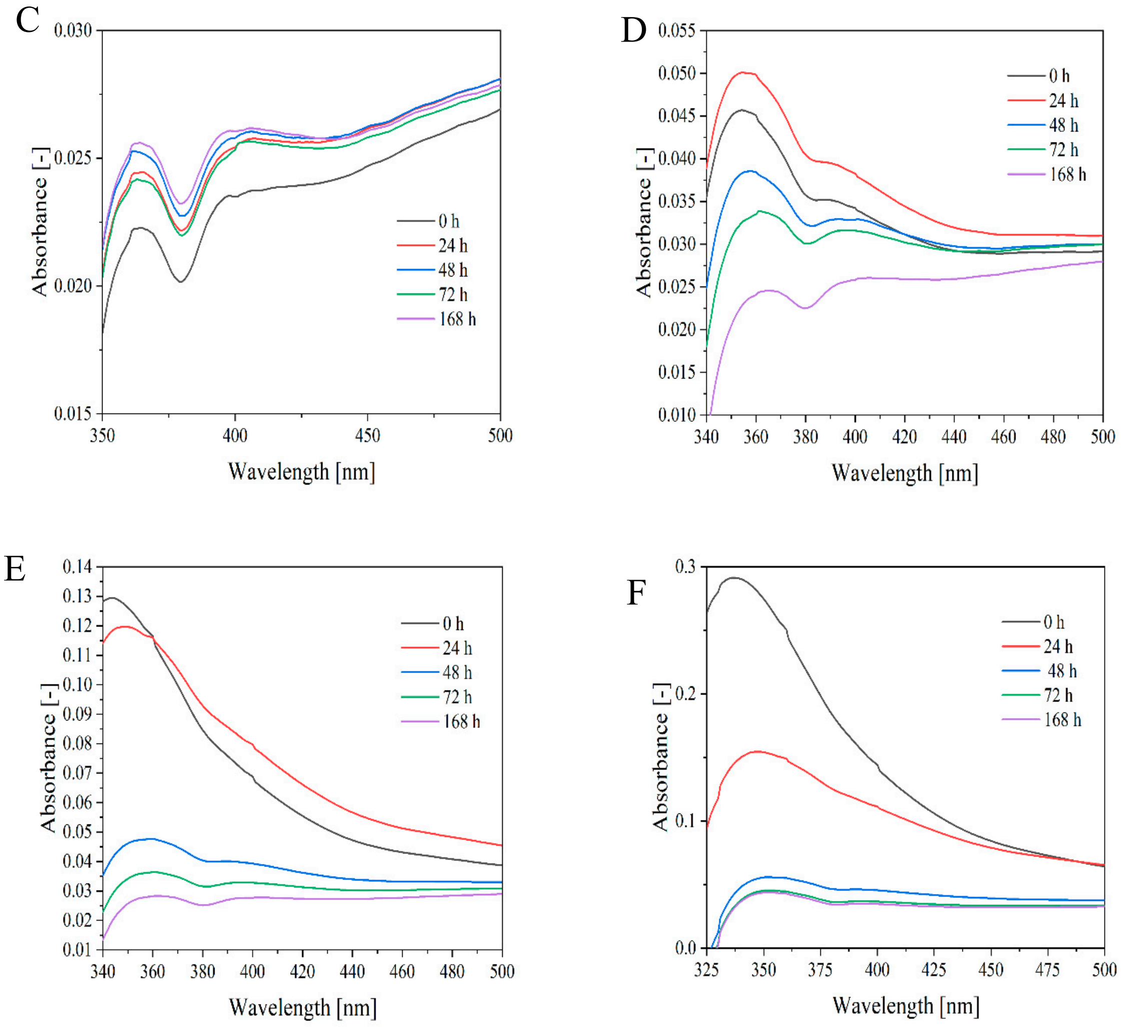

Supplementary Figure S15. Absorption spectra of FAS solutions in redistilled water and stored in the refrigerator (~4 °C): 0.1 mM (A), 0.5 mM (B), 1 mM (C), 2 mM (D), 5 mM (E) and 10 mM (F).

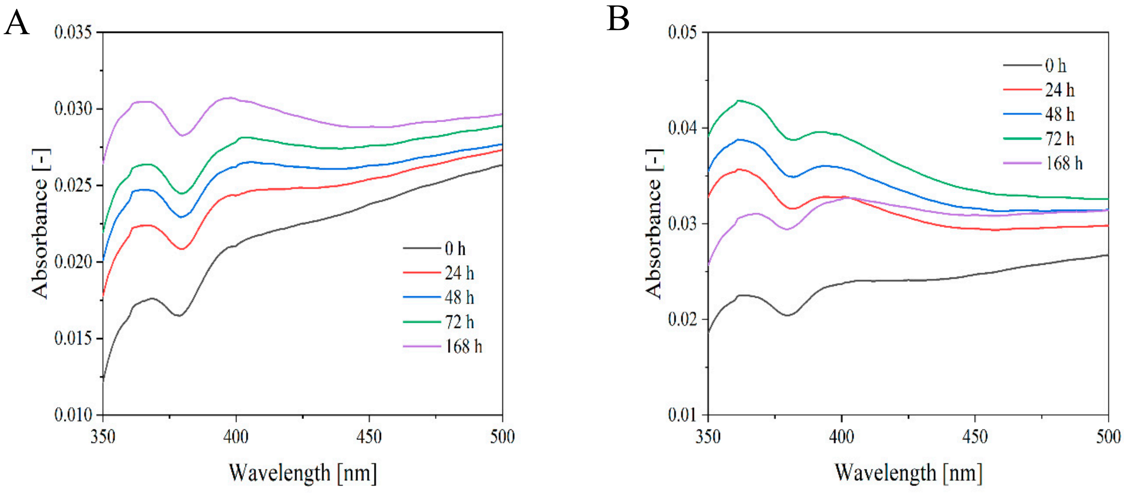

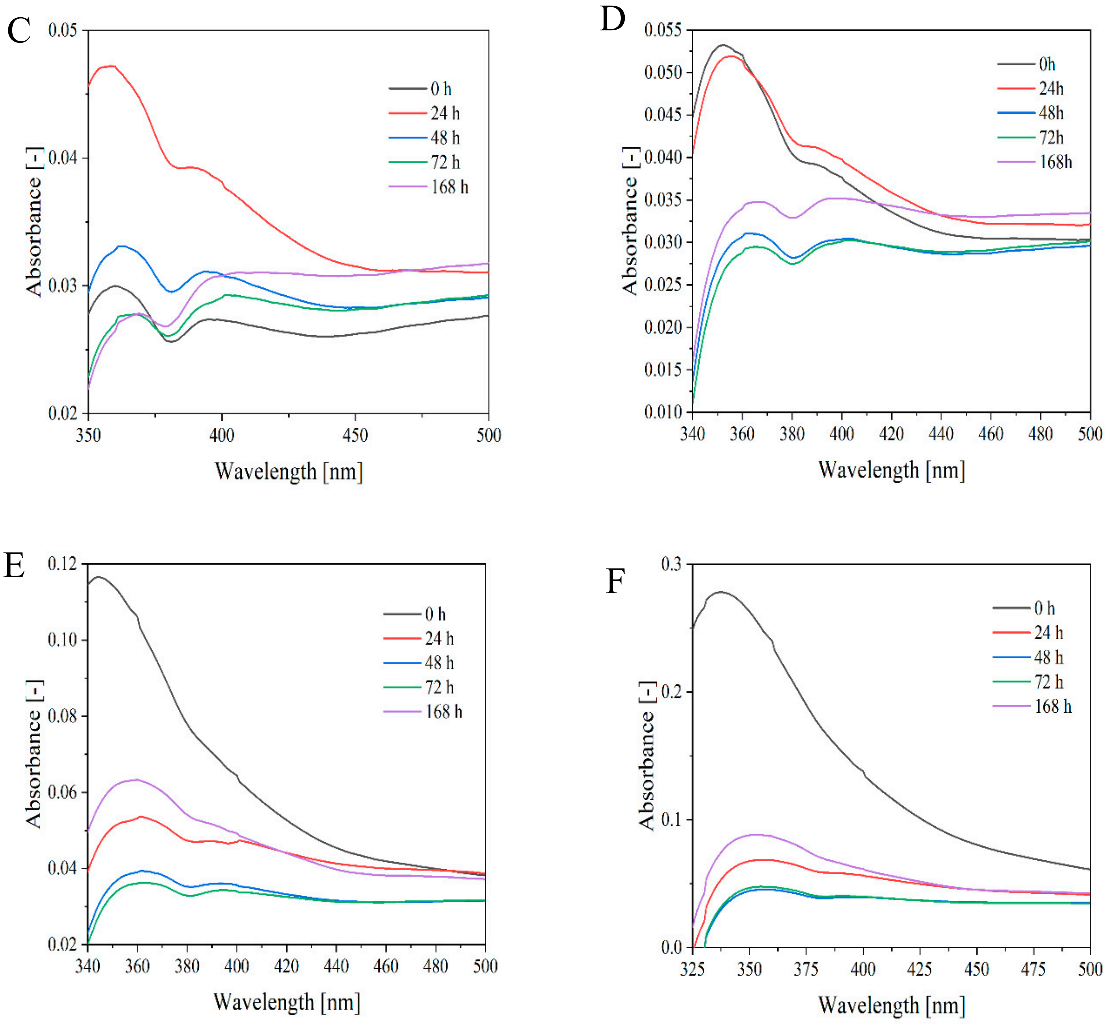

Supplementary Figure S16. Absorption spectra of FAS solutions in redistilled water and stored in a cabinet (~21–23° C): 0.1 mM (A), 0.5 mM (B), 1 mM (C), 2 mM (D), 5 mM (E) and 10 mM (F).

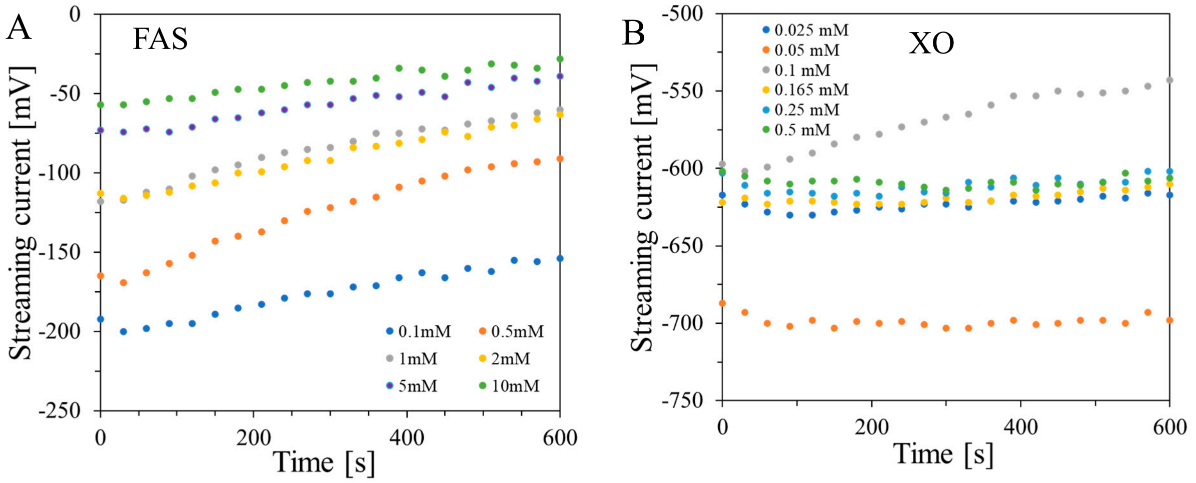

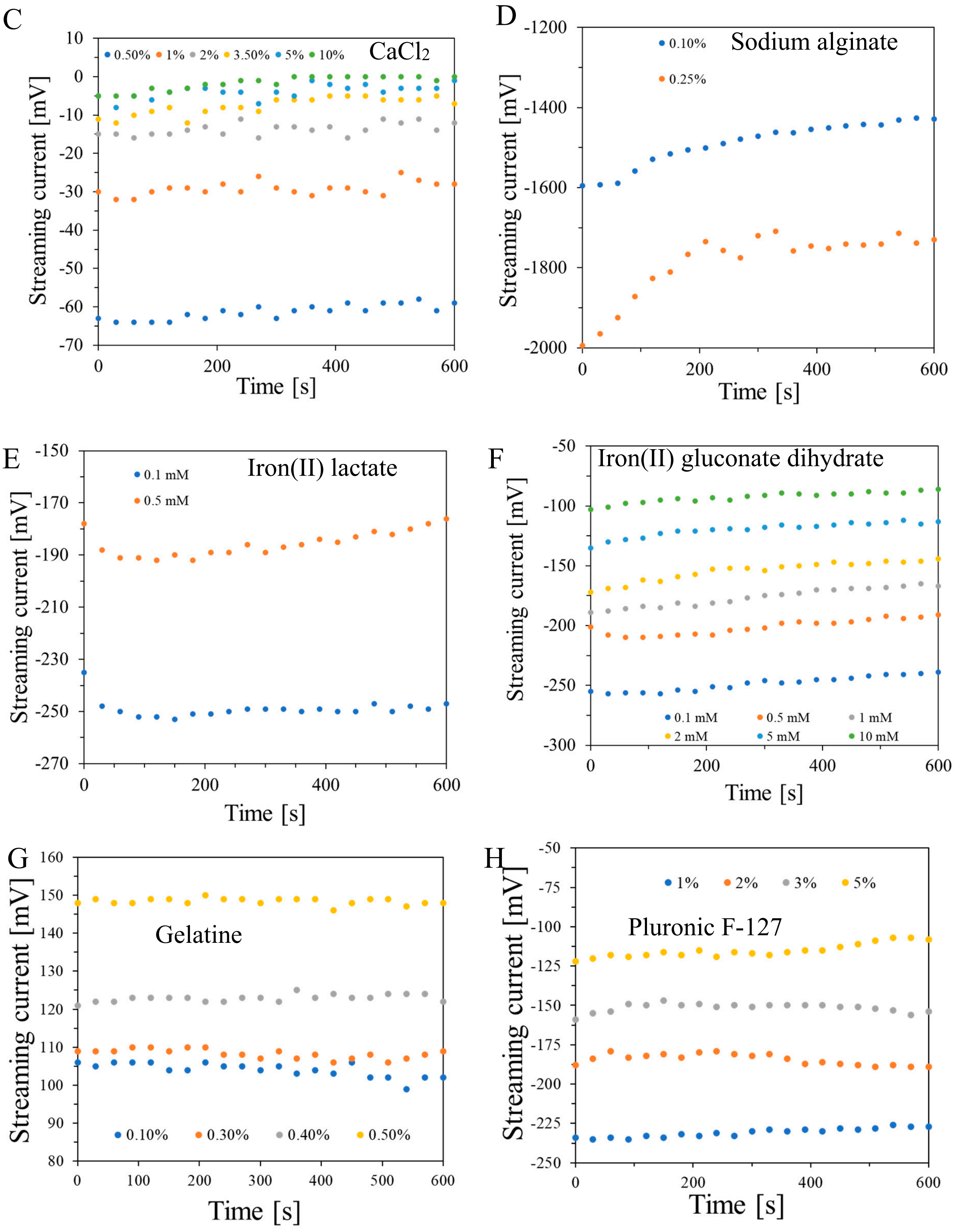
C
CaCl2
0.50% 1% 2% 3.50% 5% 10%
Streaming current [mV]
Time [s]
D
Sodium alginate
0.10%
0.25%
Streaming current [mV]
Time [s]
E
Iron(II) lactate
0.1 mM
0.5 mM
Streaming current [mV]
Time [s]
F
Iron(II) gluconate dihydrate
0.1 mM 0.5 mM 1 mM
2 mM 5 mM 10 mM
Streaming current [mV]
Time [s]
G
Gelatine
0.10% 0.30% 0.40% 0.50%
Streaming current [mV]
Time [s]
H
Pluronic F-127
1% 2% 3% 5%
Streaming current [mV]
Time [s]

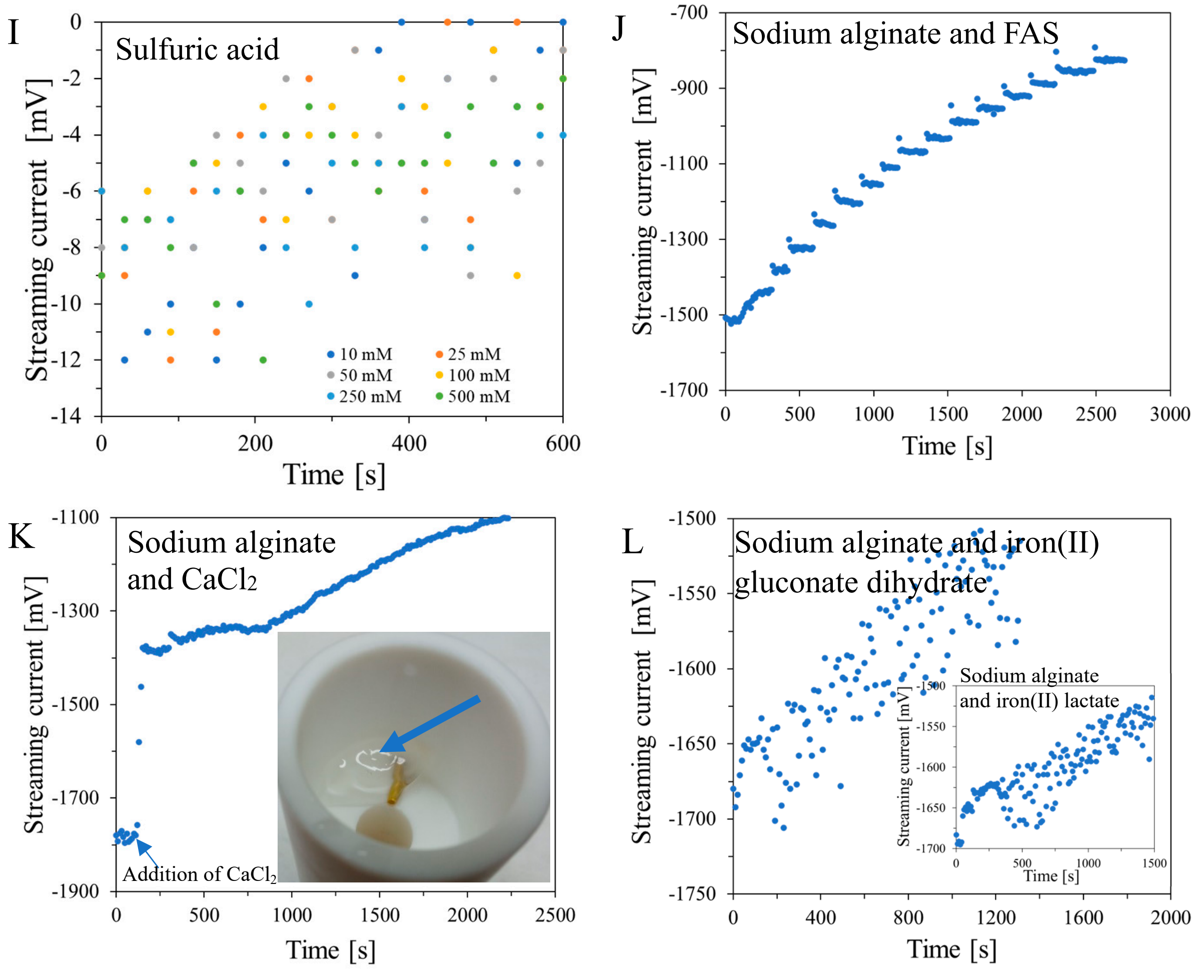

Supplementary Figure S17. Streaming current of FAS (A), XO (B), $CaCl_2$ (C), sodium alginate (D), iron(II) lactate (E), iron(II) gluconate dihydrate (F), gelatine 300 Bloom, type A (G), Pluronic F-127 (H) and sulfuric acid (I) solutions. In J–L, the graphs corresponding to the reaction of the following substrates are presented: sodium alginate and FAS (J), sodium alginate and $CaCl_2$ (K), sodium alginate and iron(II) gluconate dihydrate (L), and sodium alginate and iron(II) lactate – inset in (L). For each reaction, 15 mL of 0.25% sodium alginate was titrated periodically in 50 µL portions of: 1 mM FAS (J), 3.5% $CaCl_2$ (K), 1 mM iron(II) gluconate dihydrate (L), and 1 mM iron(II) lactate – inset in (L). Inset in (K) is a photograph of a measuring cup with calcium alginate hydrogel formed (indicated with the blue arrow).

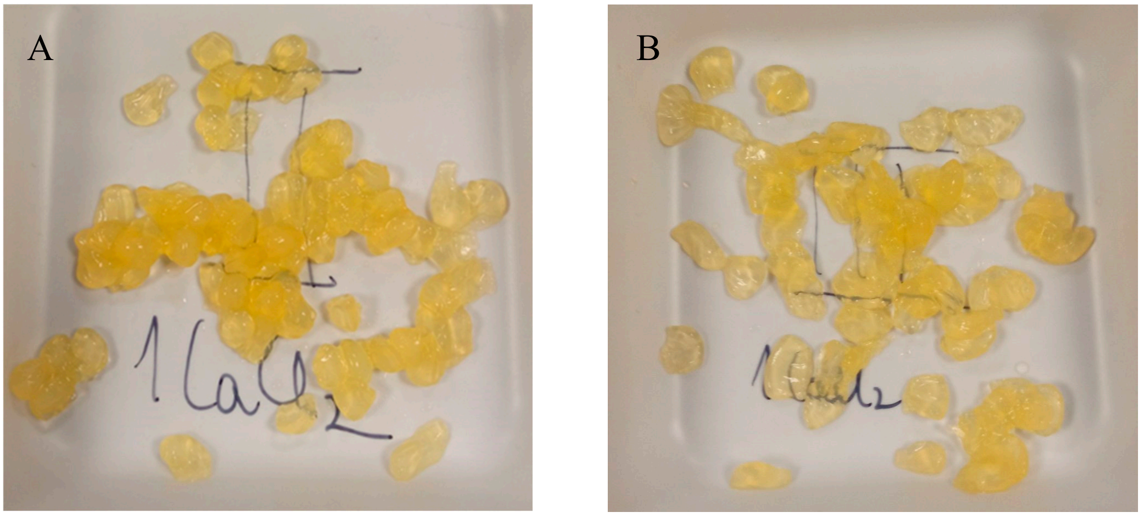

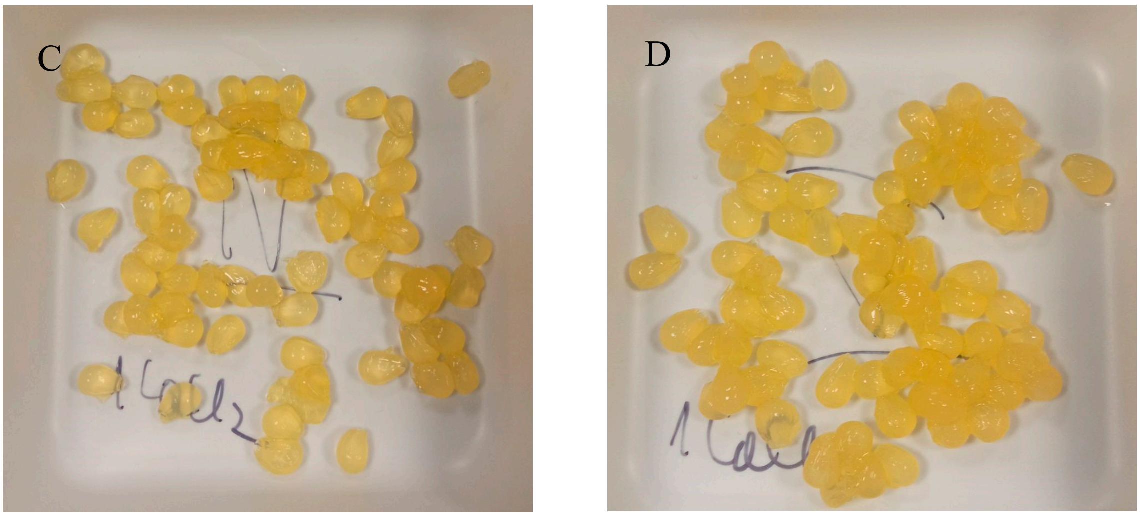

Supplementary Figure S18. Alginate capsules obtained by dropping the sodium alginate polymer solution of a low viscosity (Sigma) to the Fricke solution with 0% $CaCl_2$. The concentration of the sodium alginate solution was as follows: 2% (A), 2.3% (B), 2.7% (C) and 3% (D)

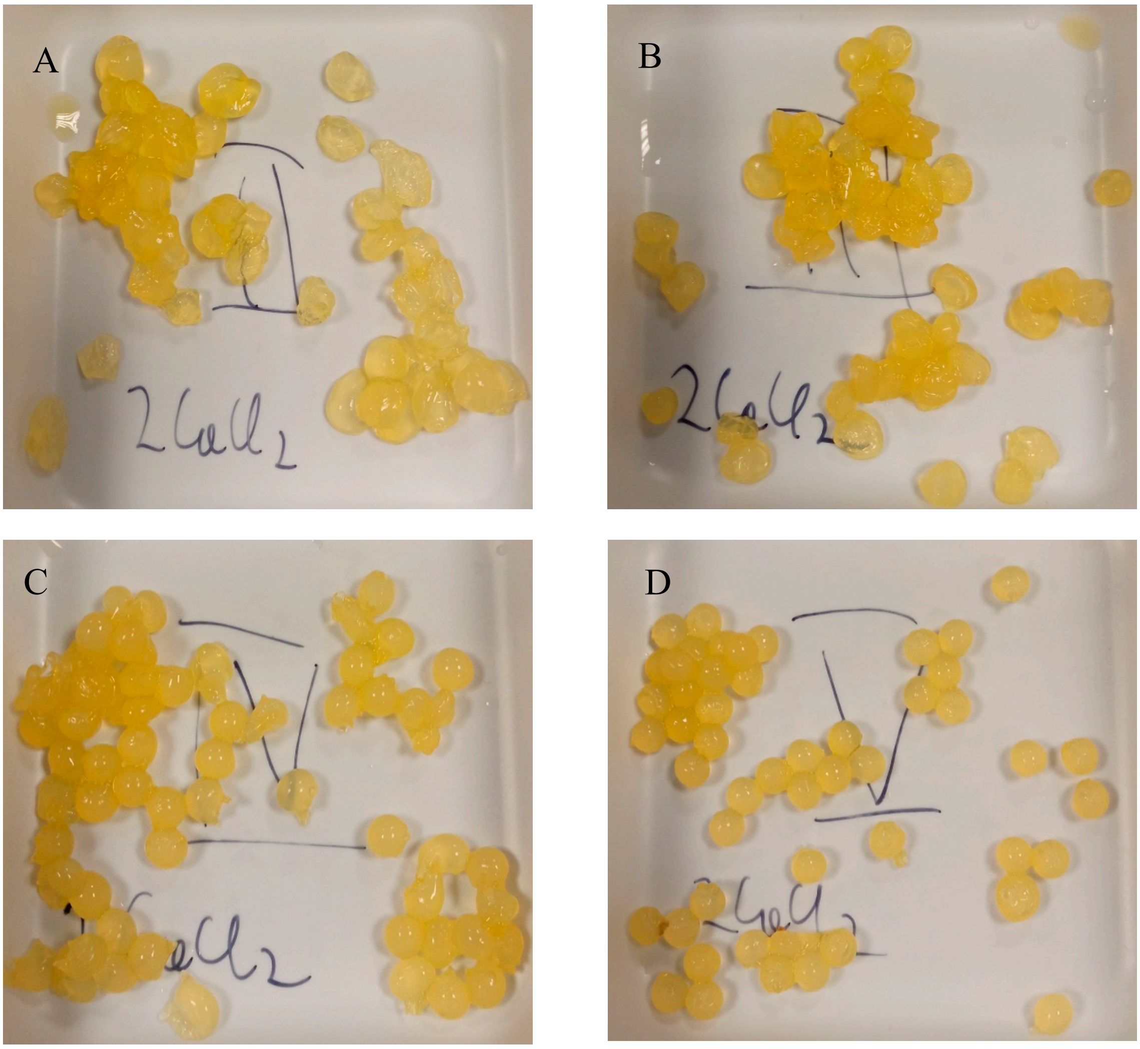

Supplementary Figure S19. Alginate capsules obtained by dropping the sodium alginate polymer solution of a low viscosity (Sigma) to the Fricke solution containing 1% $CaCl_2$. The concentration of the sodium alginate solution was as follows: 2% (A), 2.3% (B), 2.7% (C) and 3% (D).

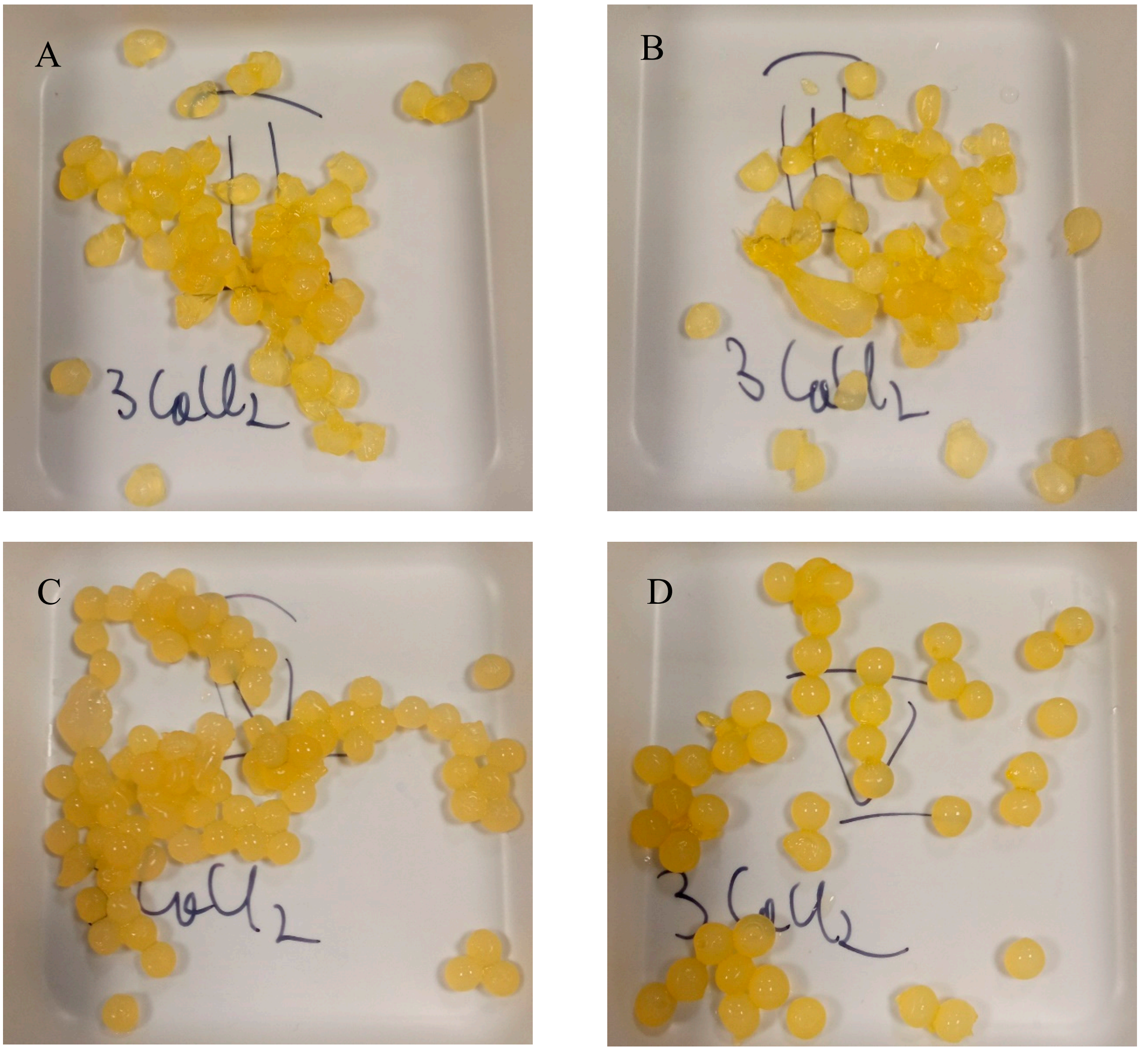

Supplementary Figure S20. Alginate capsules obtained by dropping the sodium alginate polymer solution of a low viscosity (Sigma) to the Fricke solution containing 1.5% $CaCl_2$. The concentration of the sodium alginate solution was as follows: 2% (A), 2.3% (B), 2.7% (C) and 3% (D).

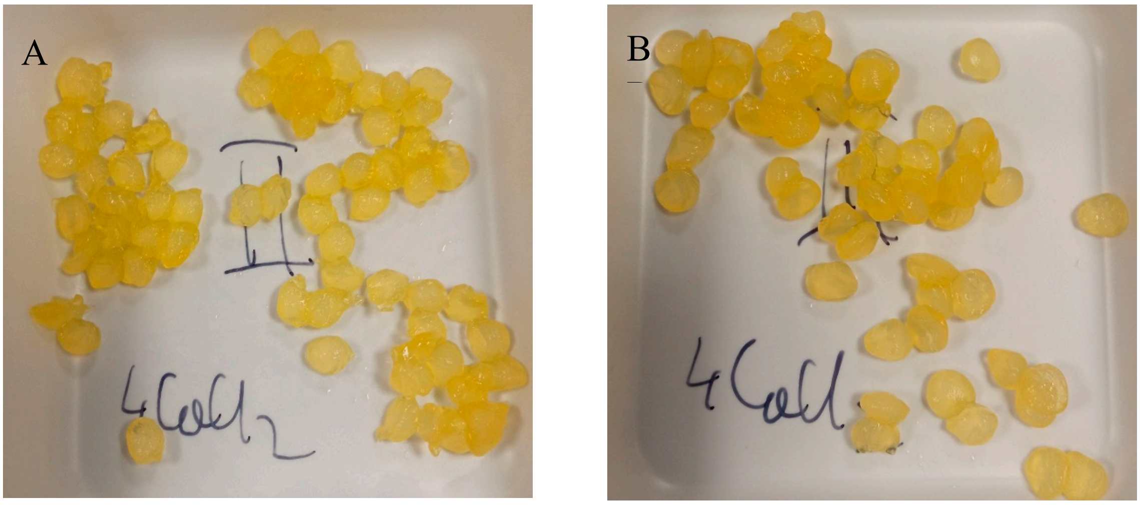

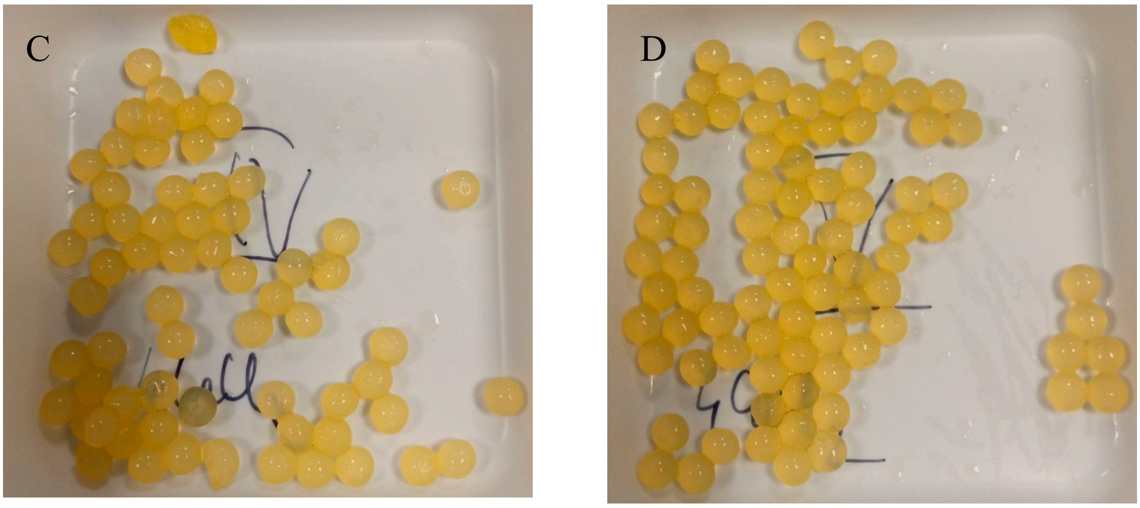

Supplementary Figure S21. Alginate capsules obtained by dropping the sodium alginate polymer solution of a low viscosity (Sigma) to the Fricke solution containing 2.5% $CaCl_2$. The concentration of the sodium alginate solution was as follows: 2% (A), 2.3% (B), 2.7% (C) and 3% (D).

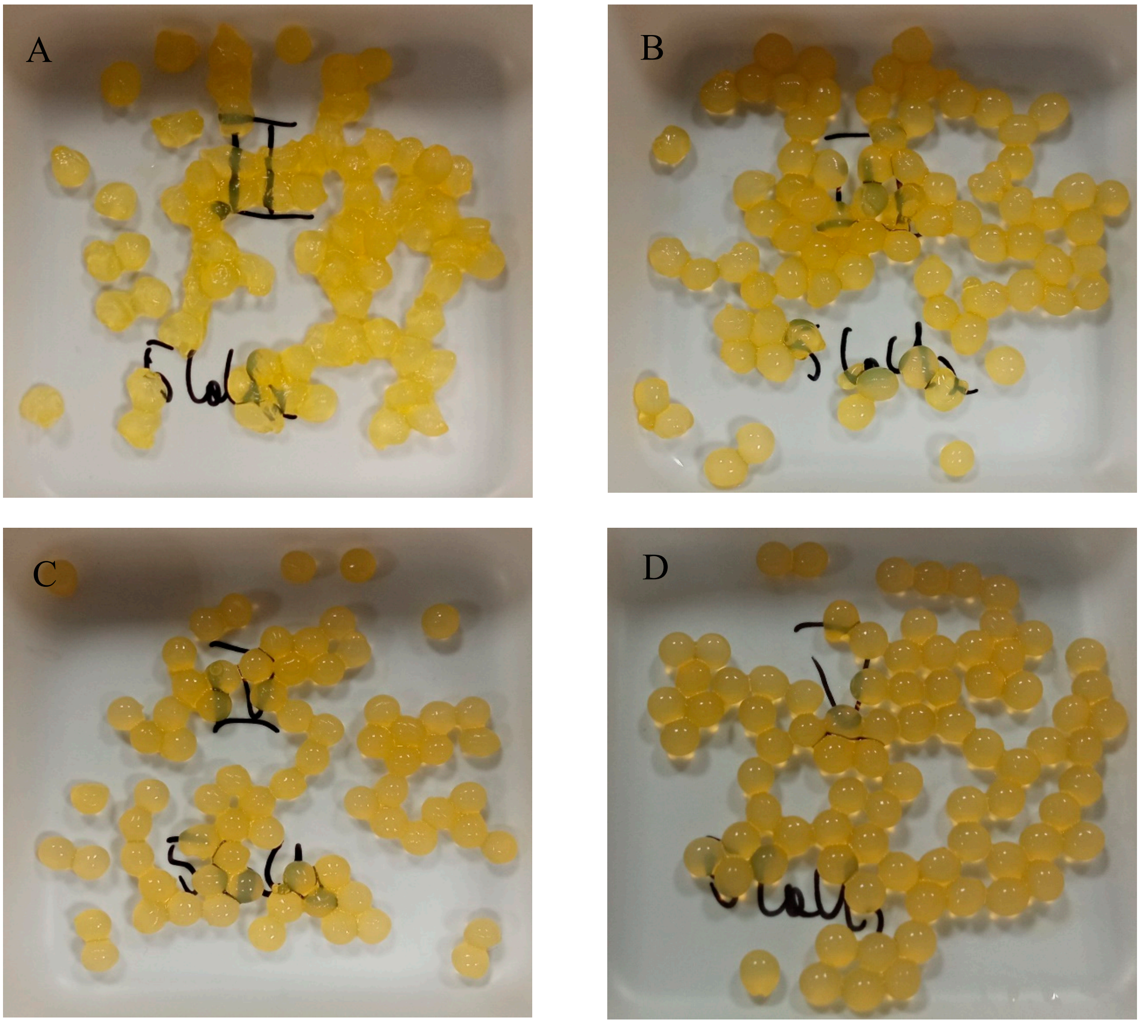

Supplementary Figure S22. Alginate capsules obtained by dropping the sodium alginate polymer solution of a low viscosity (Sigma) to the Fricke solution containing 3.5% $CaCl_2$. The concentration of the sodium alginate solution was as follows: 2% (A), 2.3% (B), 2.7% (C) and 3% (D).

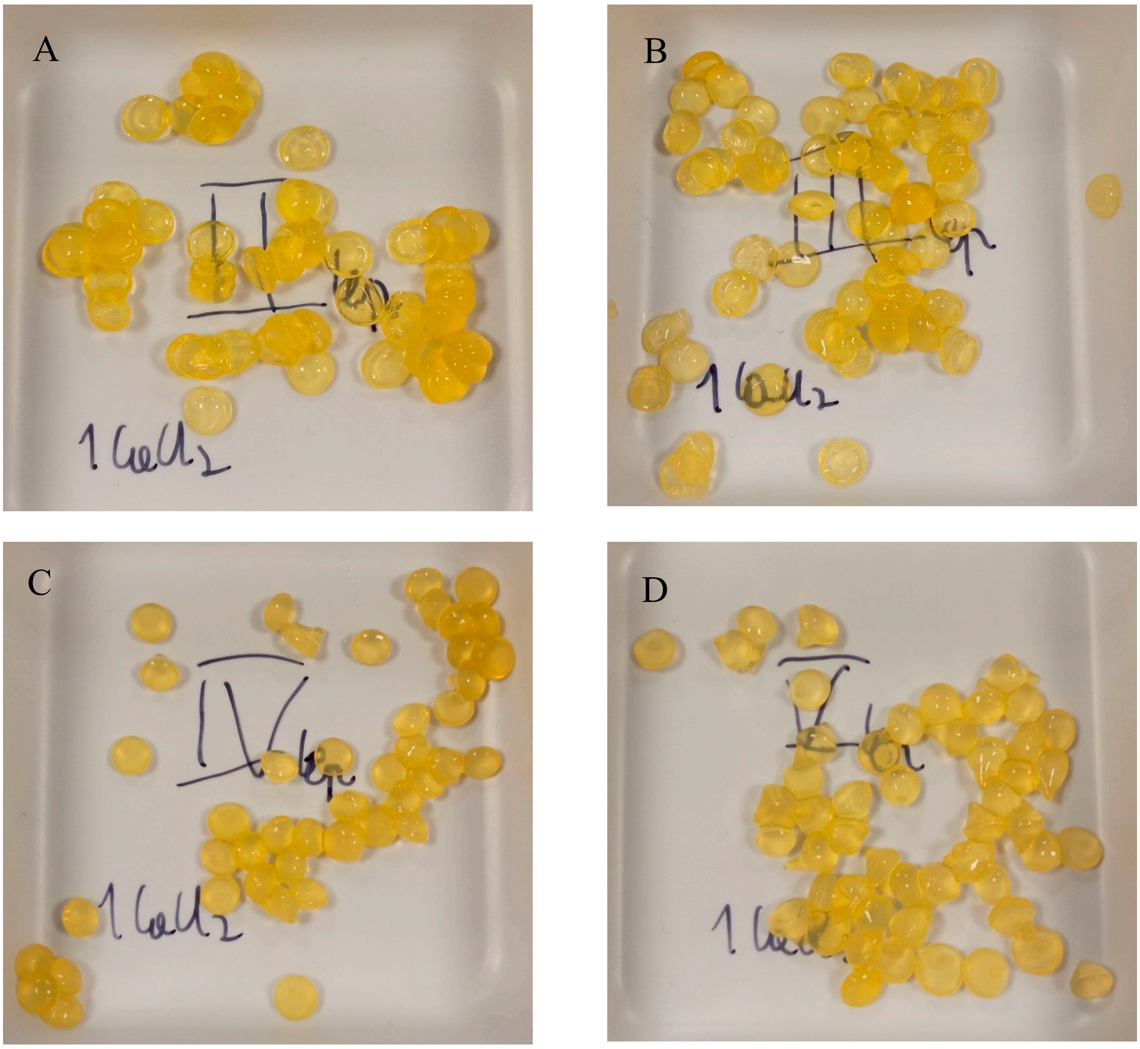

Supplementary Figure S23. Alginate capsules obtained by dropping the sodium alginate polymer solution of a medium viscosity (Sigma) to the Fricke solution containing 0% $CaCl_2$. The concentration of the sodium alginate solution was as follows: 2% (A), 2.3% (B), 2.7% (C) and 3% (D).

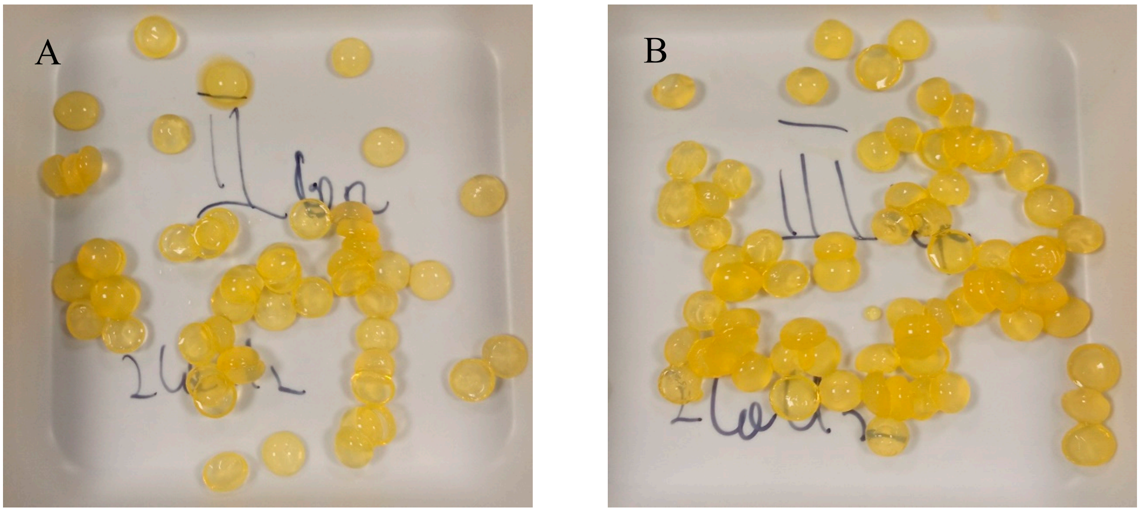

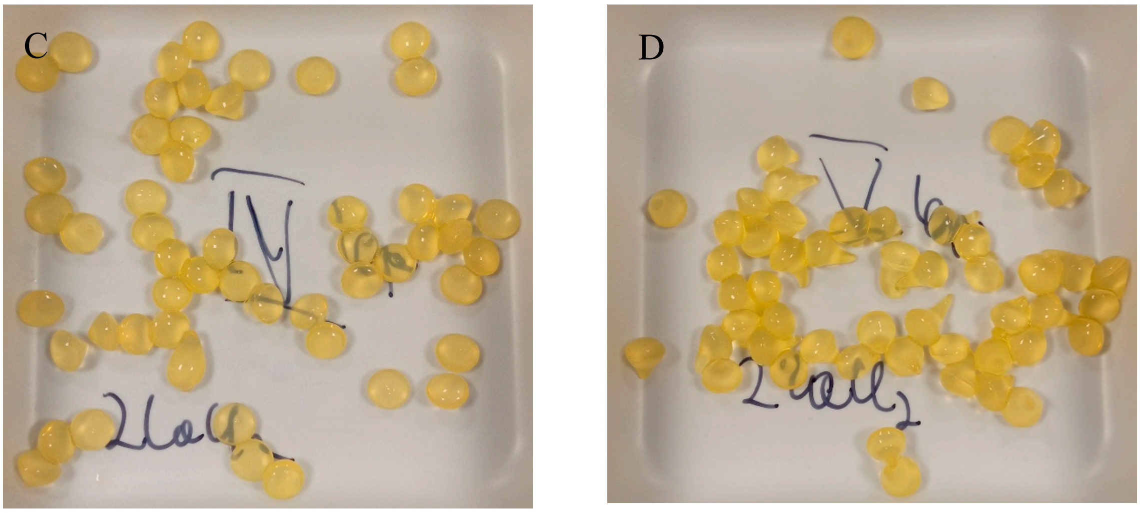

Supplementary Figure S24. Alginate capsules obtained by dropping the sodium alginate polymer solution of a medium viscosity (Sigma) to the Fricke solution containing 1% $CaCl_2$. The concentration of the sodium alginate solution was as follows: 2% (A), 2.3% (B), 2.7% (C) and 3% (D).

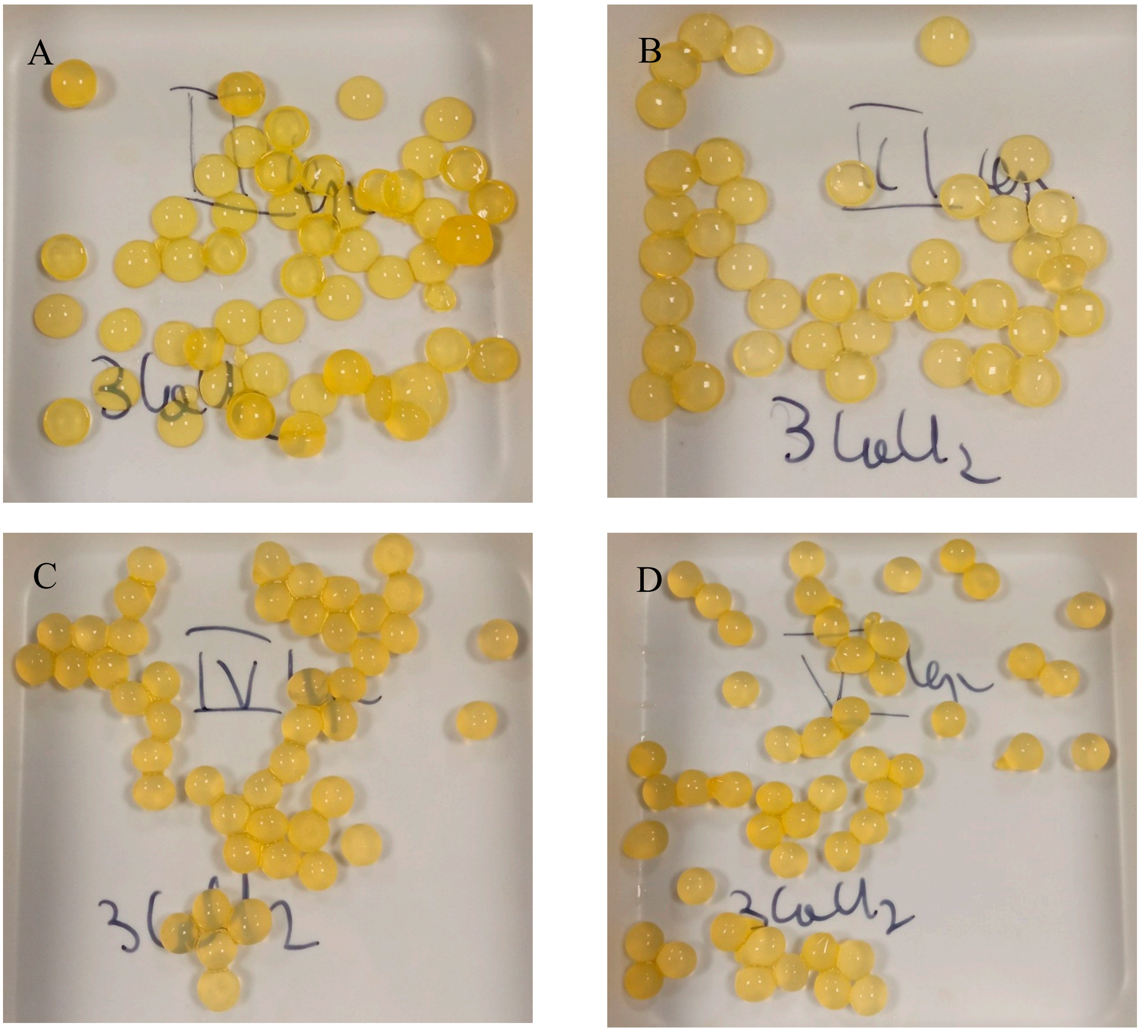

Supplementary Figure S25. Alginate capsules obtained by dropping the sodium alginate polymer solution of a medium viscosity (Sigma) to the Fricke solution containing 1.5% $CaCl_2$. The concentration of the sodium alginate solution was as follows: 2% (A), 2.3% (B), 2.7% (C) and 3% (D).

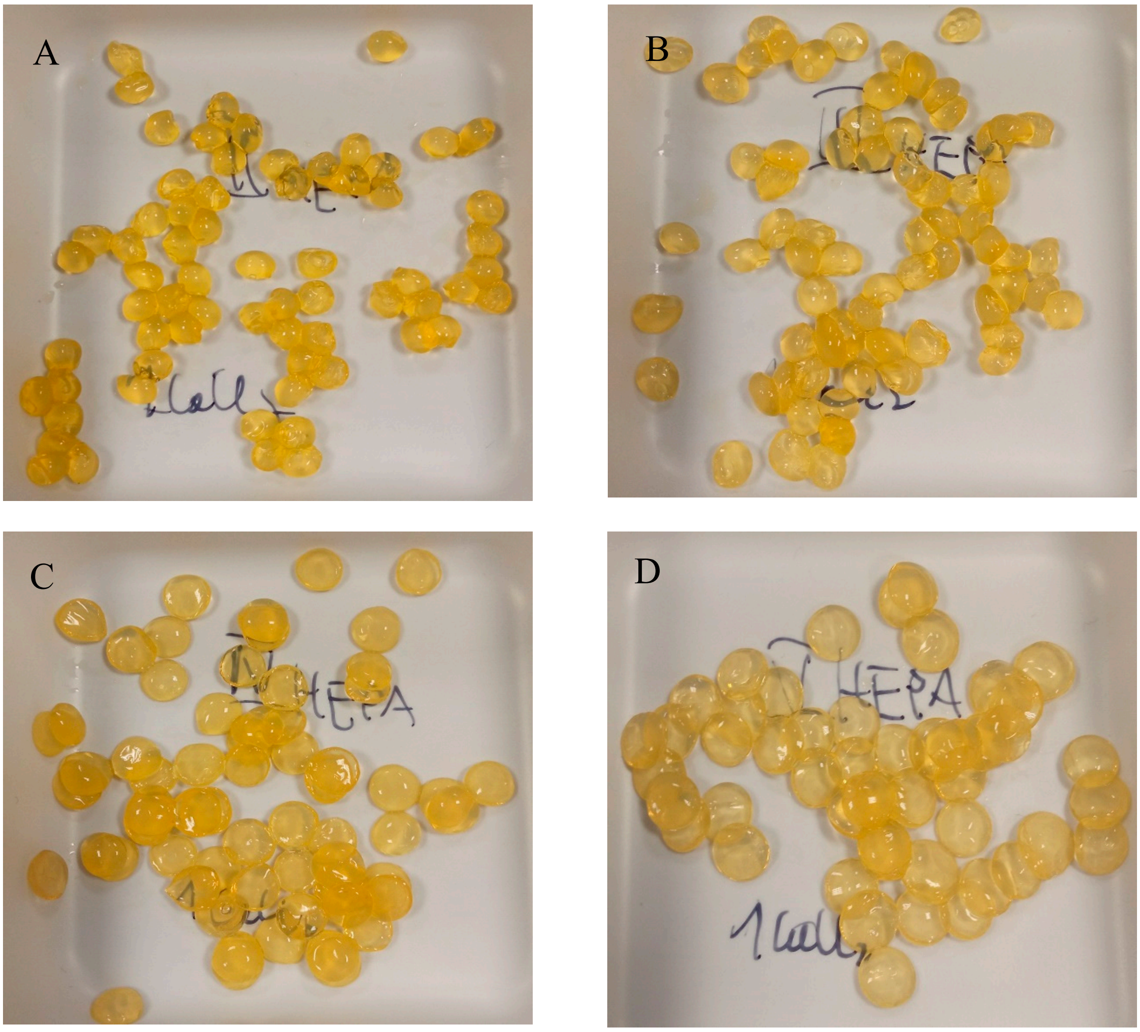

Supplementary Figure S26. Alginate capsules obtained by dropping the sodium alginate polymer solution (Heppe) to the Fricke solution containing 0% $CaCl_2$. The concentration of the sodium alginate solution was as follows: 2% (A), 2.3% (B), 2.7% (C) and 3% (D).

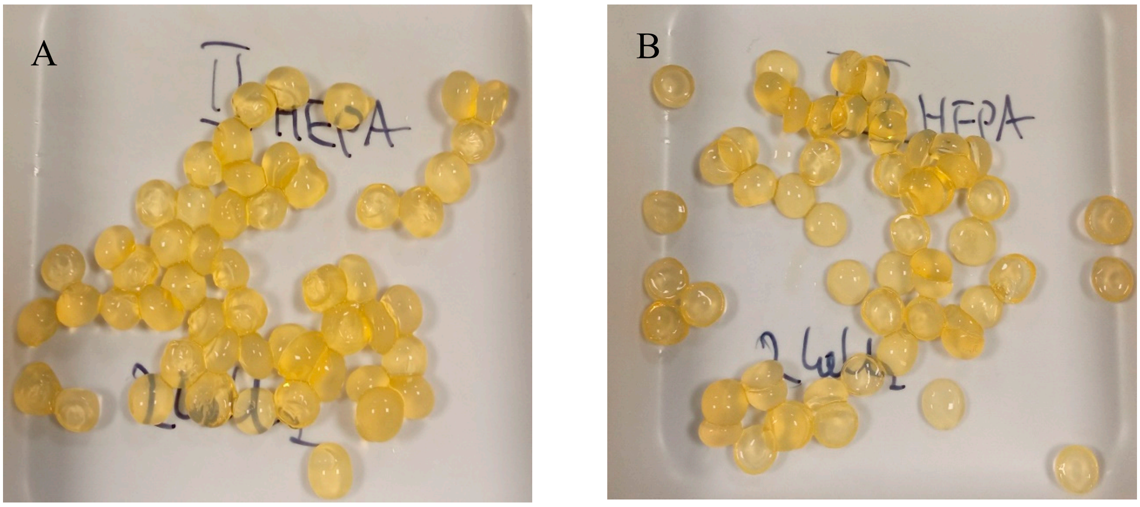

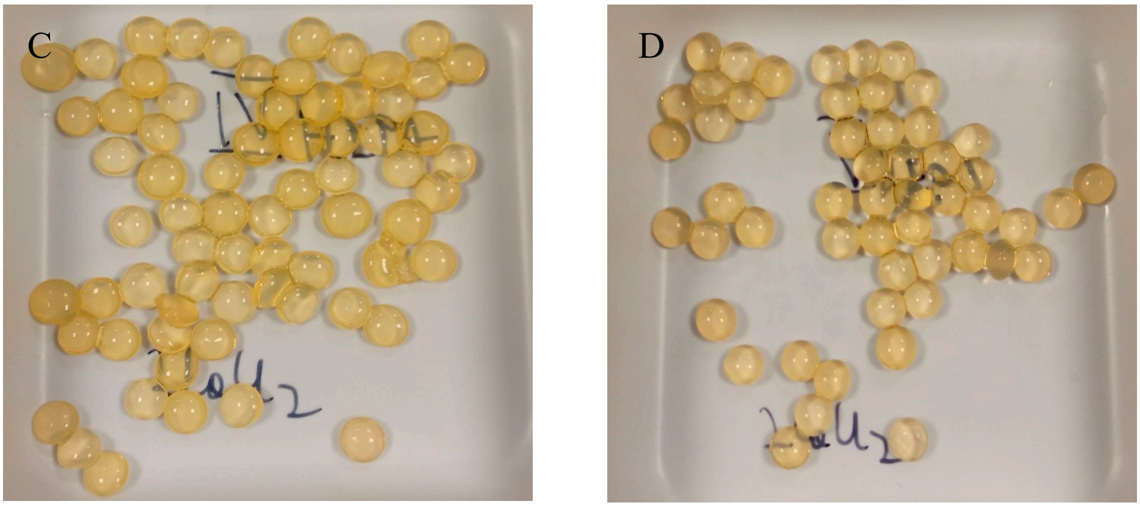

Supplementary Figure S27. Alginate capsules obtained by dropping the sodium alginate polymer solution (Heppe) to the Fricke solution containing 1% $CaCl_2$. The concentration of the sodium alginate solution was as follows: 2% (A), 2.3% (B), 2.7% (C) and 3% (D).

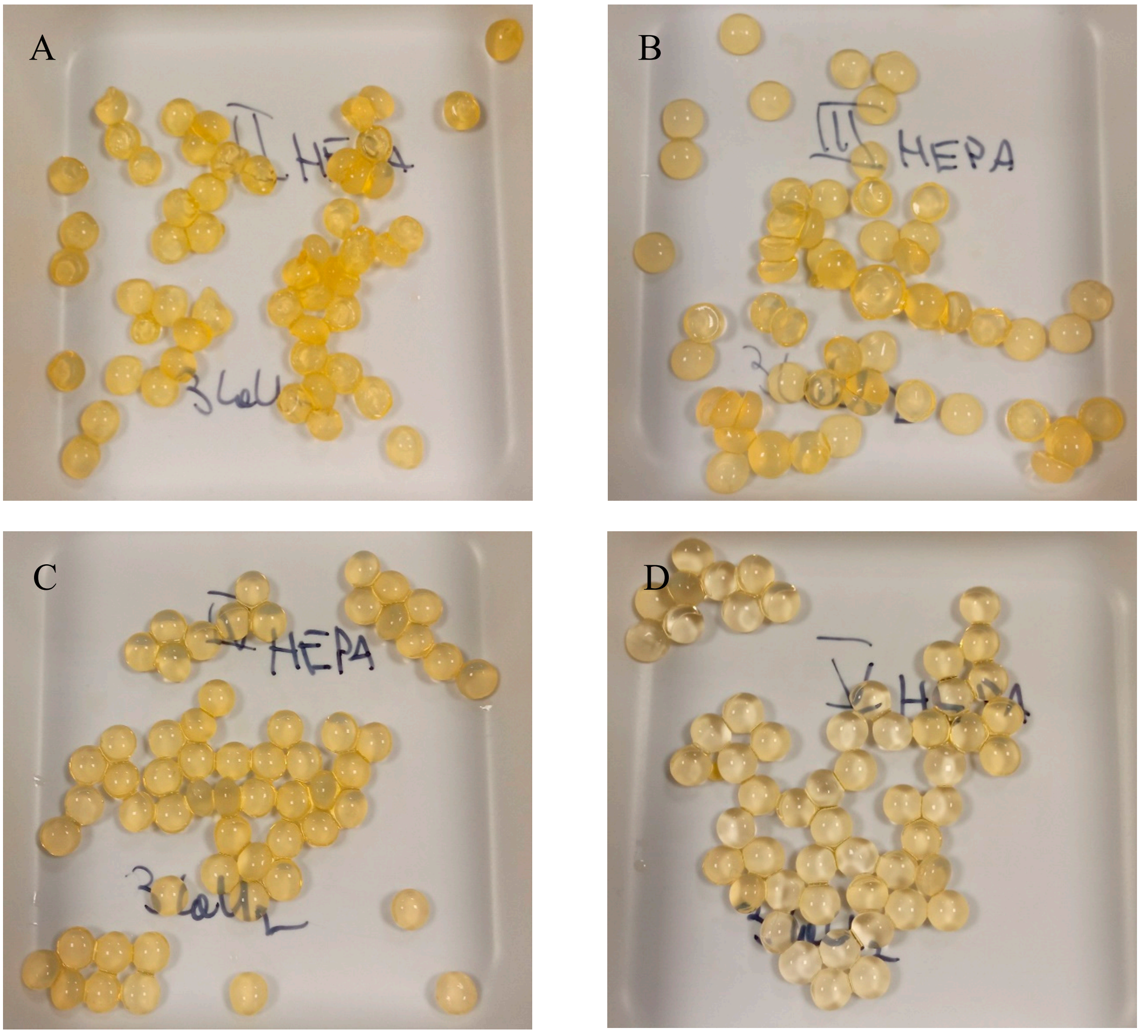

Supplementary Figure S28. Alginate capsules obtained by dropping the sodium alginate polymer solution (Heppe) to the Fricke solution containing 1.5% $CaCl_2$. The concentration of the sodium alginate solution was as follows: 2% (A), 2.3% (B), 2.7% (C) and 3% (D).

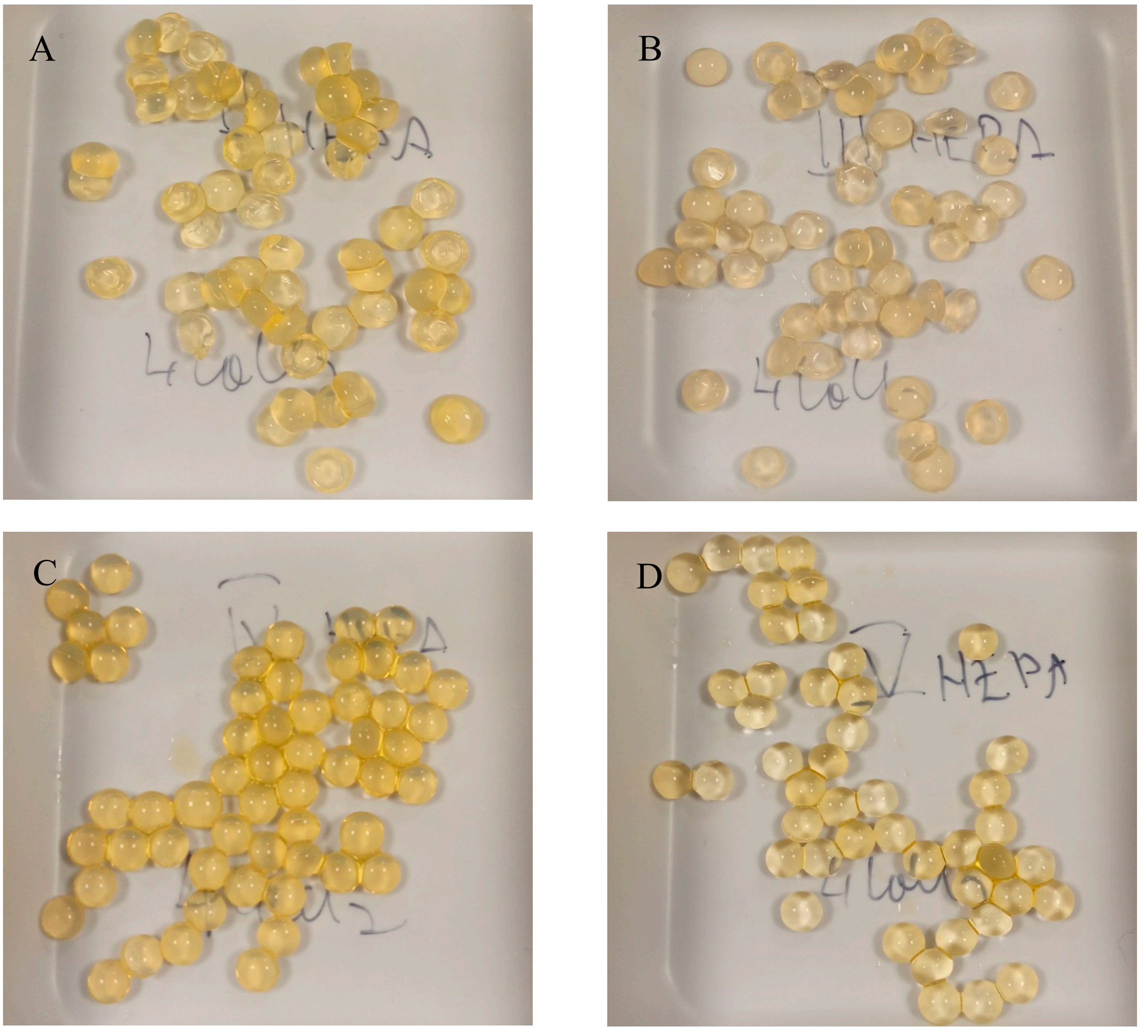

Supplementary Figure S29. Alginate capsules obtained by dropping the sodium alginate polymer solution (Heppe) to the Fricke solution containing 2.5% $CaCl_2$. The concentration of the sodium alginate solution was as follows: 2% (A), 2.3% (B), 2.7% (C) and 3% (D).

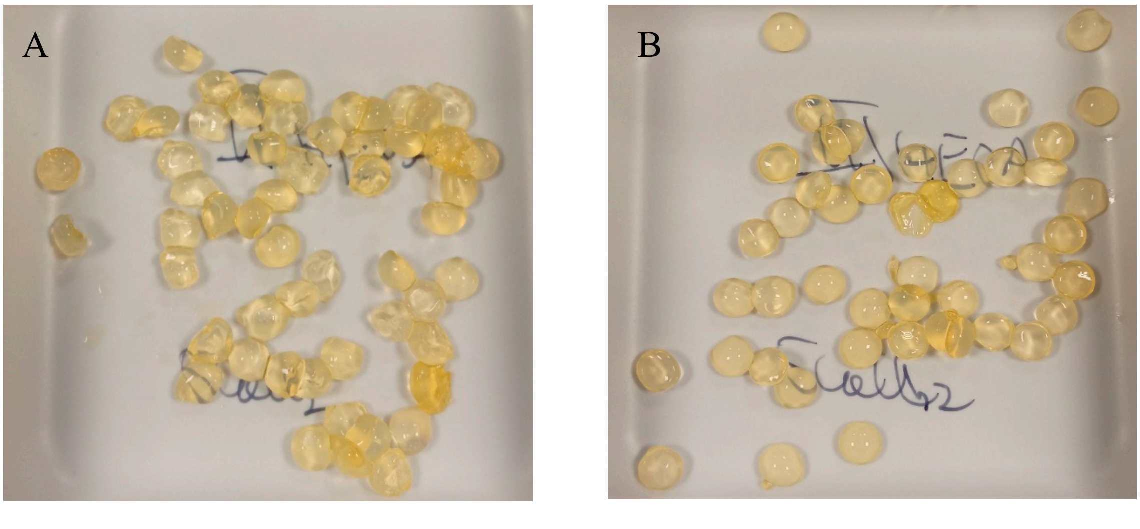

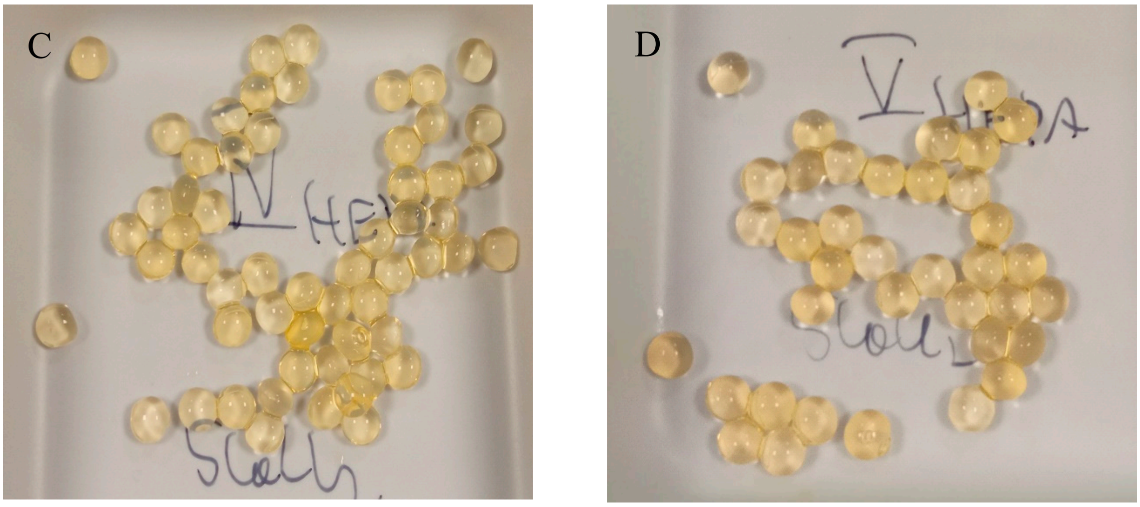

Supplementary Figure S30. Alginate capsules obtained by dropping the sodium alginate polymer solution (Heppe) to the Fricke solution containing 3.5% $CaCl_2$. The concentration of the sodium alginate solution was as follows: 2% (A), 2.3% (B), 2.7% (C) and 3% (D).

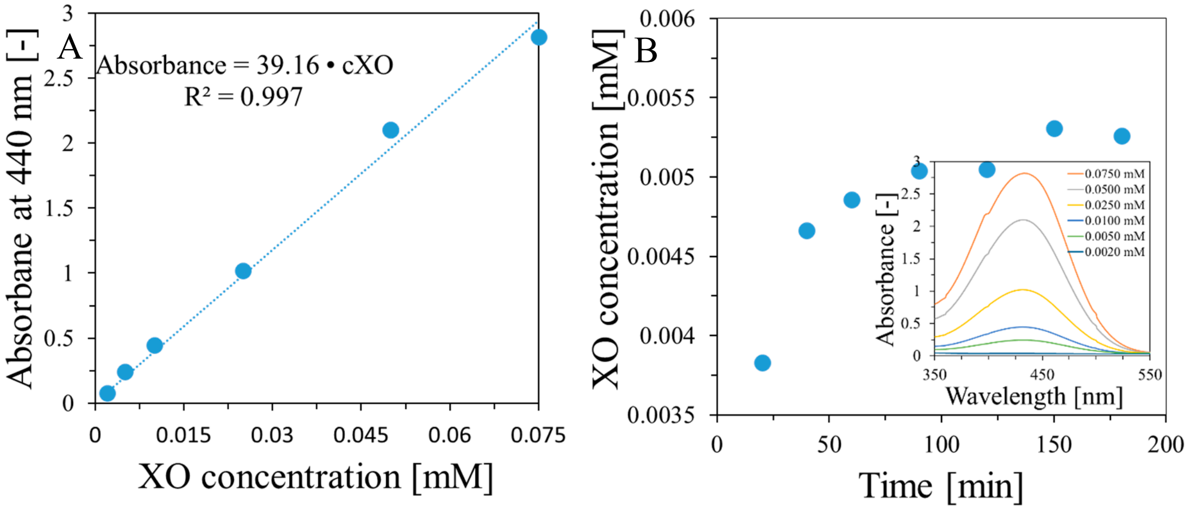

Supplementary Figure S31. Diffusion of XO from alginate capsules prepared by reaction of sodium alginate (3.5%) with Fricke solution (FAS: 1 mM, H_2SO_4: 50 mM, XO: 0.165 mM) (B). The capsules were immersed in re-distilled water. A is for the calibration curve that was obtained after registering the absorbance spectra (inset in B).

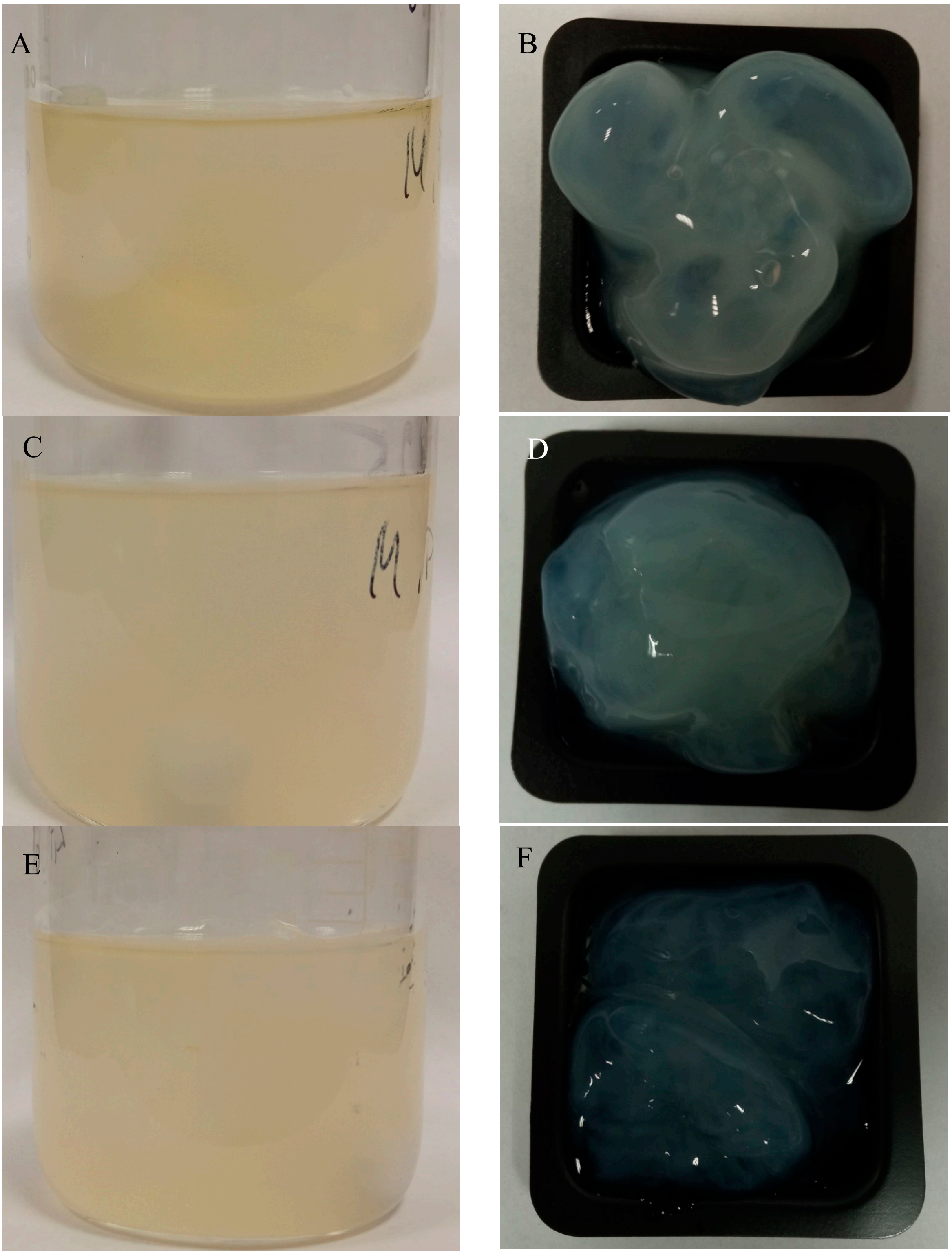

Supplementary Figure S32. Macro-gels prepared through the reaction of sodium alginate with the Fricke solution (sodium alginate with FAS and then H_2SO_4) for the following concentrations of the sodium alginate solution: 1.75% (A,B), 1.5% (C,D), 1.35% (E,F).

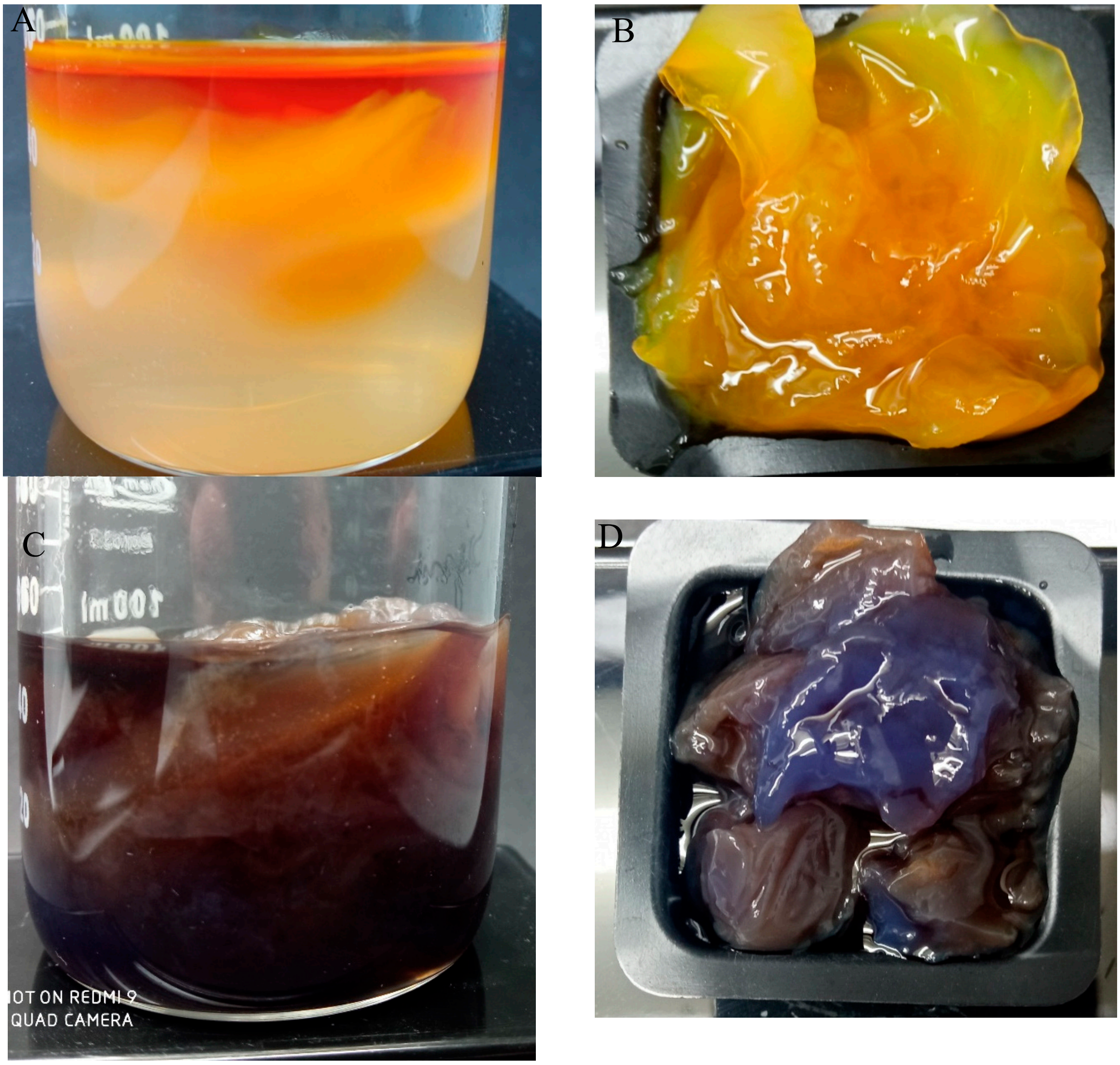

Supplementary Figure S33. Macro-gels prepared through the reaction of sodium alginate with the Fricke solution (FAS: 1mM, H_2SO_4: 50mM and XO: 0.165 mM added together to a sodium alginate solution stirred at about 400 rpm) for 1.75% sodium alginate solution in a beaker (A) and after removal from a beaker (B), on a plastic container. In C and D the photographs of the corresponding macro-gels are shown after irradiation (30 Gy).

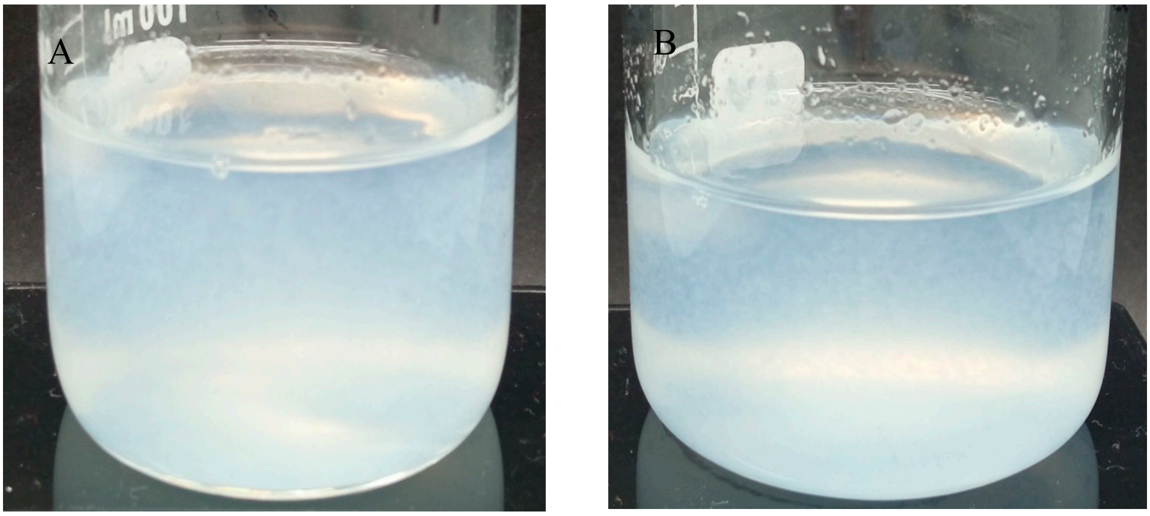

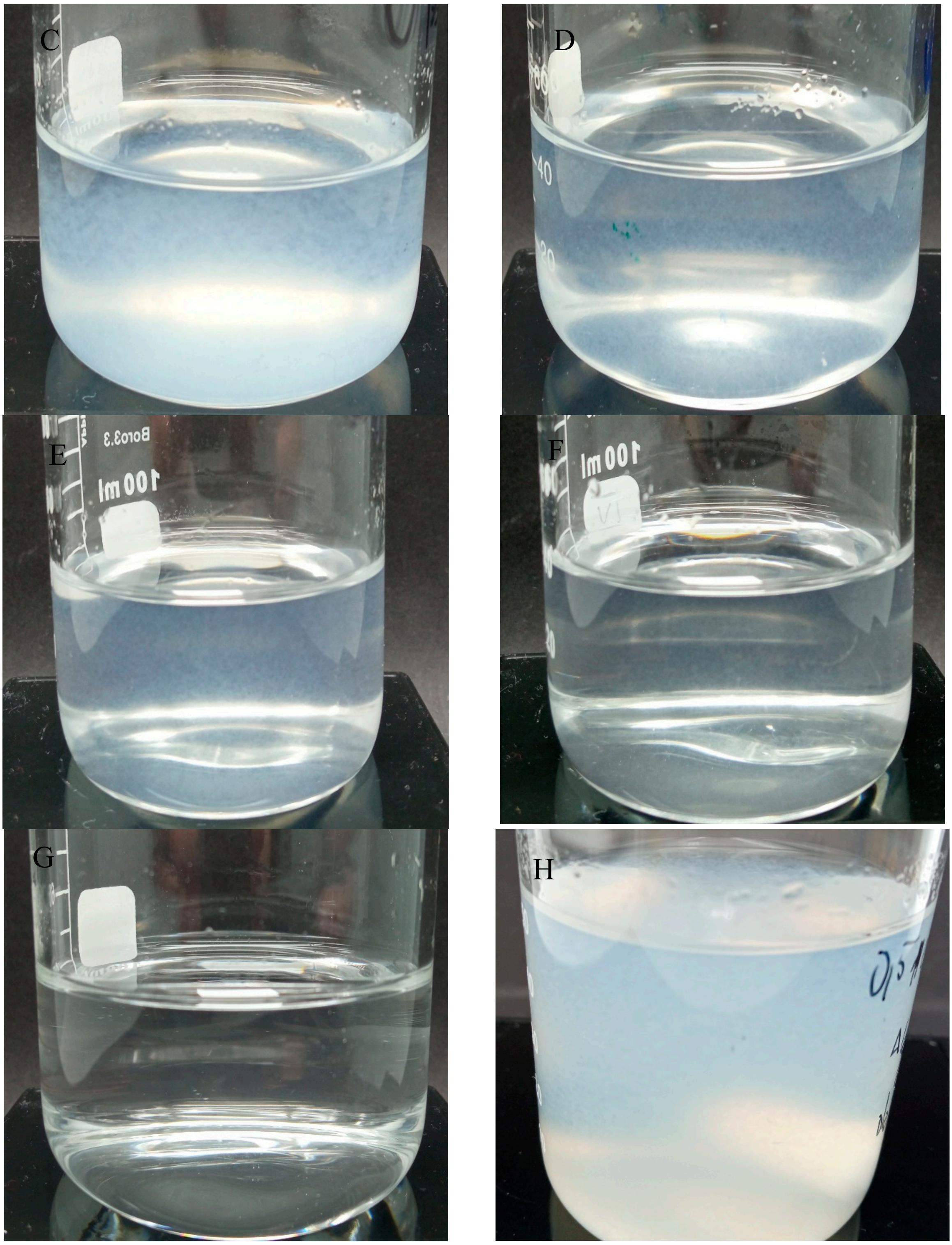

Supplementary Figure S34. Nano- and micro-gels as prepared through the reaction of sodium alginate with the Fricke solution (FAS and sulfuric acid added to sodium alginate solution), for sodium alginate concentrations: 0.65% (A), 0.5% (B), 0.25% (C), 0.15% (D), 0.1% (E), 0.05% (F), 0.015% (G), and 0.5% without FAS added (H).

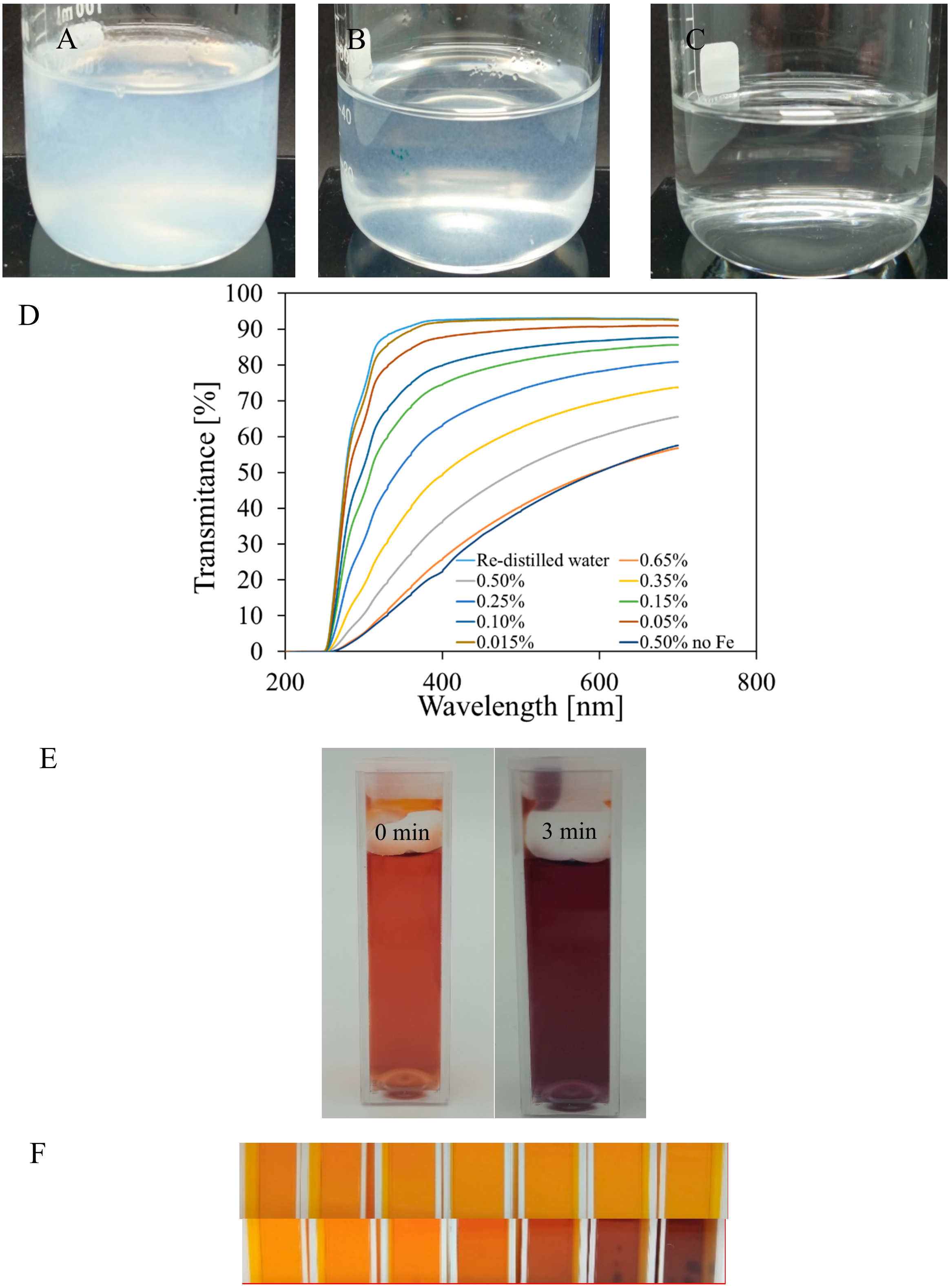

Supplementary Figure S35. Nano- and micro-gels as prepared through the reaction of sodium alginate with the Fricke solution (FAS added to sodium alginate solution following by addition of sulfuric acid), for sodium alginate concentrations: 0.65% (A), 0.15% (B) and 0.015% (C). In D, the transmittance spectra for the micro-gel solution made of 0.65–0.015% alginate solutions and re-distilled water are shown. In E photographs of a solution of 0.5% sodium alginate and 1 mM FAS, without sulfuric acid, taken immediately and 3 minutes after the addition of 0.1 mM XO are presented. In F, the photographs are presented for micro-gels in Pluronic F-127 matrix before (first line) and after irradiation (second line) for the doses of 0, 1, 2, 5, 10, 15, and 20 Gy.

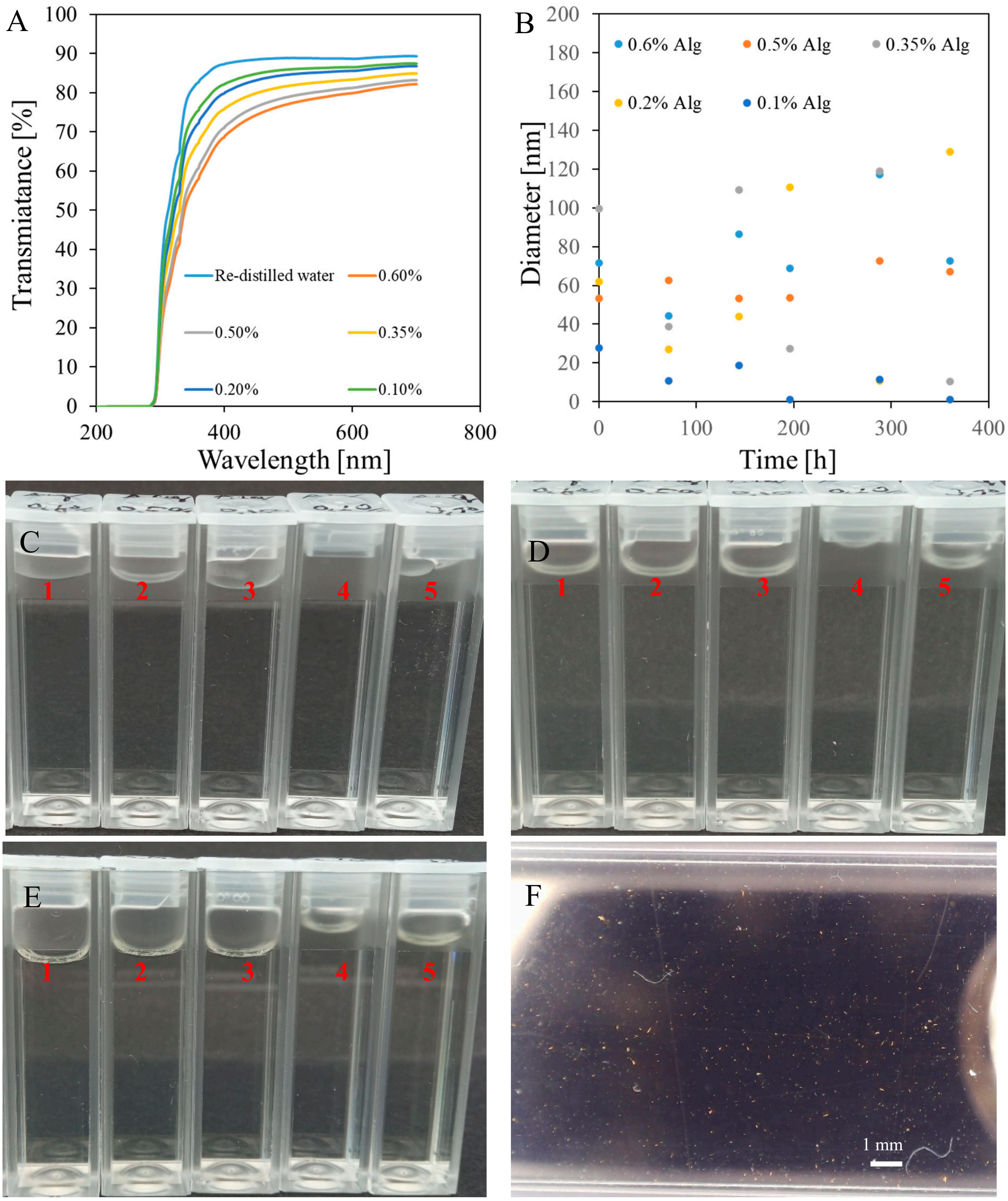

Supplementary Figure S36. **A**: transmittance spectra of solutions of 0.1-0.6% sodium alginate Heppe, 1 mM FAS and 1-3 mM H_2SO_4 (pH of solutions in the range of 3.9-4.2). **B**: Hydrodynamic diameters of microstructures present in the tested solutions over time. **C**, **D** and **E**: photos of cuvettes with tested solutions taken immediately after preparation (**C**) and 7 (**D**) and 14 (**E**) days after preparation, respectively. The numbers 1 to 5 indicate the following concentrations of sodium alginate in solution: 0.6% (1), 0.5% (2), 0.35% (3), 0.2% (4), 0.1% (5). **F**: microscopic photo of 0.5% sodium alginate solution taken 17 days after preparation of the solution.

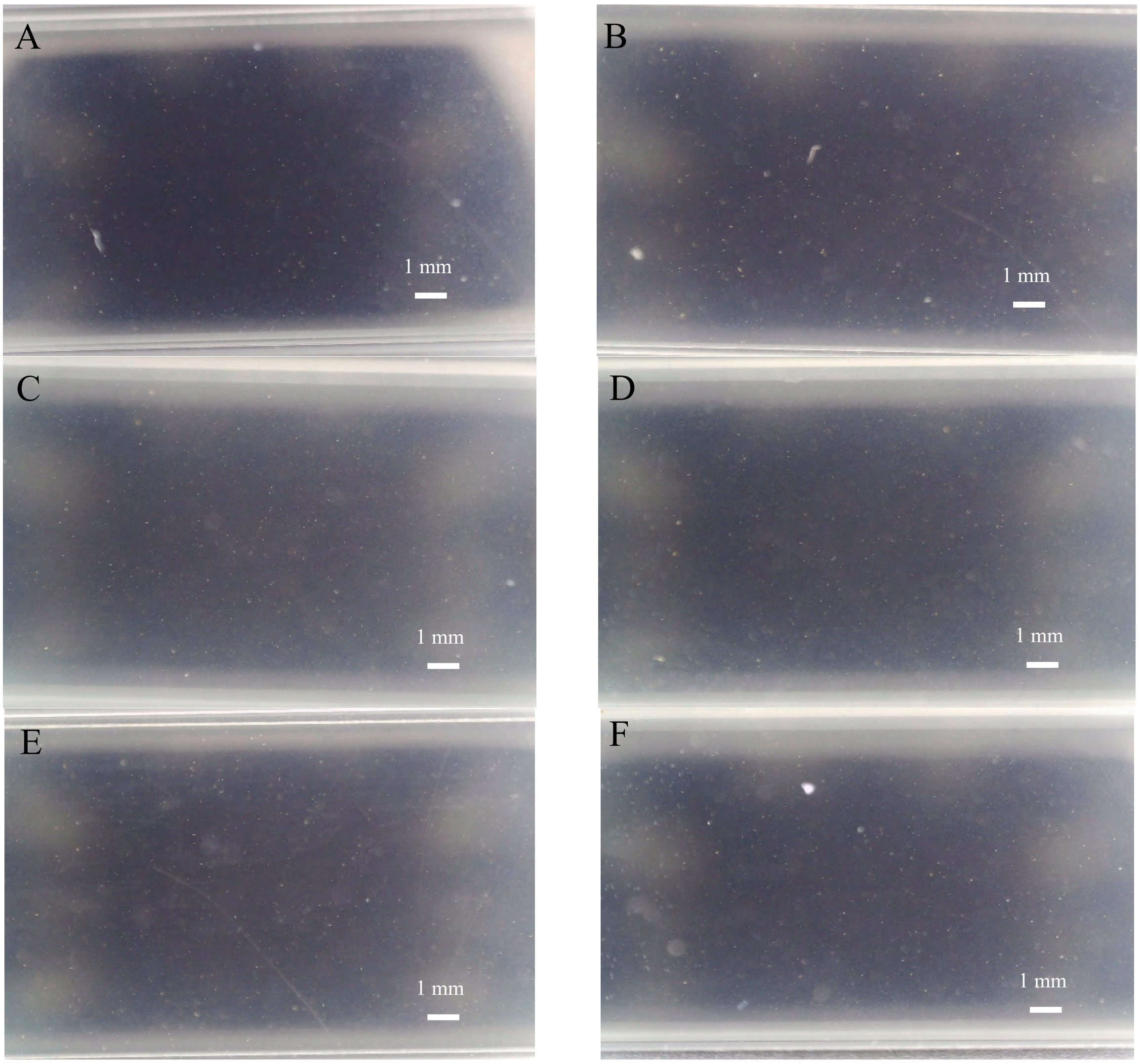

Supplementary Figure S37 Microscopic photographs of solutions of 0.5% Sigma low-viscosity sodium alginate, 2 mM FAS and 0 mM H_2SO4 (pH=5.7) one hour after irradiation with dose **A**: 0 Gy, **B**: 3 Gy, **C**: 7 Gy, **D**: 10 Gy, **E**: 20 Gy, **F**: 30 Gy. The solutions were prepared one hour before irradiation.

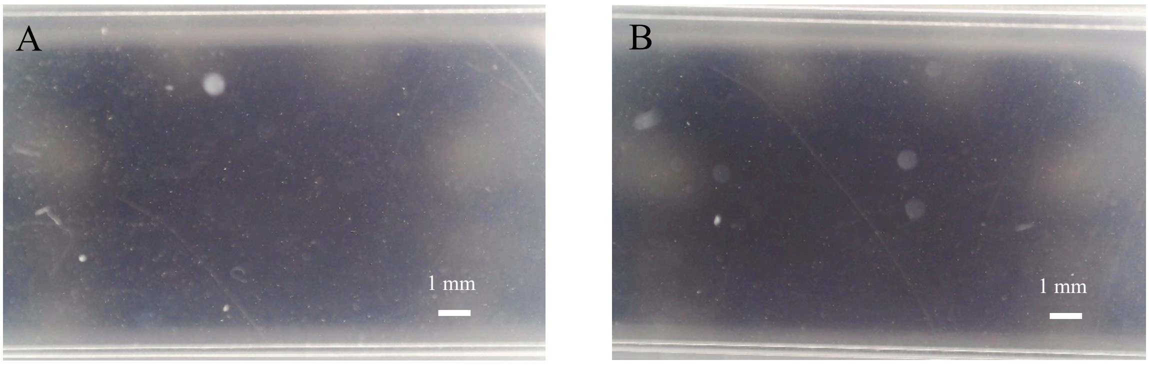

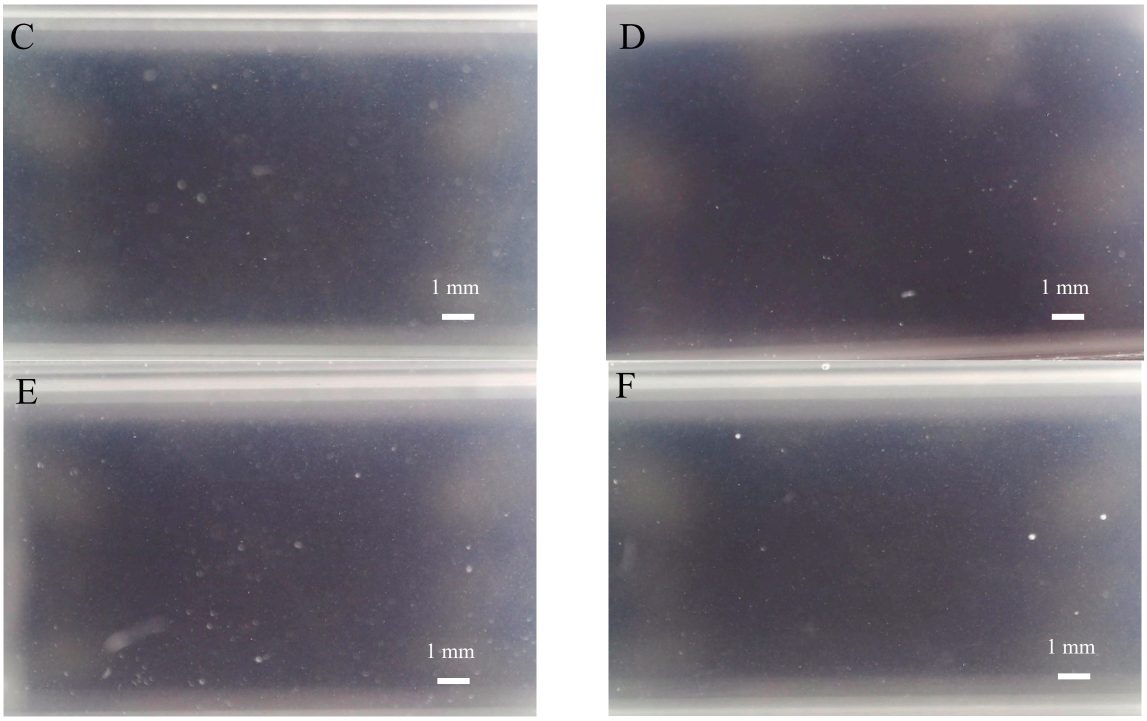

Supplementary Figure S38 Microscopic photographs of solutions of 0.5% Sigma low-viscosity sodium alginate, 2 mM FAS and 2 mM H_2SO4 (pH=3.9) one hour after irradiation with dose **A**: 0 Gy, **B**: 3 Gy, **C**: 7 Gy, **D**: 10 Gy, **E**: 20 Gy, **F**: 30 Gy. The solutions were prepared one hour before irradiation.

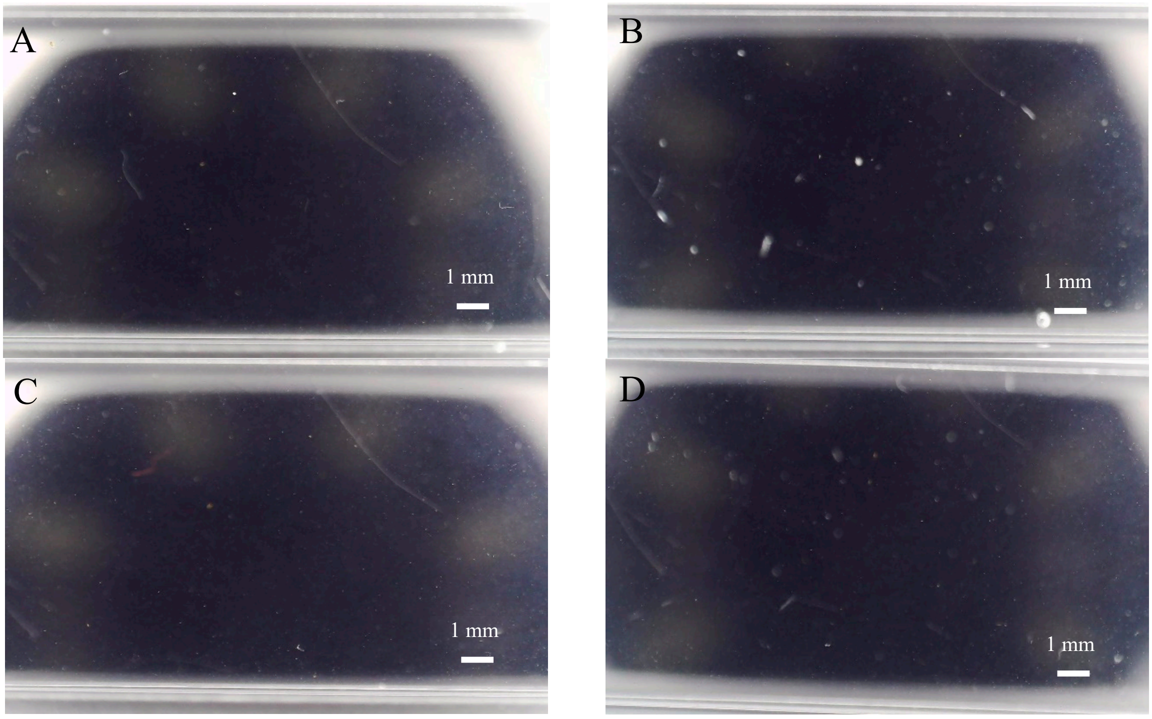

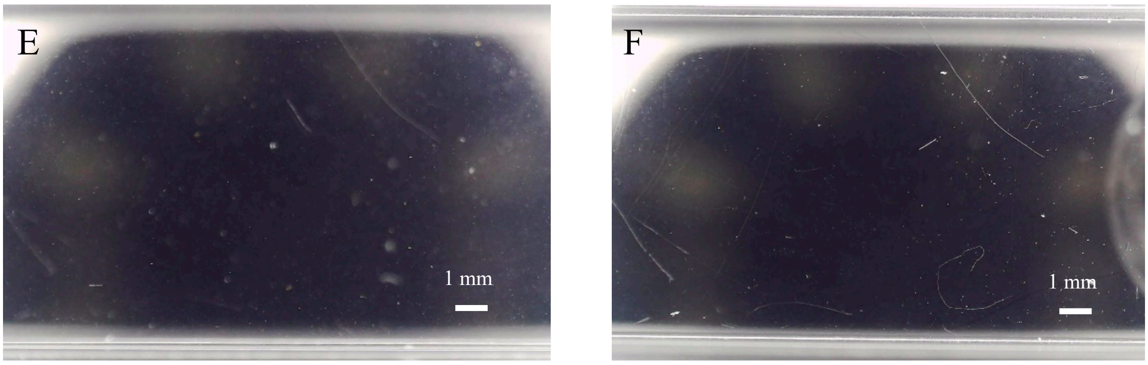

Supplementary Figure S39 Microscopic photographs of solutions of 0.5% Heppe sodium alginate, 2 mM FAS and 0 mM H_2SO4 (pH=5.7) one hour after irradiation with dose **A**: 0 Gy, **B**: 3 Gy, **C**: 7 Gy, **D**: 10 Gy, **E**: 20 Gy, **F**: 30 Gy. The solutions were prepared one hour before irradiation.

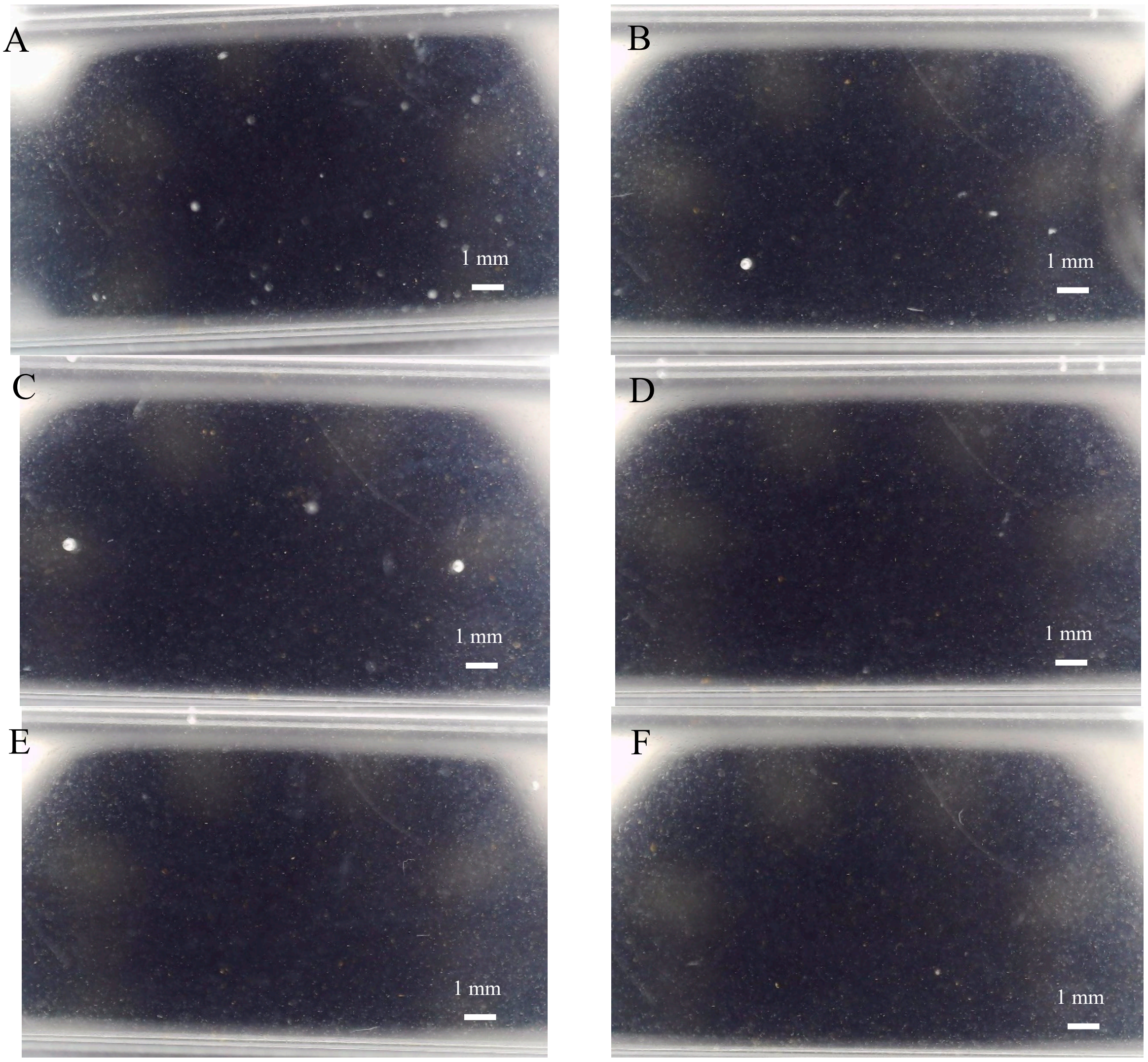

Supplementary Figure S40 Microscopic photographs of solutions of 0.5% Heppe sodium alginate, 2 mM FAS and 2 mM H_2SO4 (pH=3.9) one hour after irradiation with dose **A**: 0 Gy, **B**: 3 Gy, **C**: 7 Gy, **D**: 10 Gy, **E**: 20 Gy, **F**: 30 Gy. The solutions were prepared one hour before irradiation.

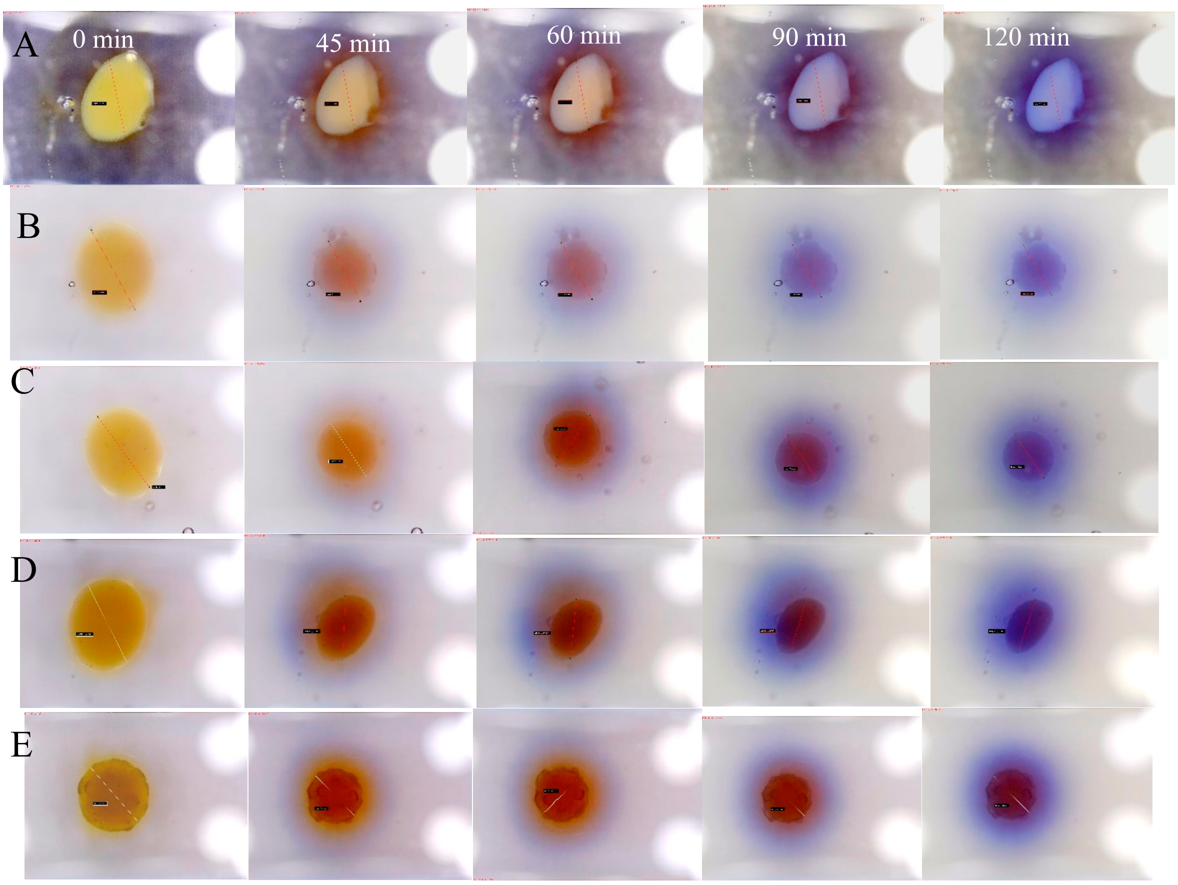

Supplementary Figure S41. Performance of alginate capsules containing Fricke dosimeter embedded in Pluronic F127 matrix stored for 0–120 min. Capsules formed in reaction of 3.5% sodium alginate with Fricke solution: 1 mM.FAS, 50 mM.H_2SO_4, 0.165 mM XO containing 1–3.5% $CaCl_2$. Concentration of $CaCl_2$: 0 (A), 1 (B), 1.5 (C), 2.5 (D), and 3.5% (E) (storage temperature: ~23 °C).

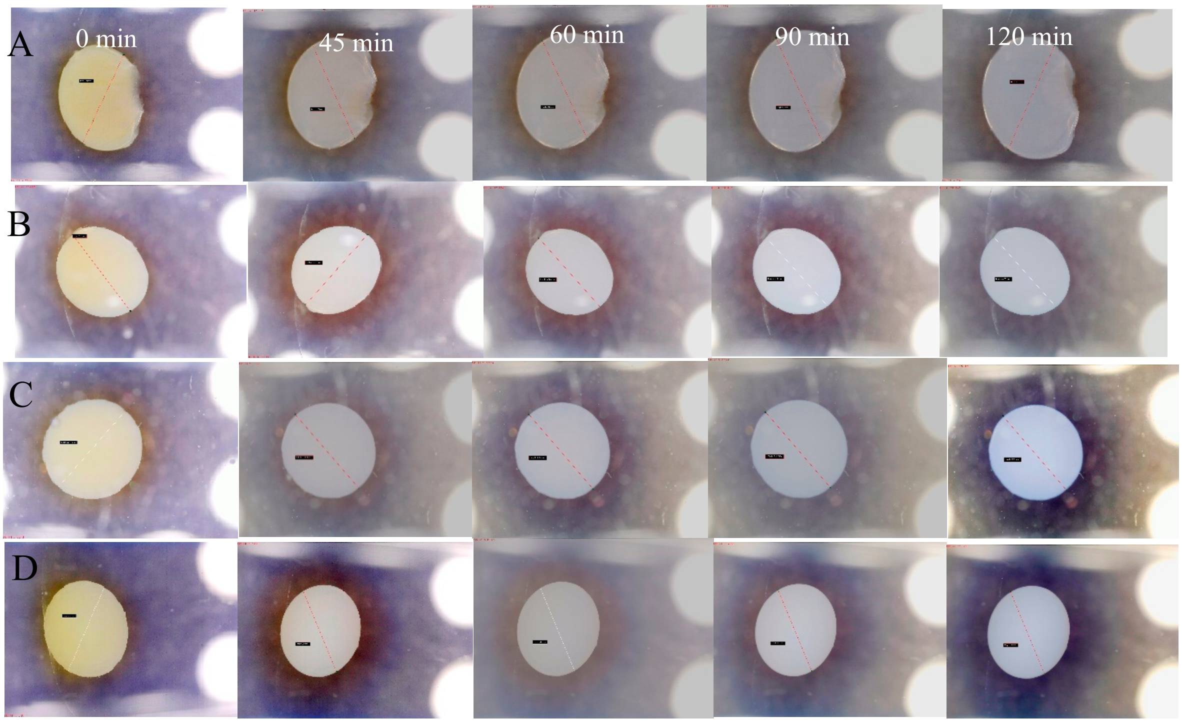

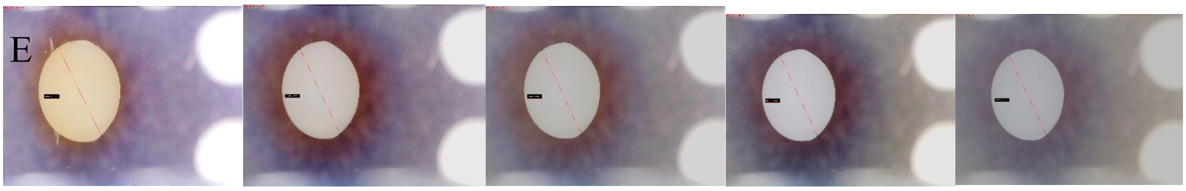

Supplementary Figure S42. Performance of alginate capsules containing Fricke dosimeter embedded in gelatine matrix stored for 0–120 min. Capsules formed in reaction of 3.5% sodium alginate with Fricke solution: 1 mM FAS, 50 mM.H_2SO_4, 0.165 mM XO containing 1–3.5% $CaCl_2$. Concentration of $CaCl_2$: 0 (A), 1 (B), 1.5 (C), 2.5 (D), and 3.5% (E) (storage temperature: ~23 °C).
